# Enteropathy produced in mice by intergenerational transmission of small intestinal microbiota from undernourished children

**DOI:** 10.1038/s41564-026-02394-4

**Published:** 2026-06-16

**Authors:** Kali M. Pruss, Clara Kao, Alexandra E. Byrne, Robert Y. Chen, Blanda Di Luccia, Laura Karvelyte, Reyan Coskun, Mackenzie Lemieux, Keshav Nepal, Daniel M. Webber, Matthew C. Hibberd, Yi Wang, Haoxin Liu, Dmitry A. Rodionov, Andrei L. Osterman, Marco Colonna, Christian Maueroder, Kodi Ravichandran, Michael J. Barratt, Tahmeed Ahmed, Jeffrey I. Gordon

**Affiliations:** 1 The Edison Family Center for Genome Sciences and Systems Biology, Washington University School of Medicine, St Louis, MO USA; 2 The Newman Center for Gut Microbiome and Nutrition Research, Washington University School of Medicine, St Louis, MO USA; 3 Division of Immunobiology, Department of Pathology and Immunology, Washington University School of Medicine, St Louis, MO USA; 4 Infectious and Inflammatory Disease Center, Sanford Burnham Prebys Medical Discovery Institute, La Jolla, CA USA; 5 Inflammation Research Centre, VIB, and the Department of Biomedical Molecular Biology, Ghent University, Ghent, Belgium; 6 International Centre for Diarrhoeal Disease Research, Bangladesh (icddr,b), Dhaka, Bangladesh

**Keywords:** Microbial communities, Microbiology, Immunology

## Abstract

Environmental enteric dysfunction (EED), a small intestinal disorder prevalent in undernourished children with stunted growth and their undernourished mothers, is associated with gut mucosal barrier disruption and decreased absorptive capacity. Here we provide preclinical evidence that intergenerational transmission of a perturbed small intestinal microbiota contributes to pathogenesis. One of two bacterial consortia cultured from duodenal aspirates obtained from Bangladeshi children with EED induced local and systemic inflammation in female gnotobiotic mice, resulting in impaired prenatal and postnatal growth in their offspring. Immunologic changes in pups phenocopied features of EED in children, and dam-to-pup transmission of this consortium altered signalling pathways related to intestinal epithelial cell renewal, barrier integrity and immune function. Screening of co-housed mice harbouring the inflammatory or non-inflammatory consortia identified *Campylobacter concisus* as an inducer of pro-inflammatory cytokine signalling in a host nitric oxide synthase-dependent manner. This preclinical model could facilitate small intestinal microbiota-targeted therapeutics for intergenerational undernutrition.

## Abstract (web summary)

In mouse models, small intestine microbiota of children suffering from environmental enteric dysfunction is transmitted maternally and recapitulates growth defects by modulation of inflammatory pathways

## Main

Undernutrition is a pressing global health challenge. Numerous epidemiologic studies indicate that stunting in mothers is associated with low birth weight and postnatal linear growth faltering (stunting) in offspring^1,2^. Stunting is accompanied by increased risk of infection, metabolic/hormonal imbalances later in life^3^, as well as neurodevelopmental/cognitive abnormalities^4–6^.

One hypothesis is that a perturbed small intestinal (SI) microbiota^7^ contributes to undernutrition by inducing environmental enteric dysfunction (EED), a subclinical disorder of the SI characterized by increased gut permeability, villus blunting and impaired nutrient absorption^7–13^. Transmission of the maternal SI microbiota to offspring may perpetuate intergenerational EED^14^. Evidence that the SI microbiota contributes to the pathogenesis of EED comes in part from the Bangladeshi EED (BEED) study, in which children who were stunted or at risk for stunting and failed to respond to a nutritional intervention underwent esophago-gastroduodenoscopy (EGD)^7,15^. Almost all (95%) displayed histopathologic evidence of EED^7^. Sequencing of 16S rRNA amplicons generated from their duodenal aspirates revealed a ‘core’ group of 14 bacterial taxa present in >80% of the children. The absolute abundances of these taxa were significantly negatively correlated with linear growth and positively correlated with levels of duodenal mucosal proteins involved in immunoinflammatory responses^7^.

Preclinical studies are necessary to test whether members of the microbiota are causally related to EED. Current mouse models of EED rely on either provision of a low-protein diet, which has not been explicitly defined as a causative factor for EED in humans, and/or administration of a single pathogen or broad inflammatory insult (for example, lipopolysaccharide)^16–19^. While these models are valuable for understanding effects of protein restriction on immunity and virulence mechanisms of enteric pathogens, they do not simulate the complexity of SI bacterial community dynamics and interactions with the host that probably play a role in pathophysiology of EED. In the current study, we characterize maternal-to-offspring transmission of different SI bacterial consortia from children in the BEED study that elicit discordant intestinal and systemic inflammatory responses in gnotobiotic mice. Our goal was to use these consortia to obtain insights about host cellular and molecular responses to members of the duodenal microbiota of children with EED and thus glean further understanding about the aetiology of this still enigmatic disorder.

## Results

### Modelling maternal–offspring transmission of EED donor SI bacterial consortia

We cultured and pooled 184 bacterial strains from the duodenal aspirates of children in the BEED study^7^. Due to ethical considerations, we could not perform EGD to obtain duodenal aspirates from healthy children. In addition, we chose a representative strain of each bacterial species present in the 184-member culture collection (based on their full-length 16S rRNA sequences, Supplementary Table 1b) and pooled the resulting 39 isolates into a species-representative subset consortium. We evaluated the physiologic effects of these two consortia in recently weaned mice (Supplementary Results). On the basis of higher levels of intestinal and systemic inflammation in mice colonized with the 184-isolate consortium, we named it ‘child small intestinal inflammation-inducing’ (cSI-I). The species-representative subset consortium was designated ‘child small intestinal non-inflammatory’ (cSI-N). cSI-N was used as a reference ‘control’ in addition to caecal contents from specific-pathogen-free mice.

**Fig. 1:**
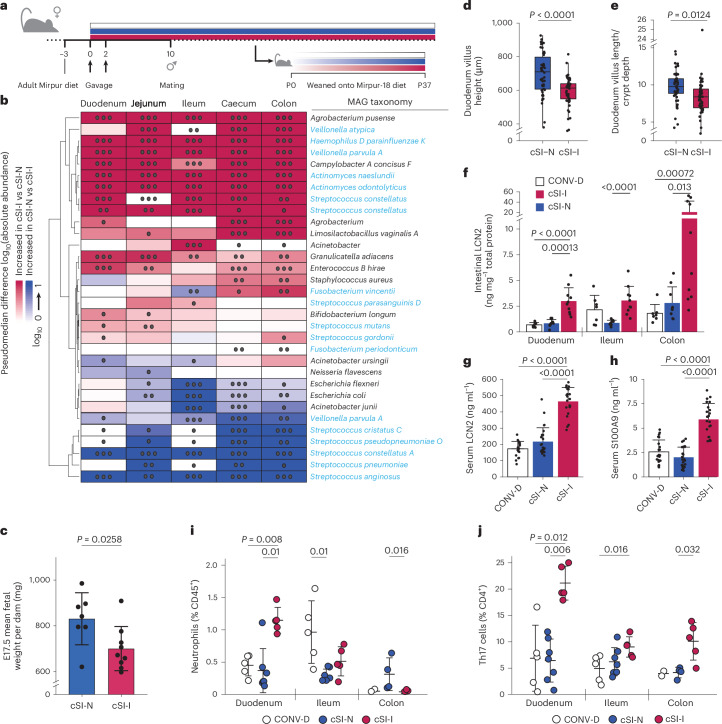
Dam-to-offspring transmission of bacteria cultured from duodenal aspirates collected from Bangladeshi children with EED. **a**, Design of intergenerational transmission experiment. **b**, Differential abundances of bacterial MAGs (rows) along the length of the intestine (columns) of P37 pups born to dams harbouring the cSI-N (*n* = 25 mice) or cSI-I bacterial consortia (*n* = 22 mice). MAGs were included in the heat map if they demonstrated significant differential log_10_ absolute abundance in at least one of the intestinal locations. MAG taxonomy coloured in blue indicates correspondence to ASVs representing ‘core taxa’ identified in the small intestines of stunted children with EED in the BEED study. ^•^*P*_adj_ < 0.05, ^••^*P*_adj_ < 0.01, ^•••^*P*_adj_ < 0.001; FDR-corrected two-sided Wilcoxon rank-sum tests. **c**, Average fetal mass per dam at E17.5. Dams were colonized with the cSI-I or cSI-N consortia for 2 weeks before mating (*n* = 7 cSI-N and 9 cSI-I dams; 2–10 fetuses per litter, two-sided unpaired *t*-test). **d**,**e**, Villus length (**d**) and ratio of villus length to crypt depth (**e**) in the duodenum of P37 cSI-I compared with cSI-N mice (*n* = 5 animals per group; boxes denote median and quartiles, whiskers denote the minimum and maximum within 1.5× the interquartile range). **f**, Levels of LCN2 protein in intestinal tissue from P37 offspring (*n* = 7 CONV-D, 9 cSI-N, 10 cSI-I). **g**,**h**, Serum levels of LCN2 (**g**) and S100A9 protein (**h**) in P37 pups (*n* = 21 CONV-D, 25 cSI-N, 22 cSI-I). **i**,**j**, Frequency of neutrophils (**i**) and Th17 cells (**j**) along the length of the gut (*n* = 5 CONV-D and cSI-I mice, *n* = 7 cSI-N for duodenum and ileum; *n* = 5 cSI-I, *n* = 4 cSI-N, *n* = 2 CONV-D mice for colon; colon from CONV-D animals was excluded from statistical comparisons). For **d**–**j**, statistics shown are the result of two-sided Wilcoxon rank-sum test; mean ± s.d. are shown.

**Extended Data Fig. 1:**
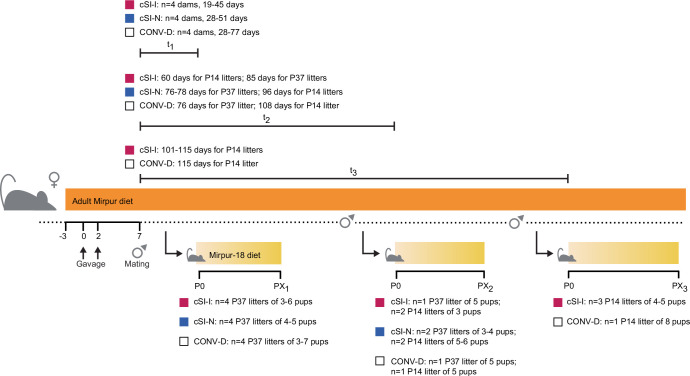
Intergenerational transmission experiment. Design of the intergenerational transmission experiment. Litter sizes and the age of offspring at the time of sample collection are shown for each dam (*n* = 4 dams/group). t_1_-t_3_ denotes the number of days between first mating and birth of the litter.

Germ-free (GF) adult female C57Bl/6J mice were fed a diet representative of that consumed by adults residing in the Mirpur district of Dhaka (‘Adult Mirpur’, Supplementary Table 1a). Three days later, animals were gavaged with the cSI-I or cSI-N consortia, or caecal contents from conventionally raised adult C57Bl/6J mice (‘conventionalized’, CONV-D; Fig. 1a and Extended Data Fig. 1). One week after colonization, female mice were mated with GF males. Pups were weaned onto a ‘Mirpur-18’ diet representative of that consumed by children living in Mirpur, where the BEED study was performed (Supplementary Table 1a). Animals consumed this diet ad libitum until euthanasia on postnatal day 37 (P37). To assess the reproducibility and timing of onset of EED features, second and third litters produced by the same dams were euthanized on P37 or P14, respectively (Extended Data Fig. 1).

We used metagenome-assembled genomes (MAGs) to identify bacterial members of each consortium that successfully colonized dams and their offspring (Supplementary Results). The absolute abundances of 21 MAGs were significantly higher in at least one intestinal segment of P37 offspring colonized with the cSI-I compared with cSI-N consortium; the taxonomy of 12 of these 21 MAGs corresponded to strains we previously identified as ‘core taxa’ (amplicon sequence variants, ASVs) present in >80% of the duodenal microbiota of Bangladeshi children with biopsy-confirmed EED (Fig. 1b)^7^.

### Reduced prenatal and postnatal growth in cSI-I offspring

**Extended Data Fig. 2:**
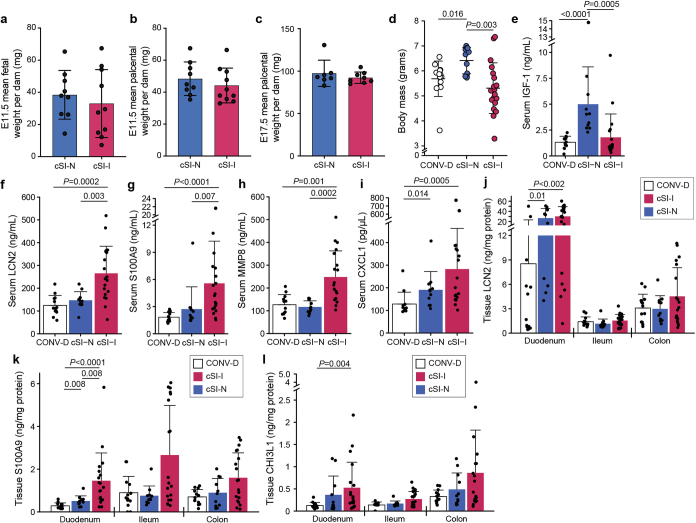
Growth and inflammatory phenotypes of P14 offspring and fetuses. **a**, Average masses of fetuses per dam at E11.5 (*n* = 9 cSI-N dams, 10 cSI-I dams; *n* = 4–8 fetuses/litter). **b,c**, Average masses of placentas within each dam at E11.5 (**b**) and E17.5 (**c**) (*n* = 9 cSI-N dam at E11.5 and 7 at E17.5, *n* = 10 cSI-I dams at E11.5 and 8 at E17.5; *n* = 2–10 conceptuses/litter). For **a-c**, *P* values determined with two-sided unpaired t-tests. Each point represents averaged data for one dam. **d**, Body mass of P14 offspring of dams colonized with the cSI-I consortium (red), cSI-N consortium (blue), or cecal contents from a conventionalized mouse (white, CONV-D). **e**, Serum levels of IGF-1 in P14 offspring. **f-i**, Serum levels of the inflammation-associated proteins, LCN2 (**f**), S100A9 (**g**), MMP8 (**h**), and CXCL1 (**i**), in P14 offspring. **j-l**, Levels of the inflammation-associated proteins LCN2 (**j**), S100A9 (**k**), and CHI3L1 (**l**) in duodenal, ileal, and colonic tissue of P14 animals. For **d-l**, *n* = 11 cSI-N P14 offspring, *n* = 19 cSI-I, *n* = 13 CONV-D (3–8 pups/litter). *P* values were determined using two-sided Wilcoxon rank-sum with Tukey’s post-hoc tests. For all panels, mean values ± s.d. are shown.

We colonized a separate group of adult female mice with the cSI-I or cSI-N consortia 2 weeks before mating. Pregnant dams were euthanized when placentation just completed (embryonic day 11.5, E11.5) or near the end of gestation (E17.5). Fetal weights were significantly lower in cSI-I compared with cSI-N dams at E17.5 (Fig. 1c), but not at E11.5 (Extended Data Fig. 2a and Supplementary Table 2a). Placental weights were not significantly different between groups (Extended Data Fig. 2b,c and Supplementary Table 2a). Body mass (Extended Data Fig. 2d) and serum levels of IGF-1 (Insulin-like growth factor 1, Extended Data Fig. 2e) were significantly reduced in pre-weaning (P14) offspring of cSI-I compared to cSI-N dams (Supplementary Table 2a,b).

### Pro-inflammatory biomarkers identified in children with EED in cSI-I dams and their offspring

P14 represents a critical period of lymphoid organ and adaptive immune system maturation. P14 cSI-I pups displayed significantly higher serum levels of pro-inflammatory proteins lipocalin-2 (LCN2), S100 calcium binding protein A9 (S100A9), chitinase-3-like protein 1 (CHI3L1) and matrix metallopeptidase 8/neutrophil collagenase (MMP8), as well as the immune cell chemoattractant chemokine ligand 1 (CXCL1) compared with cSI-N and CONV-D pups (Extended Data Fig. 2f–i and Supplementary Table 2b). Levels of LCN2, CHI3L1, S100A9 and MMP8 in duodenal biopsies were significantly positively correlated with the abundances of the ‘core’ EED-associated bacteria in children from the BEED study^7^. LCN2, S100A9 and CHI3L1 were similarly elevated in duodenal, ileal and colonic tissue of cSI-I P14 pups (Extended Data Fig. 2j–l and Supplementary Table 2b). Thus, systemic and intestinal inflammation were evident before completion of weaning.

**Extended Data Fig. 3:**
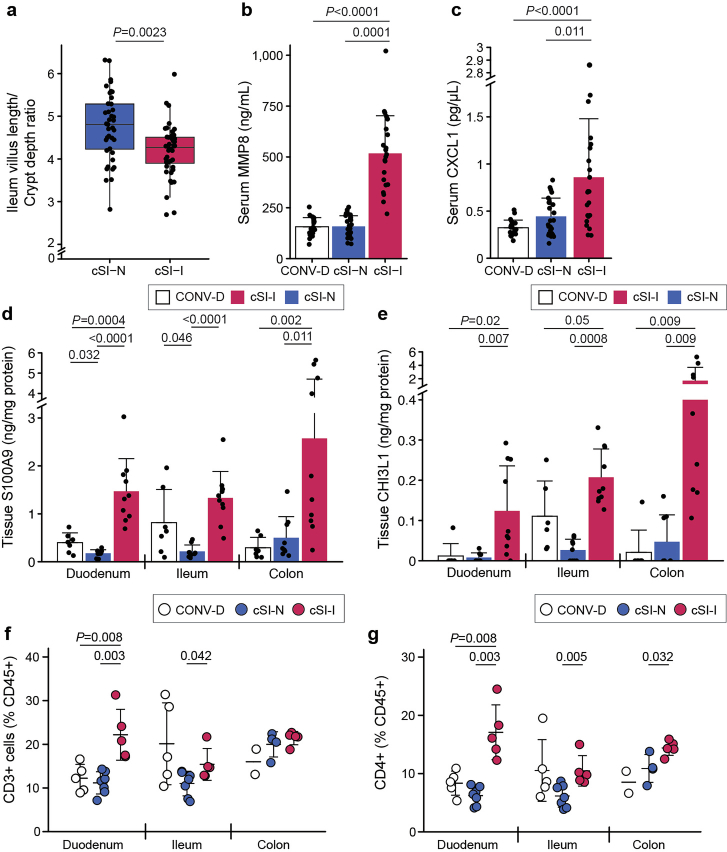
Assessment of EED biomarkers and immune profiling of P37 animals. **a**, Ratio of villus length to crypt depth in the ileums of cSI-I and cSI-N mice (*n* = 5 mice per group, two-sided Wilcoxon rank-sum test; boxes denote median and quartiles, whiskers indicate the minimum and maximum within 1.5 times the interquartile range). **b,c**, Levels of serum MMP8 (**b**), and CXCL1 (**c**), in the P37 offspring of C57Bl/6J dams colonized with the cSI-I or cSI-N consortia or conventionalized with caecal microbiota harvested from conventionally-raised C57Bl/6J animals (abbreviated CONV-D) (*n* = 21 CONV-D P37 offspring, *n* = 22 cSI-I, *n* = 25 cSI-N). **d,e**, Levels of S100A9 (**d**), and CHI3L1 (**e**), in the duodenal, ileal, and colonic tissues of P37 animals (*n* = 7 CONV-D mice, *n* = 9 cSI-N, *n* = 10 cSI-I). **f,g**, Frequency of CD3^+^ (**f**), and CD4^+^ (**g**) immune cells in the duodenal, ileal and colonic lamina propria of P37 offspring (*n* = 5 P37 CONV-D and cSI-I offspring for the duodenum and ileum, *n* = 7 cSI-N for the duodenum and ileum, *n* = 4 cSI-N, *n* = 5 cSI-I and *n* = 2 CONV-D for the colon; colonic tissue from CONV-D mice was excluded from statistical comparisons). For panels **b-g**, bars denote mean ± s.d. and *P*-values were determined by two-sided Wilcoxon rank-sum tests.

To determine whether the effects of the cSI-I and cSI-N consortia persisted through the weaning period, we assessed offspring on P37, which represents a developmental state akin to adolescence^20^. Children with EED have villus blunting: histomorphometric analyses of the small intestines of P37 offspring of cSI-I dams revealed significantly diminished duodenal villus height (Fig. 1d) and ratio of villus height to crypt depth in the duodenum and ileum compared to cSI-N offspring (Fig. 1e, Extended Data Fig. 3a and Supplementary Table 2c). Crypt depth in the ileum of P37 cSI-I mice tended to increase relative to that of cSI-N animals (*P* = 0.09, two-sided Wilcoxon rank-sum). Increased crypt depth is consistent with a proliferative response involving stem and transit-amplifying cells, but one that is insufficient to restore villus height (Supplementary Results).

LCN2 levels in serum as well as in duodenal, ileal and colonic tissue were significantly elevated in P37 mice born to cSI-I compared to cSI-N and CONV-D dams (Fig. 1f,g). S100A9, MMP8, CXCL1 and IL-17 were similarly elevated in the serum of P37 cSI-I offspring (Fig. 1h, Extended Data Fig. 3b,c and Supplementary Table 2b). As in P14 animals, duodenal, ileal and colonic tissue levels of S100A9 and CHI3L1 were significantly higher in P37 offspring of cSI-I dams (Extended Data Fig. 3d,e and Supplementary Table 2b). There were no statistically significant differences in the serum levels of these proteins between litters, nor between male and female offspring within the same colonization condition (*P* > 0.05, Wilcoxon rank-sum tests).

Levels of proteins involved in bone remodelling and SI tissue acylcarnitines were elevated in cSI-I P37 offspring, indicating increased osteocyte activity and perturbed carnitine shuttle activity (Supplementary Results).

### Alterations in immune cells in the intestinal lamina propria

The proportion of neutrophils among CD45^+^ cells was significantly increased in the duodenum of P37 cSI-I offspring, but not in their ileum or colon (Fig. 1i). CD3^+^ and CD4^+^ cells were also higher in their duodenum, ileum and colon compared with cSI-N or CONV-D offspring (Extended Data Fig. 3f,g). Among CD4^+^ cells, the Th17 population was significantly higher in the duodenum, ileum and colon of cSI-I offspring (Fig. 1j). Th1 cells were diminished in the duodenum, and Tregs were diminished in the duodenum and colon (Supplementary Table 2f). These findings lead us to speculate that a pro-inflammatory cytokine milieu in the intestines of P37 cSI-I offspring promotes Th17 cell differentiation and proliferation.

The above protein biomarkers, neutrophil and T cell phenotypes were also evident in dams: the frequencies of duodenal neutrophils, CD3^+^ cells, CD4^+^ T cells and Th17 cells were all significantly elevated in cSI-I adult females, while their Th1 cells and Tregs were significantly reduced (*P* < 0.05 for all comparisons, Wilcoxon rank-sum test; *n* = 5 mice per group; Supplementary Table 2f). The increased CD3^+^ and CD4^+^ T cells in the intestines of cSI-I compared to cSI-N dams and their offspring is consistent with previous characterization of EED in children as a T cell mediated enteropathy^12,21^.

### Intestinal epithelial cellular responses

To investigate intestinal epithelial responses to dam–pup transmission of the cSI-I and cSI-N consortia, we performed single nucleus RNA-sequencing (snRNA-seq) on duodenal and ileal tissue of P37 offspring. Expression of genes involved in tissue proliferation and pro-inflammatory innate immune pathways, as well as genes corresponding to duodenal mucosal proteins in children with EED that were negatively correlated with healthy growth, were elevated in cSI-I offspring (Supplementary Results).

### Co-housing to nominate pathology-inducing bacterial strains

**Fig. 2:**
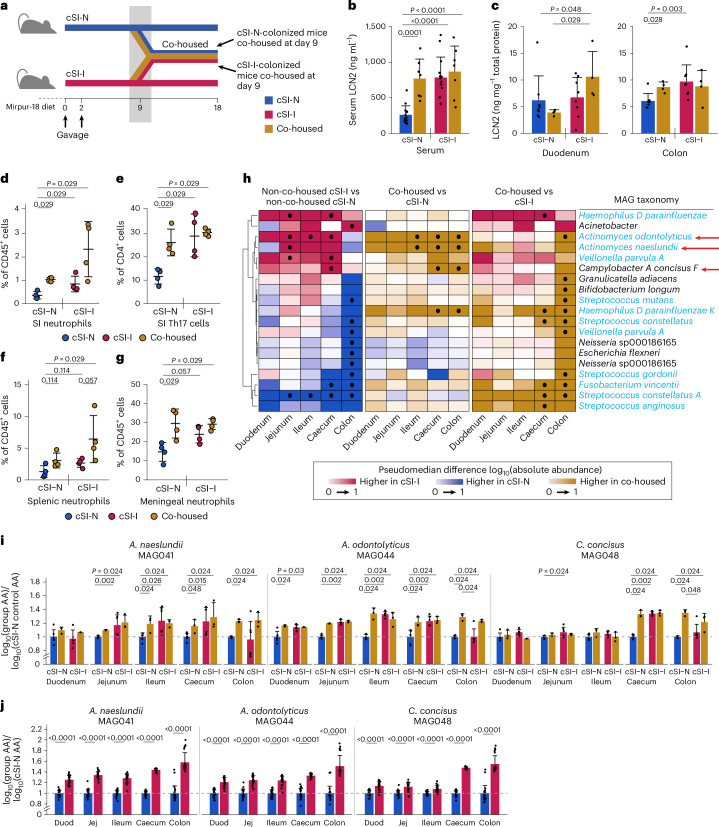
Identification of bacteria associated with pathology after co-housing. **a**, Experimental design of co-housing experiment. **b**, Serum levels of LCN2 protein on experimental day 18, 9 days after initiation of co-housing; *n* = 14 mice (control group), *n* = 7 mice (co-housed group). **c**, Intestinal tissue levels of LCN2 measured on experimental day 18, 9 days after co-housing; *n* = 8 mice (control group), *n* = 4 mice (co-housed group). **d**,**e**, Frequency of neutrophils (**d**) and Th17 cells (**e**) in the small intestinal lamina propria (*n* = 4 mice per group; duodenal, jejunal and ileal segments were combined before analysis). **f**,**g**, Frequency of neutrophils in the spleen (**f**) and meninges (**g**) (*n* = 4 mice per group). For **d**–**f**, each point represents an individual animal; the colour code matches that used in **a**. **h**, Differences in absolute abundances of MAGs along the length of the gut (*n* = 6 mice per group). MAGs were included if they demonstrated statistically significant differences in their absolute abundance for any comparison between groups in at least one segment of the intestine. MAG taxonomy coloured in blue indicates correspondence to ASVs representing ‘core taxa’ in the duodenal microbiota of stunted Bangladeshi children with EED in the BEED study. The three red arrows point to MAGs defined as ‘pathology associated’. ^•^*P*_adj_ < 0.05, FDR-corrected, two-sided Wilcoxon rank-sum test. **i**,**j**, Log_10_ absolute abundances (AA) of the pathology-associated MAGs along the length of the intestine in either the co-housing experiment (**i**) or P37 animals from the intergenerational experiment (**j**). Values are expressed relative to cSI-N non-co-housed controls (**i**) or P37 cSI-N offspring in the intergenerational transmission experiment (**j**, *n* = 22 cSI-I mice, 25 cSI-N). For **b**–**g**,**i** and **j**, *P* values were determined using two-sided Wilcoxon rank-sum tests; mean ± s.d. are shown. For **b**–**i**, data shown are from 2 independent experiments (*n* = 6 or 8 mice per group).

To identify bacteria that conferred or ameliorated the immunoinflammatory state described above, we gavaged separate groups of 4–5-week-old GF C57Bl/6J mice consuming the Mirpur-18 diet with the cSI-I or cSI-N consortia. After 9 days, half of the animals from each group were co-housed; protein biomarkers and immune cell populations were evaluated 9 days later (Fig. 2a). Co-housed mice and non-co-housed cSI-I controls gained significantly less weight compared to non-co-housed cSI-N controls (linear mixed-effects model; Supplementary Table 2b). Irrespective of the initial colonization, serum concentrations of LCN2 and CHI3L1 were significantly higher in co-housed compared with non-co-housed cSI-N and did not differ significantly from non-co-housed cSI-I animals (Fig. 2b and Supplementary Table 2b). Levels of LCN2 and CHI3L1 were significantly higher in colonic tissue and trended higher in duodenal tissue from co-housed compared with cSI-N mice (Fig. 2c and Supplementary Table 2b).

Neutrophils and Th17 cells were significantly higher in the SI lamina propria of cSI-I and co-housed animals (regardless of initial colonization) compared with cSI-N; Th1 cells were reduced (Fig. 2d,e and Supplementary Table 2f). Splenic and meningeal neutrophils were similarly elevated in cSI-I and co-housed compared to cSI-N mice (Fig. 2f,g and Supplementary Table 2f). There was no evidence for a mitigating effect of the cSI-N community on pathology in mice originally colonized with cSI-I (Supplementary Tables 2a,f). These results suggested either restructuring of strain composition within the cSI-N consortium or transfer of bacteria from cSI-I to cSI-N animals after co-housing, inducing pathology in these formally healthy animals.

We again performed shotgun sequencing of intestinal contents, seeking bacterial taxa whose abundances were elevated in the ‘inflammatory’ experimental groups (cSI-I and co-housed mice) compared to their ‘non-inflammatory’ counterparts (cSI-N). Three MAGs satisfied these criteria: *Actinomyces naeslundii* (MAG041), *Actinomyces odontolyticus* (MAG044) and *Campylobacter concisus* (MAG048) (Fig. 2h and Supplementary Table 1d).

Compared with cSI-N controls, the absolute abundance of *C. concisus* was significantly higher in the caecum and colon of co-housed animals, regardless of initial colonization condition (Fig. 2i). The absolute abundances of *A. naeslundii* and *A. odontolyticus* were significantly higher in the jejunum, ileum, caecum and colon of co-housed compared with non-co-housed cSI-N mice (Fig. 2i). Abundances of all three MAGs were equivalent in co-housed and cSI-I mice in the SI and caecum (Fig. 2i), suggesting immigration of these taxa from cSI-I to cSI-N animals during co-housing. Moreover, their absolute abundances were significantly increased along the length of the gut of P37 cSI-I compared to cSI-N offspring in our intergenerational transmission model (Fig. 2j and Supplementary Table 1d). *C. concisus* and *Actinomyces* spp. are resident members of the oral microbiota^22^ and are not typically considered pathogens, although some strains have been associated with periodontitis and inflammatory bowel disease^23–25^.

Four isolates in our clonally arrayed culture collection matched these three MAGs that were elevated in co-housed animals compared to cSI-N controls, with an average nucleotide identity (ANI) score of >99%: two isolates matched the *A. naeslundii* MAG041 (Bg041a, Bg041b), one matched *A. odontolyticus* MAG044 (Bg044) and one matched *C. concisus* MAG048 (Bg048) (Supplementary Table 4a; see Supplementary Table 4b for results of in silico metabolic reconstructions of these pathology-associated MAGs, their corresponding cultured isolates and other phylogenetically related species^26,27^).

### Testing the role of isolates corresponding to pathology-associated MAGs

**Fig. 3:**
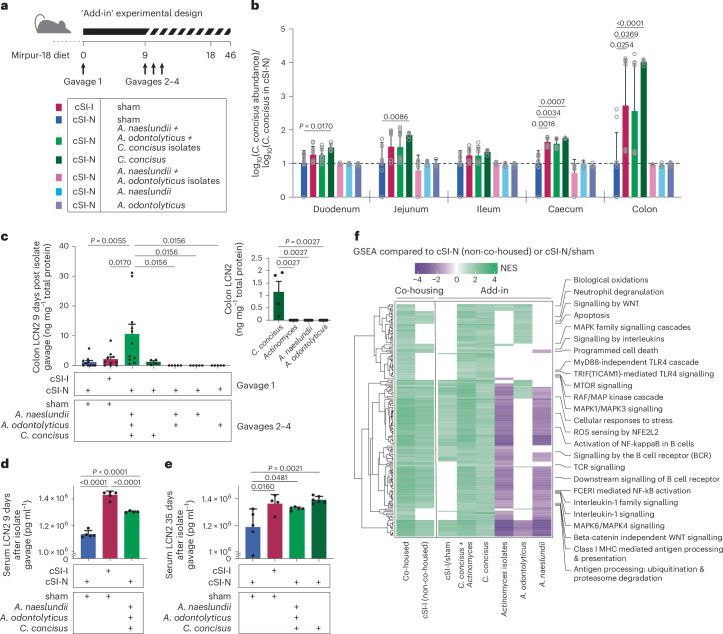
Direct test of candidate pathology-inducing small intestinal bacterial strains in cSI-N mice. **a**, Experimental design of ‘add-in’ experiment. Individual cultured isolates, alone or in combinations, were gavaged once daily on days 9–11 into mice previously colonized with the cSI-N bacterial consortium. **b**, Ratio of the absolute abundance of *C. concisus* strain Bg048 (corresponding to MAG048) shown relative to its absolute abundance in cSI-N/sham controls (a ratio of 1 is indicated by the dotted line; *F*_(24,176)_ = 5.572, *P* < 0.0001, two-way analysis of variance (ANOVA) with Dunnett’s multiple comparisons to cSI-N/sham control; mean ± s.d. are shown). Colours correspond to the groups shown in **a**. **c**, Levels of LCN2 in colonic tissue measured on experimental day 18, 9 days after isolate gavage (*F*_(6,43)_ = 4.481, *P* = 0.0013). The inset shows colonic LCN2 levels after addition of the individual isolates alone (*F*_(3,15)_ = 9.054, *P* = 0.0012, one-way ANOVAs with Tukey’s multiple comparisons; mean ± s.e.m. are shown). **d**,**e**, Serum LCN2 9 days (**d**, *F*_(2,11*)*_ = 184.1, *P* < 0.0001) or 35 days (**e**, *F*_(3,16)_ = 7.273, *P* = 0.0027) following isolate gavage (mean ± s.d. shown, one-way ANOVAs with Tukey’s multiple comparisons). **f**, Results of bulk RNA-seq analysis: normalized enrichment scores (NES) for all reactome pathways that were significantly enriched in the colon (GSEA *q* < 0.05) of the indicated treatment groups compared to their respective cSI-N counterparts (non-co-housed cSI-N controls or cSI-N/sham). Pathways related to the immune system are labelled. Pathways were included in the heat map if they were significantly enriched with the addition of *C. concisus* and in co-housed animals. If a pathway was not significantly enriched, it was assigned a NES score of zero. For **b**, **c** and **f**, data were combined across 2 independent experiments with *n* = 10 cSI-I/sham and cSI-N/sham animals, *n* = 11 cSI-N/*C. concisus* + *A. odontolyticus* + *A. naeslundii* and *n* = 5 cSI-N/*C. concisus*, cSI-N/*A. odontolyticus*, cSI-N/*A. naeslundii* and cSI-N/*A. naeslundii* + *A. odontolyticus* mice. For **d** and **e**, a third independent experiment involved *n* = 5 mice per group except for cSI-N/*C. concisus* + *A. odontolyticus* + *A. naeslundii* at 9 days and cSI-I/sham at 35 days where *n* = 4, and cSI-N/*C. concisus* at 35 days where *n* = 6.

We directly tested the capacity of these Bangladeshi *C. concisus, A. odontolyticus* and *A. naeslundii* isolates to induce pathology. GF 4–5-week-old C57Bl/6J mice consuming the Mirpur-18 diet were gavaged with the cSI-N consortium (experimental day 0). On days 9 through 11, mice were gavaged once daily with either (1) a pool of all four isolates, (2) the *C. concisus* isolate, (3) the two *A. naeslundii* isolates, (4) the *A. odontolyticus* isolate or (5) a pool of both *Actinomyces* species. As positive and negative controls, mice initially colonized with the cSI-N or cSI-I consortium were subsequently gavaged with uninoculated culture medium (‘sham’, Fig. 3a).

**Extended Data Fig. 4:**
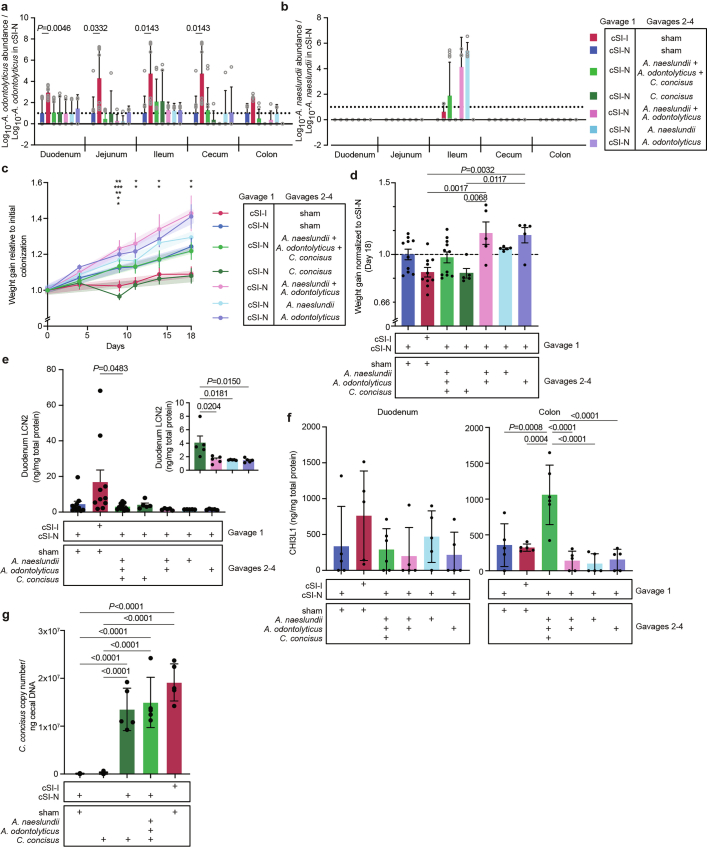
Colonization status, weight gain and inflammatory biomarkers in mice from the isolate ‘add-in’ experiments. **a,b**, Animals were initially colonized with either the cSI-N or cSI-I consortium (‘gavage 1’). Gavages 2–4 were performed on experimental days 9 through 11, as in Fig. 4a. The absolute abundances of *A. odontolyticus* isolate Bg044 (corresponding to MAG044, panel **a**) and *A. naeslundii* isolate Bg041 (corresponding to MAG041, panel **b**), shown relative to their absolute abundance in the cSI-N/sham-gavaged controls (dotted line indicates ratio of 1; mixed-effects analysis with adjusted *P*-values from Dunnett’s multiple comparisons to cSI-N/sham control are shown; F_(6,44)_ = 6.439, *P* < 0.0001 for treatment group; F_(17.22,113.4)_ = 1.417, *P* = 0.1405 for group ~ intestinal tissue; mean values ± s.d. are shown). **c**, Changes in body mass relative to time of colonization in mice in ‘add-in’ experiments (points and error bars denote mean values ± s.e.m.; shaded, thicker line represents results of linear regression, F_(6,292)_ = 7.577, *P* < 0.0001. *P < 0.05, **P < 0.01, ***P < 0.001 from Tukey’s multiple comparisons demonstrating significant differences between *C. concisus*-gavaged animals compared to cSI-N/mock, cSI-N/*A.naeslundii*, *A. odontolyticus*, or *Actinomyces* from two-way repeated measures ANOVA; full statistics are reported in Supplementary Table 2b). **d**, Relative weight gain in isolate-gavaged and cSI-I/mock-gavaged animals relative to weight gain in cSI-N mice (F_(6,44)_ = 5.604, *P* = 0.0002, one-way ANOVA with Tukey’s post-hoc, mean ± s.e.m.). **e**, Levels of LCN2 protein in duodenal tissue on experimental day 18 (F_(6,44)_ = 2.569, *P* = 0.0321). The inset shows comparisons between individual isolates (F_(3,16)_ = 5.752, *P* = 0.0072, one-way ANOVAs with Tukey’s multiple comparisons; mean values ± s.e.m. are shown). **f**, Tissue levels of CHI3L1 (Chitinase-3-like protein 1) in the duodenum (F_(5,25)_ = 1.178, *P* = 0.3479) and colon (F_(5,25)_ = 12.73, *P* < 0.0001, one-way ANOVAs with Tukey’s multiple comparisons, *n* = 5 mice/group except for cSI-N/*C. concisus* + *A. odontolyticus* + *A. naeslundii* where *n* = 6, mean ± s.d. shown). For **a-e**, *n* = 10 cSI-N/sham and cSI-I/sham, *n* = 11 cSI-N/*C. concisus* + *A. odontolyticus* + *A. naeslundii*, *n* = 5 cSI-N/*C. concisus*, cSI-N/*A. odontolyticus*, cSI-N/*A. naeslundii*, and cSI-N/*Actinomyces*. **g**, Abundance of *C. concisus* in caecal contents of mice mono-colonized (gavaged on days 0, 3, 5, 7, and 14, n = 7 mice) compared to mice from the ‘add-in’ experiment (F_(4,22)_ = 38.49, *P* < 0.0001, one-way ANOVA with Tukey’s multiple comparisons, mean ± s.d. shown).

Secondary gavage with the four isolates combined or *C. concisus* alone led to increased absolute abundance of *C. concisus*, most notably in the caecum and colon, compared to sham-gavaged cSI-N controls (Fig. 3b). The levels of *C. concisus* achieved over the course of the 9 days following its introduction into cSI-N mice were comparable to its levels in cSI-I sham-gavaged controls (Fig. 3b) as well as co-housed animals (Supplementary Table 1d). In contrast, gavage with the *Actinomyces* species either alone or in concert resulted in a trending, but not statistically significant, increase in their absolute abundance along the length of the intestinal tract compared to cSI-N sham-gavaged controls (Extended Data Fig. 4a,b and Supplementary Table 1d).

Mice colonized with cSI-N and then gavaged with *C. concisus* gained significantly less weight than cSI-N sham-gavaged controls or animals gavaged with each of the two *Actinomyces* strains alone or together (Extended Data Fig. 4c,d). Colonic inflammation, assessed by LCN2 levels, was significantly higher when all four isolates were gavaged together into cSI-N mice compared with all other treatment groups (Fig. 3c). This increase in LCN2 levels did not occur in the duodenum (Extended Data Fig. 4e). Similar patterns were observed for the biomarker CHI3L1 (Extended Data Fig. 4f). These results suggested that in the distal gut, the presence of *A. odontolyticus* and *A. naeslundii* directly or indirectly affected *C. concisus* in a manner that increased its capacity to induce inflammation.

Systemic inflammation, quantified by serum LCN2, was significantly higher 9 days after gavage of the four strains together compared with cSI-N controls, but remained lower than in cSI-I controls (Fig. 3d). In a follow-up experiment, serum LCN2 was assessed 35 days after isolate gavage; introduction of either the four isolates together or *C. concisus* alone was sufficient to produce LCN2 levels that were significantly higher than that in cSI-N controls and not significantly different from that in cSI-I mice (Fig. 3e and Supplementary Table 2b). Furthermore, in mono-colonized mice, *C. concisus* did not reach levels higher than that in cSI-N controls and did not induce intestinal inflammation (Extended Data Fig. 4g and Supplementary Results). Together, these data suggest that, given a sufficient duration of colonization within the context of the cSI-N community, *C. concisus* induces levels of systemic inflammation comparable to that produced by the cSI-I consortium.

### *C. concisus*-induced inflammatory signalling in the intestinal epithelium

We conducted bulk RNA-seq of the duodenum and colon to further understand the host response to the presence of *C. concisus*. We compared reactome pathways whose expression was significantly enriched compared to cSI-N controls in (1) P37 cSI-I animals, (2) co-housed animals and non-co-housed cSI-I controls and (3) mice initially colonized with cSI-N and subsequently gavaged with *C. concisus*, *A. odontolyticus* or *A. naeslundii* individually, both *Actinomyces* species, all isolates together, as well as cSI-I controls (Supplementary Table 3d).

*C. concisus*, either alone or with the *Actinomyces* species, induced a pro-inflammatory transcriptional response in the colon that was not present in the duodenum (Supplementary Table 3d). Compared with their cSI-N counterparts, reactome pathways involved in recruitment and activation of immune cells, reactive oxygen species generation and detoxification, antigen presentation, MAPK signalling, pro-inflammatory cytokines and toll-like receptor (TLR) signalling were more highly expressed in the colons of P37 cSI-I animals, co-housed and non-co-housed cSI-I controls, and recipients of *C. concisus* (±*Actinomyces*) (Fig. 3f and Supplementary Table 3d). Genes involved in cytokine (IL-1) signalling leading to programmed cell death (apoptosis) and immune cell activation (neutrophil degranulation) were induced in the colon following gavage of *C. concisus* alone or with the *Actinomyces* isolates (Supplementary Table 3d). Of the 41 immunoinflammatory pathways significantly enriched in the colons of mice gavaged with *C. concisus* and the *Actinomyces* spp., only five were enriched in the duodenum (Supplementary Table 3d). None of the 49 pathways enriched in the colon after gavage of *C. concisus* alone were enriched in the duodenum (Supplementary Table 3d). In the absence of *C. concisus*, the *Actinomyces* isolates did not elicit a significant inflammatory transcriptional response in either the colon or the duodenum (Fig. 3f and Supplementary Table 3d). Together, these findings provide evidence that this *C. concisus* isolate induced immunoinflammatory gene expression in the context of the cSI-N community that recapitulated aspects of that induced by the full EED donor-derived cSI-I consortium.

### *C. concisus* pathogenesis

**Fig. 4:**
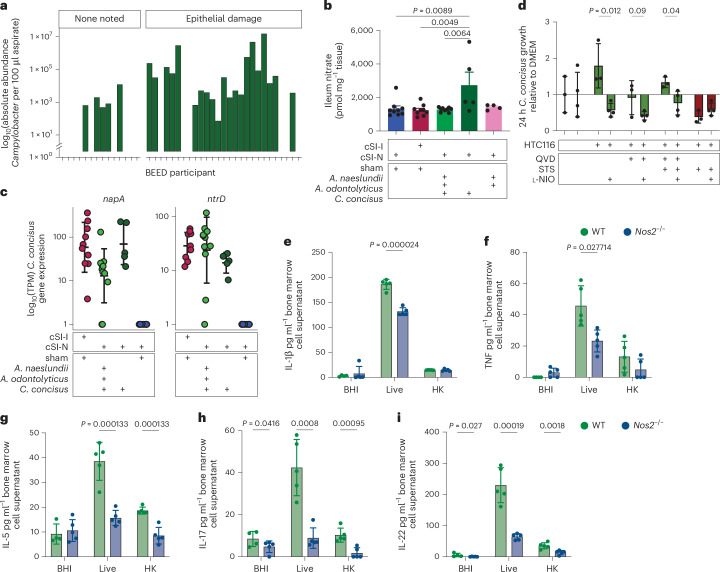
Colonic epithelial nitric oxide synthase activity confers growth advantage to *C. concisus*, which elicits cytokine signalling in a *Nos2*-dependent manner. **a**, Absolute abundance of the *Campylobacter* ASV identified in duodenal aspirates from children in the BEED study (*n* = 41). **b**, Levels of nitrate in homogenized ileal tissue 9 days post isolate gavage (*F*_(4,31)_ = 4.576, *P* = 0.0051, one-way ANOVA with Tukey’s multiple comparisons; *n* = 9 mice for cSI-I, cSI-N and cSI-N/*C. concisus* + *A. odontolyticus* + *A. naeslundii*, *n* = 5 for cSI-N/*C. concisus*, *n* = 4 for cSI-N/*Actinomyces*. Bars denote mean ± s.e.m. **c**, Expression of *C. concisus* nitrate reductase (*napA*) and nitrate transporter (*ntrD*) 9 days post isolate gavage, or in cSI-I and cSI-N sham-gavaged controls (tpm, transcripts per million, mean ± s.d.; *n* = 10 cSI-I and cSI-N, *n* = 11 cSI-N/*C. concisus* + *A. odontolyticus* + *A. naeslundii*, and *n* = 5 cSI-N/*C. concisus* mice). **d**, Comparison of *C. concisus* growth after 24 h in spent medium collected from live human colonic cells (HTC116 ± QVD, QVD + STS; green) or apoptotic cells (STS, red) that were or were not treated with the NOS inhibitor L-NIO compared to growth in cell culture medium alone (DMEM, *n* = 3 biological replicates per condition without L-NIO treatment, 4 per condition with L-NIO; pairwise two-tailed *t*-test for each ±L-NIO comparison. Bars denote mean ± s.d., see also Supplementary Results). **e**–**i**, Bone marrow cells collected from conventionally raised wild-type (WT) or iNOS^−/*−*^ (*Nos2*^−/*−*^) mice were incubated with live or heat-killed (HK) *C. concisus* for 48 h. Cytokines were quantified in bone marrow cell supernatants (*n* = 4 biological replicates per WT control (BHI), *n* = 5 for all other treatment groups; mean ± s.d., multiple two-sided unpaired *t*-tests with two-stage step-up FDR correction).

Returning to our V4-16S rRNA dataset from the duodenal aspirates of children in the BEED study^7^, we found that the prevalence of the only *Campylobacter* ASV present (undefined species) was significantly higher in children with ‘epithelial damage’ defined by histopathologic scoring metrics^2^ (Fig. 4a, two-sided Fischer’s exact test *P* = 0.0057, no adjustment for multiple hypotheses). The absolute abundance of this *Campylobacter* ASV was significantly positively correlated with levels of only one protein in the duodenal mucosa—CD177, also known as human neutrophil antigen 2 (HNA-2) (Pearson correlation, *R* = 0.694, *P*_adj_ = 0.00692). CD177/HNA-2 plays a critical role in neutrophil transmigration and extravasation during an inflammatory response^28,29^.

To nominate potential mechanisms by which *C. concisus* may elicit host inflammation, we (1) performed a comparative genomics analysis using 119 published genomes and (2) analysed *C. concisus* gene expression during the ‘add-in’ experiment described above. These analyses (Supplementary Results) suggested that *C. concisus* may utilize host-derived substrates in the gut. To test this hypothesis, we turned to an ex vivo tissue culture system in which we collected conditioned culture medium from live or apoptotic mouse and human colonic epithelial cells and found that spent medium from live cells increased *C. concisus* growth (Supplementary Results).

Combining results from microbial and host RNA-seq, we postulated that *C. concisus* may utilize products of host nitric oxide metabolism. Nitrate was significantly elevated in ileal tissue of *C. concisus*-gavaged animals (Fig. 4b) and a *C. concisus* nitrate reductase was more highly expressed in inflammatory conditions (Fig. 4c and Supplementary Results). Moreover, treating human colonic epithelial cells with the nitric oxide synthase inhibitor *N*^5^-(1-Iminoethyl)-L-ornithine (L-NIO) abrogated the growth benefit to *C. concisus* (Fig. 4d, and Supplementary Table 6a and Results).

### *C. concisus*-induced inflammatory cytokine signalling is enhanced by iNOS

**Extended Data Fig. 5:**
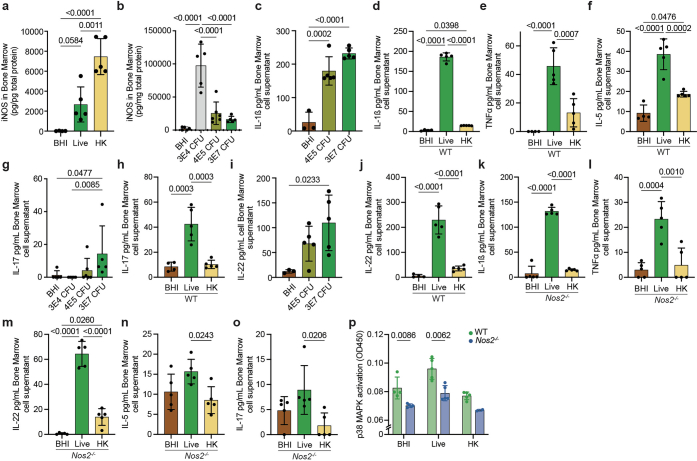
*C. concisus* induces iNOS and elicits iNOS-dependent immune signaling. **a,b**, Bone marrow cells from wild-type (WT) mice were incubated for 48-hours with fresh (Live) or heat-killed (HK) *C. concisus* culture, or bacterial medium as a negative control (BHI, F_(2,11)_ = 28.24, *P* < 0.0001). **b**, Bone marrow cells were co-cultured for 48-hours with increasing amounts of live *C. concisus* culture. Proteins were extracted from bone marrow cell pellets and iNOS levels were quantified (F_(3,17)_ = 26.71, *P* < 0.0001). **c-j**, Bone marrow cells collected from wild-type (WT) mice were incubated for 48 hours with fresh *C. concisus* culture (Live, CFU), heat-killed *C. concisus* culture (HK), or bacterial medium as a negative control (BHI). IL-1ß (**c**, F_(2,10)_ = 40.35, *P* < 0.0001; **d**, F_(2,11)_ = 1331, *P* < 0.0001), TNFα (**e**, F_(2,11)_ = 26.70, *P* < 0.0001), IL-5 (**f**, F_(2,11)_ = 38.63, *P* < 0.0001), IL-17 (**g**, *H*_(3,21)_ = 11.69, *P* = 0.0085; **h**, F_(2,11)_ = 24.01, *P* < 0.0001), and IL-22 (**i**, F_(2,10)_ = 5.162, P = 0.0288; **j**, F_(2,11)_ = 58.85, *P* < 0.0001) were quantified in cell supernatant. **k-o**, Bone marrow cells collected from *Nos2*^−/*−*^ mice were incubated for 48 hours with fresh (Live) or heat-killed (HK) *C. concisus* culture. Again, IL-1ß (**k**, F_(2,12)_ = 258.0, *P* < 0.0001), TNFα (**l**, F_(2,12)_ = 17.96, *P* = 0.0002), IL-22 (**m**, F_(2,12)_ = 117.1, *P* < 0.0001), IL-5 (**n**, F_(2,12)_ = 4.993, *P* = 0.0264) and IL-17 (**o**, F_(2,12)_ = 5.054, *P* = 0.0256) were quantified from cell culture supernatants. **p**, p38 MAPK activation was assessed in bone marrow cell lysates after 48 hours incubation bacterial medium (BHI), fresh *C. concisus* culture (Live), or heat-killed (HK) *C. concisus* culture (multiple unpaired two-tailed t-tests with two-stage step-up FDR correction, *n* = 5 biological replicates/group, except for the WT control, where *n* = 4 and stimulation of *Nos2*^−/*−*^ cells with heat-killed *C. concisus*, where *n* = 2; the latter was excluded from statistical comparison). For **a**-**f** and **h**-**o**, *P* values shown are the result of one-way ANOVA followed with Tukey’s multiple comparisons; **g**, Kruskal-Wallis test with Dunn’s multiple comparisons. For **a, d-f, h** and **j**, *n* = 5 replicates per group except for the BHI negative control where *n* = 4. For **b** and **g**, *n* = 5 replicates per group except for the addition of 4 × 10^5^ CFU *C. concisus*, where *n* = 6. For **c** and **i**, *n* = 5 biological replicates per group except for the BHI negative control, where *n* = 3. For **k-o**, n = 5 biological replicates per group. For all panels, bars denote mean ± s.d., Brain-Heart Infusion broth (BHI) was used as a negative control; CFU, colony-forming units of *C. concisus*.

To determine the ‘type’ of immune response elicited by *C. concisus* and whether the host’s capacity to produce nitric oxide plays a role, we collected bone marrow, which harbours a large population of diverse immune cells that seed other body sites, from WT and *Nos2*^−/*−*^ mice and stimulated these cells for 48 h with live or heat-killed *C. concisus*. Inducible nitric oxide synthase (iNOS) levels were significantly higher after co-culture of wild-type bone marrow cells with live and heat-killed *C. concisus* compared with bacterial medium alone (Extended Data Fig. 5a,b and Supplementary Table 6b). In alignment with our bulk RNA-seq data (Fig. 3f), IL-1β secretion was higher in response to heat-killed *C. concisus* and, in a dose-dependent manner, to co-culture with live *C. concisus* compared with medium alone (Extended Data Fig. 5c,d and Supplementary Table 6b). Production of TNF (canonical type 1 cytokine), IL-5 (type 2), IL-17 (main cytokine produced by Th17 cells) and IL-22 (type 3 and produced by Th17 cells) was significantly higher after co-culture with live *C. concisus* compared with medium alone (Extended Data Fig. 5e–j and Supplementary Table 6b). In contrast, incubation with heat-killed *C. concisus* only elicited a significant increase in IL-5 (Extended Data Fig. 5f and Supplementary Table 6b). Secretion of all five cytokines was significantly lower from cells obtained from *Nos2*^−/*−*^ compared with WT mice after co-culture with live *C. concisus*. (Fig. 4e–i and Supplementary Table 6b). In *Nos2*^−/*−*^ cells, levels of IL-1β, TNF and IL-22 remained significantly higher after incubation with live *C. concisus* compared with medium control (Extended Data Fig. 5k–m and Supplementary Table 6b). However, genetic ablation of iNOS ameliorated the IL-5 and IL-17 responses: levels in cell supernatants were not significantly higher in response to live *C. concisus* compared with medium control (Extended Data Fig. 5n,o and Supplementary Table 6b). Genes involved in MAPK signalling were more highly expressed in the colon of animals gavaged with *C. concisus* (Fig. 4f); p38 MAPK signalling was reduced in *Nos2*^−/*−*^ animals compared to WT, both after exposure to live *C. concisus* and at baseline (Extended Data Fig. 5p and Supplementary Table 6b). Together, these data suggest that *C. concisus*-induced cytokine signalling depends on iNOS, products of which the organism may exploit to gain a competitive advantage in the gut environment.

## Discussion

We describe a gnotobiotic mouse model of intergenerational transmission of members of the SI microbiota cultured from children with EED which phenocopied several aspects of human disease. In the absence of samples from healthy children living in the same environment as those in our clinical study, we generated two bacterial consortia from duodenal aspirates collected from Bangladeshi children with EED: one encompassing all isolates recovered from the aspirates and induced intestinal and systemic inflammation; the other a subset comprising one representative strain of each species present in the full collection. This ‘reverse translation’ approach provided an opportunity to perform analyses not possible in children—for example, immune profiling in the gut and extraintestinal tissues, snRNA-seq analysis in different regions of the SI and experimental tests of causality for disease-associated organisms.

We dissected structure–activity relationships in the human donor SI bacterial consortia by combining results from our dam-to-pup intergenerational transmission model with shorter duration co-housing experiments and isolate ‘add-in’ experiments, identifying *C. concisus* as a key driver in eliciting inflammation. These single-isolate manipulations and a mono-colonization experiment illustrated how inflammation induced by *C. concisus* occurred in a dose- and context-dependent manner: colonic inflammation was enhanced in the presence of *Actinomyces*, which themselves were not sufficient to elicit an immunoinflammatory response.

Experiments using heat-killed bacterial culture demonstrated that components and/or products of this pathology-inducing Bangladeshi *C. concisus* isolate induced immune signalling ex vivo. Our in vivo and in vitro studies suggested a role for bacterial nitrate and formate metabolism in pathogenesis of this organism. The mechanisms by which host nitric oxide generation and utilization of host-derived substrates by pathogenic bacteria contribute to decompartmentalization of the oral microbiota and enteropathy in humans warrant further investigation; the results could have therapeutic implications (Supplementary Discussion). Future studies should include microbial communities from distal regions of the intestines of individuals with EED to characterize how biogeographical features of the microbiota relate to the pathophysiology of this enteropathy.

Endoscopy studies performed as part of the BEED study established that EED is widespread in children and undernourished mothers^15,30^, supporting the notion that EED is an intergenerational health problem, at least in some low- and middle-income countries. Establishing a clinical infrastructure in these areas that allows concomitant sampling of the oral, SI and faecal microbiota of undernourished mothers with EED as well as healthy mothers without EED, with subsequent creation of preclinical models of the type described in this report, should provide an opportunity to obtain a mechanistic understanding of the interplay between the expressed functions of microbial community members, maternal gut health and nutritional status, intestinal adaptations to pregnancy, plus development before and following birth. An anticipated outcome of such an effort would be identification of candidate therapeutic targets in the microbiota that may be manipulated with dietary or more directed interventions.

## Methods

### Mouse experiments

All experiments involving mice were performed using protocols approved by Washington University Animal Studies Committee (IACUC 23-0271) and Institutional Biological and Chemical Safety (15436). C57Bl/6J mice (The Jackson Laboratory) were housed in plastic flexible film gnotobiotic isolators (Class Biologically Clean) at 23 °C under a strict 12-h light cycle (lights on at 6:00). Autoclaved paper ‘shepherd shacks’ were kept in each cage to facilitate natural nesting behaviours and for environmental enrichment.

#### Source of cSI-I and cSI-N bacterial consortia

The methods used for culturing bacterial strains from duodenal aspirates that were obtained from children enrolled in the BEED study are described in a previous publication^5^. Strains were stored at −80 °C in PBS containing 15% glycerol (v/v). For isolates originally isolated under anaerobic conditions, a 20-μl aliquot of each stock was used to inoculate a well in a 1-ml deep-well plate (Thermo Scientific) containing 600 µl LYBHI broth (brain-heart infusion [BHI] broth supplemented with 0.05% L-cysteine HCl [w/v] and 0.5% yeast extract [w/v]) followed by incubation under anaerobic conditions (atmosphere 75% N_2_, 20% CO_2_, 5% H_2_). For isolates originally isolated under microaerophilic conditions (85% N_2_, 10% CO_2_, 5% O_2_), a 20-µl aliquot of each stock was inoculated into 600 µl BHI broth. After incubation for 48 h at 37 °C, a 20-μl aliquot of each anaerobic or microaerophilic culture was added to 600 μl of fresh medium which was incubated for an additional 24 h at the same temperature under the same atmospheric conditions. Equal volumes of each isolate subculture were then pooled and a solution of PBS containing 30% glycerol (v/v) was added, resulting in a final 15% glycerol pooled stock (v/v). Pooling was performed in the Coy chamber. The 15% glycerol pooled stock was sealed in multiple 1.8-ml crimp glass vials (Wheaton) and the vials were stored at −80 °C before gavage into mice.

#### Diets

The ‘adult Mirpur’ diet was designed on the basis of 24-h dietary recall surveys and food frequency questionnaires taken from adults enrolled in the BEED study living in an urban slum located in the Mirpur district of Dhaka City, Bangladesh^31^. A pelleted, sterile version of this diet was manufactured by Dyets. The quantity of each ingredient used to prepare the diet is provided in Supplementary Table 1a. Rice (parboiled, long grain) and red lentils (masoor dal) were each cooked separately with an equal weight of water at 100 °C in a steam-jacketed kettle until the grains were cooked but still firm and then set aside. Tilapia fillets (frozen) were steamed separately at 100 °C in a steam-jacketed kettle with a small amount of water until tender (15–20 min). Sweet pumpkin (calabaza variety) was chopped, boiled in the steam-jacketed kettle until soft and then strained. Fresh market white potatoes, Daikon radish (moola), spinach, okra and yellow onions were washed, finely chopped and cooked together in the steam kettle without added water at 70 °C until soft. After cooling, all cooked ingredients were combined and mixed with wheat flour (atta), soybean oil, salt, turmeric, garlic and coriander powder. The resulting diet was mixed extensively using a planetary mixer, spread on trays, dried overnight at 30 °C and pelleted by extrusion (½ inch diameter; California Pellet Mill, CL5). Dried pellets were aliquoted into ~250 g portions and placed in a paper bag with an inner wax lining, which was then placed in a plastic bag. Bags were subsequently vacuum sealed and their contents sterilized by gamma irradiation (30–50 kGy, Sterigenics).

The composition of the ‘Mirpur-18’ diet was based on Bangladeshi complementary feeding practices for 18-month-old children living in Mirpur, as defined by quantitative 24-h dietary recall surveys conducted in the MAL-ED study^32^. This diet was manufactured according to a previously described protocol^7^.

The sterility of irradiated diets was confirmed by culture in (1) BHI broth, (2) nutrient broth and (3) Sabouraud-dextran broth (all from Difco) for 1 week at 37 °C under aerobic conditions, and in reduced tryptic soy broth (Difco) supplemented with 0.05% L-cysteine HCl under anaerobic conditions. All diets were stored at −20 °C before use. Nutritional analysis of each irradiated diet was conducted by Nestlé Purina Analytical Laboratories (Supplementary Table 1a).

#### Husbandry for intergenerational transmission experiments

Germ-free female C57Bl/6J mice (6–8-week-old) were given ad libitum access to an autoclaved breeder chow (LabDiet 5021, Purina Mills) until 3 days before colonization, at which time they were switched to the adult Mirpur diet for the remainder of the experiment. Mice received 200 µl of the stock solutions of the cSI-I or cSI-N consortium via an oral gavage needle. Another group of mice received an oral gavage of clarified caecal contents pooled from conventionally raised C57Bl/6J animals maintained on the standard chow (200 μl per recipient animal).

One week after initial gavage, trio matings were performed (two colonized females with one germ-free C57Bl/6J male). Females were maintained on the adult Mirpur diet. Pups born to these mothers remained with their dams in the same cage until weaning, at which time pups from the same litter were transferred to new cages and fed the Mirpur-18 diet. For dams and their pups, bedding was replaced every 7 days and diets were provided ad libitum. Following weaning of pups on postnatal day 21 (P21), trio matings were performed again using previously pregnant mice. Offspring were euthanized on P37 (first pregnancy) or P14 (subsequent pregnancies) without previous fasting. All biospecimens were flash frozen in liquid nitrogen and stored at −80 °C until analyses were performed.

For additional matings to assess fetal weights, adult germ-free female C57Bl/6J mice were switched to the adult Mirpur diet 3 days before gavage with 200 µl of the cSI-I or cSI-N consortium, as described above. Two weeks after gavage, female mice were mated in trios and pregnant dams were euthanized at embryonic day (E) 11.5 or E17.5.

#### Husbandry for co-housing experiments

Germ-free 4–5-week-old male C57Bl/6J mice were switched from a standard chow diet (Diet 2018S, Envigo) to the Mirpur-18 diet 3 days before colonization. Each mouse received 200 µl of the cSI-I or cSI-N bacterial consortium via a single oral gavage. Nine days later, mice were either maintained in their cage and isolator (non-co-housed control groups; *n* = 6–8 per group per experiment) or were transferred to a new cage in a new isolator to be co-housed with an equal number of mice transferred from an isolator containing the other experimental group (*n* = 6–8 per group per experiment). Bedding was replaced every 7 days for all groups of animals; the Mirpur-18 diet was provided ad libitum. All animals were weighed three times a week and euthanized without previous fasting on experimental day 18. All biospecimens were flash frozen in liquid nitrogen and stored at −80 °C before use.

#### Husbandry for tests of candidate mediators of EED (‘add-in’ experiments)

The design of this experiment was analogous to that of the co-housing experiment. Germ-free 4–5-week-old male C57Bl/6J mice were switched from a standard chow diet (Diet 2018S, Envigo) to the Mirpur-18 diet 3 days before initial colonization. Each mouse received 200 µl of the cSI-N consortium via a single oral gavage; all mice were maintained in a single gnotobiotic isolator. Nine days later, mice were distributed to new isolators and subsequently gavaged with 200 µl of either 15% PBS/glycerol alone (sham), the cSI-I bacterial consortium, or the cultured isolates that matched the *A. naeslundii*, *A*. *odontolyticus* and *C. concisus* MAGs. Mice received subsequent 200-µl gavages on days 10 and 11. Another reference control group of mice in their own isolator were initially gavaged with 200 µl of the cSI-I consortium, followed by gavage with medium alone on days 9 through 11. Bedding was replaced every 7 days and the Mirpur-18 diet was provided ad libitum. All animals were weighed three times a week and euthanized on experimental day 18 (first round of experiments) or 35 (follow-up more-prolonged exposure experiment).

#### Husbandry for mono-colonization experiment

Adult female germ-free C57Bl/6J mice were fed the Mirpur-18 diet ad libitum starting at 3 days before gavage. One group of animals was orally gavaged with 200 µl of a *C. concisus* monoculture grown in Bolton broth supplemented with 10% FBS (corresponding to 8.7 × 10^6^ colony-forming units (c.f.u.s)) on experimental days 0, 3, 5, 7 and 14. Another group received 200 µl of an equivalent number of cultured cells that had been heat killed (100 °C for 120 min), at the same time points as those used for administration of live cells. Live culture and heat-killed gavage mixes were prepared in 15% (v/v) glycerol. A third group of mice was maintained germ free. All animals were euthanized on experimental day 18.

#### Division of the intestine

At the time of euthanasia, the small intestine was removed and divided into thirds. Each third was then subdivided into two equal length subsegments (‘proximal’ and ‘distal’). The proximal subsegment was used for either histomorphometric analysis or flow cytometry. Intestinal contents were removed from the distal subsegment by gentle extrusion. The distal subsegment was then cut into three 1.5-cm-long pieces (labelled #1–3 on the basis of their proximal-to-distal location), flash frozen in liquid nitrogen and stored at −80 °C before use. These frozen pieces were used for bulk tissue RNA-seq (piece #1), snRNA-seq (piece #2) or ELISA- and Luminex-based protein quantification (piece #3). From the proximal end of the colon, three 1.5-cm-long pieces (labelled #1–3 on the basis of their proximal-to-distal location) were flash frozen for the same type of assays applied to small intestinal samples.

#### Histomorphometric and immunohistochemical analyses of intestinal tissue

Proximal subsegments of the duodenum and ileum (see *‘*Division of the intestine’ above) were pinned and fixed in 10% neutral buffered formalin for 24 h at 4 °C, followed by a 70% ethanol wash. These segments were oriented in parallel lines in agar and then collectively embedded in paraffin. Five-μm-thick sections were prepared and stained with haematoxylin and eosin and imaged using a Zeiss Axioscan 7 Slide Scanner. Ten well-oriented crypt–villus units were selected from each intestinal segment and villus height and crypt depth was quantified using QuPath (v.0.2.3)^33^. Measurements were performed for 4–6 mice per group, with the investigator blinded with respect to experimental group.

Sections (5-μm-thick) were prepared from formalin-fixed paraffin-embedded duodenal and ileal segments. Slides were deparaffinized and heat-induced antigen retrieval was performed using Trilogy (Millipore Sigma). Slides were blocked in PBS containing 2% BSA, 5% donkey serum and 0.1% Triton-X (antibody buffer) for 30 min at room temperature and treated with primary antibodies (Ki67 anti-rabbit, 1:300, Abcam AB16667; E-cadherin anti-mouse, 1:250, DB Sciences, 610181) for 90 min at room temperature. Following three cycles of washing in PBS, slides were incubated with secondary antibodies (Alexa Fluor donkey anti-rabbit 488, 1:300, Invitrogen, A32790; Alexa Fluor donkey anti-mouse, 1:300, Invitrogen, A32731) for 30 min at room temperature, followed by a wash in PBS, incubation with DAPI (1:1,000 dilution, Thermo Scientific, 62248) for 5 min and a final wash in PBS. Slides were mounted with ProLong Gold Antifade Mountant (Invitrogen, P36930) and imaged with a Zeiss Axioscan 7 Brightfield/Fluorescence Slide Scanner. Data were analysed using QuPath (v.0.2.3)^33^; as with the histomorphometric analysis, the investigator was blinded with respect to treatment group.

#### Protein assays of serum and intestinal tissue

Total intestinal protein was extracted by homogenizing a 1.5-cm-long piece of the duodenum, ileum or colon (piece #3, see ‘Division of the intestine’ above) in 600 μl of a solution of ice-cold T-PER Buffer (Thermo Scientific) with Complete Ultra protease inhibitor (Roche) using Lysis Matrix F beads (MP Bio). The homogenate was centrifuged at 13,000 × *g* for 5 min at 4 °C. Total protein concentration in the resulting supernatants was quantified using the Micro BCA Protein Assay kit (Thermo Scientific). Protein concentrations were normalized to 1 mg ml^−1^ in PBS with Complete Ultra protease inhibitor (Roche). Intestinal and/or serum levels of LCN2, IGF-1, CHI3L1 and S100A9 were quantified using Mouse Lipocalin-2/NGAL DuoSet ELISA, Mouse/Rat IGF-I/IGF-1 DuoSet ELISA, Mouse Chitinase 3-like 1 DuoSet ELISA and Mouse S100A9 DuoSet ELISA (R&D Systems), respectively, following manufacturer instructions. Intestinal and/or serum levels of several other proteins were measured using a custom Mouse Pre-Mixed Multi-Analyte kit (R&D Systems; CHI3L1, S100A9, MMP8, CXCL1, IL-17A, IL-17E) and the MILLIPLEX MAP Mouse Bone Magnetic Bead Panel (Millipore Sigma; DKK-1, FGF-23, OPG). Assays employing the latter two kits were conducted on a FlexMap3D instrument (Luminex).

### Flow cytometry of immune cell populations

#### Intergenerational transmission experiments

Subsegments of duodenum, ileum and/or colon (see ‘Division of the intestine’ above) were digested, and myeloid and lymphoid cells were collected according to methods previously described^5^. Briefly, each subsegment was immediately flushed with cold PBS after dissection to remove luminal contents. Each subsegment was then opened lengthwise and gently agitated for 20 min at room temperature in Hanks Balanced Salt Solution (HBSS) supplemented with 15 mM HEPES, 10% bovine calf serum (BCS) and 5 mM EDTA. Each sample was vortexed and the suspended cells were collected; the remaining tissue fragments were subjected to a second round of gentle agitation and vortexing. The tissue remaining after the second collection was rinsed with cold 1× HBSS before digestion with Collagenase IV (Sigma) in complete RPMI-1640 medium for 40 min at 37 °C with gentle agitation. Digests were filtered through a 100-μm mesh strainer (Falcon) and subjected to density gradient centrifugation using 40% and 70% Percol solutions (GE Healthcare).

To dissect meninges, skin and muscle overlying the skull as well as the mandibles and bone rostral to maxillae were removed. The remaining skull was placed in Iscove’s modified Dulbecco’s medium (IMDM, Sigma Aldrich). The meninges were removed from the skull cap using fine forceps and visualized under a light microscope. Meninges were digested for 20 min at 37 °C with 1.4 U ml^−1^ of Collagenase VIII (Sigma Aldrich) and 35 U ml^−1^ of DNAse I (Sigma Aldrich) in IMDM. Following digestion, the tissue was gently pressed through a 70-μm mesh cell strainer (Falcon). The flow-through material was centrifuged at 450 × *g* at 4 °C for 4 min. Spleens were processed in a manner similar to that used for the meninges except that we performed an additional lysis step with ammonium-chloride-potassium (ACK) lysis buffer (Quality Biological) before staining.

Cells collected from each sample type were resuspended in ice-cold FACS buffer (2 mM EDTA, 25 mM HEPES, 1% BSA in 1× PBS) and stained for extracellular markers at 1:300 dilution. Dead cells were excluded using Zombie NIR fixable Viability kit. Cells were analysed on a Cytek Aurora flow cytometer (Cytek Biosciences) and the results assessed using FlowJo (v.10.8.1). The fluorophore-labelled monoclonal antibodies used for flow cytometry are listed in Supplementary Table 7 and gating strategies are presented in Supplementary Fig. 13.

#### Co-housing and add-in experiments

Mice were injected retro-orbitally (under isoflurane anaesthesia 2 min before euthanasia) with 2 μg of phycoerythrin (PE)-conjugated anti-CD45 antibody (Biolegend, clone 30-F11) to label intravascular leucocytes. Blood was collected from the retro-orbital sinus following euthanasia, centrifuged and lysed using ACK lysis buffer (Quality Biological) for 1 min at room temperature, followed by addition of 2 ml 1× PBS. This lysis step was repeated two more times. The resulting material was subjected to centrifugation at 420 × *g* for 4 min. Cell pellets were resuspended in FACS buffer (PBS with 2% bovine serum albumin) and stained for extracellular markers at 1:300 dilution.

Immune cells from intestines, spleen and meninges were collected as described above for the intergenerational experiment. Dead cells were excluded using Zombie NIR fixable Viability kit. Cells were analysed on a Cytek Aurora (Cytek Biosciences) and the data assessed using FlowJo (v.10.8.1). The fluorophore-labelled monoclonal antibodies used for flow cytometry are listed in Supplementary Table 7.

### Metagenome-assembled genomes

#### Identification of MAGs

DNA was extracted from flash-frozen caecal contents obtained from P37 cSI-I and cSI-N mice and their dams in the intergenerational experiment, as well as from non-co-housed and co-housed mice in the co-housing experiment. Caecal contents were subjected to bead beating and phenol–chloroform extraction to obtain crude genomic DNA, which was subsequently purified (QIAquick 96 PCR Purification kit) and quantified (Qubit). The final DNA fragment size distribution was determined using an Agilent Technologies 4200 TapeStation.

Fragmented genomic DNA (400–1,000 ng) was prepared for long-read sequencing using a SMRTbell Express Template Prep Kit 2.0 (Pacific Biosciences) adapted to a deep 96-well plate (Fisher Scientific) format. All DNA handling and transfer steps were performed with wide-bore genomic DNA pipette tips (ART). Barcoded adapters were ligated to A-tailed fragments (overnight incubation at 20 °C), and damaged or partial SMRTbell templates were subsequently removed (SMRTbell Enzyme Cleanup kit). High molecular weight templates were purified (the volume of added undiluted AMPure beads used was 0.45 times the volume of the DNA solution). A second round of size selection was performed by diluting AMPure beads to a final concentration of 40% (v/v) with SMRTbell elution buffer, after which the resulting mixture was added at 2.2 times the volume of the pooled libraries. DNA was eluted from the AMPure beads with 12 µl of SMRTbell elution buffer. Pooled libraries were quantified (Qubit) and their size distribution was assessed using a TapeStation (Agilent). Libraries were then sequenced to a depth of 1.85 ± 1.5 × 10^9^ reads per sample (mean ± s.d.) using a Sequel II System instrument (Sequel Binding Kit 3.0 and Sequencing Primer v.4, Pacific Biosystems). The resulting reads were demultiplexed and Q20 circular consensus sequencing (CCS) reads were generated (Cromwell workflow conFig.d in SMRT Link). CCS reads were assembled into contigs using metaFlye (v.2.8.1)^34^; with hifi-error set to 0.003 and other options set to default. Contig quality was evaluated using checkm (v.1.0.7)^35^. Any contig demonstrating checkm ‘completeness’ ≥85% and ‘contamination’ ≤5% was nominated as a ‘high-quality’ MAG. Remaining contigs were subjected to further MAG assembly efforts, first by calculating coverage using CoverM (v.0.6.1, https://github.com/wwood/CoverM) and then MAG reconstruction using MaxBin (v.2.2.7)^36^. MAGs were cleaned using MAGpurify (v.2.1.2)^37^ and evaluated for quality using checkm. In an iterative process, candidate MAGs demonstrating checkm completeness ≥85% and contamination ≤5% were again nominated as a MAG. High-quality MAGs from both primary and subsequent MAG assembly efforts were dereplicated using dRep (v.2.3.2)^38^. Final MAG summary statistics were collected with checkm and quast (v.4.5)^39^ (Supplementary Table 1c).

#### Taxonomic classification of MAGs

Taxonomic assignments were initially made by employing the Genome Taxonomy Database Toolkit (GTDB-Tk, v.1.5.1)^40^ and corresponding database (release 95). Phylogenetic trees of MAGs and closely related reference genomes were generated using the Phylogenetic Tree Building service available from the Bacterial and Viral Bioinformatics Resource Center (BV-BRC^41^). This service utilizes the Codon Tree method and universal protein families as homology group and analyses alignments of these proteins identified in each genome using the programme RAxML. The genome trees obtained were visualized via iTOL^42^.

#### Determination of the absolute abundances of MAGs and isolate genomes

The absolute abundances of MAGs were determined using previously described methods with minor modifications^43,44^. In brief, ‘spike-in’ bacterial strains with genomes easily differentiated from those of gut bacteria were added to each weighed frozen sample of intestinal contents or faeces before DNA isolation and preparation of barcoded libraries^27^. For intergenerational transmission and co-housing experiments, *Alicyclobacillus acidiphilus* spike-in was added (2.22 × 10^8^ cells per ml suspension; DSM 14558; GenBank assembly accession GCA_001544355.1)^44^. For isolate add-in experiments, a commercial 1:1 mix of *Imtechella halotolerans* and *Allobacillus halotolerans* was added (2 × 10^7^ cells each per 20 µl suspension; Zymo product numbers LMG 26483 and LMG 24826, respectively).

Intestinal contents and faecal samples were subjected to bead beating and phenol–chloroform extraction to obtain crude genomic DNA which was subsequently purified (QIAquick 96 PCR Purification kit). Shotgun sequencing libraries were constructed using Nextera XT DNA Library Prep kit and sequenced on an Illumina NovaSeq 6000 instrument (150-nt paired-end reads; 6.17 × 10^6^ ± 3.75 × 10^7^ reads per ample (mean ± s.d.) from the intergenerational transmission experiment, 3.10 × 10^6^ ± 7.94 × 10^6^ reads per sample from the co-housing experiments, and 3.03 × 10^7^ ± 1.30 × 10^8^ reads per sample from the isolate add-in experiment). Sample metadata are listed in Supplementary Table 8. Due to increased host reads in small intestinal samples, libraries were balanced on the basis of a host:microbe read ratio determined by preliminary shallow sequencing.

Reads were demultiplexed (bcl2fastq), trimmed (trimgalore, v.0.6.1; https://github.com/FelixKrueger/TrimGalore) and filtered to exclude host reads using bowtie2 (v.2.3.5)^45^. MAG abundances were determined by assigning reads to each MAG, followed by a normalization for genome uniqueness in the context of a given community^27^. The resulting counts table was imported into R (v.4.0.4). We calculated the absolute abundance of a given MAG *i* in sample *j* using the following equation:

1 $$\begin{array}{ll}{\mathrm{strain}}_{i,j}=\left(\displaystyle\frac{{\mathrm{counts}}_{i,j}\times {\mathrm{spikein}1\,\mathrm{cells}\,\mathrm{added}}_{j}}{{\mathrm{spikein}1\,\mathrm{counts}}_{j}\times {\mathrm{sample}\,\mathrm{weight}}_{j}}\right.\\\left.\qquad\quad\,\,\,\,+\displaystyle\frac{{\mathrm{counts}}_{i,j}\times {\mathrm{spikein}2\,\mathrm{cells}\,\mathrm{added}}_{j}}{{\mathrm{spikein}2\,\mathrm{counts}}_{j}\times {\mathrm{sample}\,\mathrm{weight}}_{j}}\right)\,\times \,0.5\end{array}$$

The statistical significance of observed differences in the abundances of a given MAG across different groups was tested using a non-parametric Wilcoxon rank-sum test with false discovery rate (FDR) corrections on calculated log_10_ absolute abundances. Final MAG abundances for all experiments are included in Supplementary Table 1d.

#### Identification and assembly of isolate genomes related to MAGs

Average nucleotide identity was calculated using dRep (v.2.3.2)^38^ for all genomes assembled by shotgun sequencing (paired-end 150 bp reads) of cultured isolates from the BEED study duodenal aspirates (Supplementary Table 1b)^7^. For cultured isolates displaying ANI ≥ 95% shared with MAGs of interest obtained from the intergenerational and co-housing experiments, monocultures of each isolate were grown in 10 ml broth overnight at 37 °C under anaerobic conditions (atmosphere; 75% N_2_, 20% CO_2_ and 5% H_2_) without shaking. Cells were recovered by centrifugation (5,000 × *g* for 10 min at 4 °C) and high molecular weight genomic DNA was purified (QIAquick 96 PCR Purification kit) and quantified (Qubit); the final fragment size distribution was determined using a TapeStation (Agilent). Fragmented genomic DNA (400–1,000 ng) was prepared and long-read sequencing was performed as described above. The resulting reads were demultiplexed and Q20 CCS reads were generated (Cromwell workflow conFig.d in SMRT Link). Genomes were assembled using Flye (v.2.9)^46^ with hifi-error set to 0.003 and other options set to default. Assembly quality was evaluated using checkm. Taxonomic assignments were initially made by employing the Genome Taxonomy Database Toolkit (GTDB-Tk, v.1.5.1)^40^ and corresponding database (release 95). Final assembly summary statistics were collected with checkM, quast and dRep (Supplementary Table 4a). All isolates were of high quality based on marker gene analysis, consisted of one or two contigs and shared >99.9999% ANI with their respective MAG (Supplementary Table 4a).

### Quantitative polymerase chain reaction (qPCR)

To determine the absolute abundance of *C. concisus* in mono-colonized animals and animals from the add-in experiment, we performed qPCR on DNA extracted from duodenal, jejunal, ileal, caecal and colonic contents. DNA was extracted using the QIAamp Powerfecal Pro DNA kit and amplified in quadruplicate 10-µl reactions that contained 5 µl i*Taq* Universal SYBR Green Supermix (Bio-Rad), 0.4 µl of a 10 µM stock solution of primers targeting the *C. concisus cpn60* gene^47^ (JH0023: GGCTCAAAAGAGATCGCTCA; JH0024: CCCTCAACAACGCTTAGCTC) and 1.36 μl of a 3 ng µl^−1^ solution of DNA isolated from intestinal contents or 2.5 µl of a 1 ng µl^−1^ reference control solution of genomic DNA isolated from a monoculture of *C. concisus*. Amplification was performed using a QuantStudio 6 Flex machine and the following cycling conditions: 95 °C for 3 min, followed by 40 cycles of 15 s at 95 °C, 15 s at 64.6 °C, 15 s at 72 °C and a final melt at 95 °C for 1 min. *C. concisus* genome equivalents in DNA extracted from mouse intestinal samples was calculated using the standard curve of *C*_t_ values generated from a purified preparation of *C. concisus* genomic DNA.

### Bulk RNA-seq

#### Intestinal tissue

RNA was extracted from 1.5 cm flash-frozen segments of the duodenum, ileum and/or colon (piece #1, see ‘Division of the intestine’ above) using the Qiagen RNeasy 96 kit. Total RNA was quantified (Qubit) and quality was assessed using a TapeStation (Agilent). Complementary (c)DNA libraries were generated using the Illumina Total RNA Prep with Ribo-Zero kit (Illumina). Barcoded libraries were sequenced on an Illumina NovaSeq 6000 instrument (150-nt paired-end reads to a depth of 3.23 × 10^7^ ± 3.54 × 10^6^ reads per sample (mean ± s.d.) from the intergenerational experiment and 7.13 ± 3.08 × 10^7^ reads per sample from the co-housing experiment). Sample metadata are provided in Supplementary Table 8.

#### Data analysis

Read quality was verified with FastQC (v.0.11.7; http://www.bioinformatics.babraham.ac.uk/projects/fastqc/). Reads were then trimmed to remove adapters and low-quality read segments using trimgalore (v.0.6.1). Trimmed reads were pseudoaligned to a kallisto (v.0.46.2)^48^ index built from the Gencode v.25 *Mus musculus* reference genome. Transcript counts were aggregated to gene counts. Kallisto ‘estimated counts’ values and transcripts per million (TPM) values were used to generate a gene-level expression matrix in R using tximport (v.1.22.0)^49^ and biomaRt (v.2.50.3)^50^. The ‘lengthscaledTPM’ function in tximport was used to adjust estimated counts by gene length and abundance. This resulting count matrix was imported into limma-voom (v.3.50.3)^51,52^ to compare log_2_(counts per million) gene expression between groups. Gene set enrichment analysis (GSEA) was performed using clusterProfiler (v.4.2.2)^53^ and the Gene Ontology (GO) pathway database, with FDR correction.

### Epithelial cell death induction assay

HCT116 cells were cultured at 37 °C in DMEM medium supplemented with 10% FBS at 4 × 10^5^ cells per ml. Cells were washed with 1× PBS and selected controls were pre-treated with DMSO (0.1%) or 30 μM caspase inhibitor (QVD, Quinoline-Val-Asp-Difluorophenoxymethylketone) for 1 h before inducing apoptosis with administration of 1 μM staurosporine (STS, Abcam) for 24 h.

CT26:FADD cells were seeded at 2 × 10^6^ cells per 10-cm culture dish. The following day, cultures were incubated with 1 μg ml^−1^ doxycycline for 16 h to induce expression of the Fas-associated death domain (FADD) construct. Doxycycline was removed by washing with 1× PBS before 1 h pre-treatment with 20 μM z-VAD-FMK (z-VAD, pan-caspase inhibitor, MedChem Express) or DMSO (0.1%). Cell death was induced by adding 10 nM B/B homodimerizer for 5 h, after which time supernatants were collected and centrifuged at 350 × *g* for 5 min to remove cellular debris. The resulting supernatants were filtered using a 0.2-μm syringe filter (SFCA, Corning) and then frozen at −20 °C for later use in *C. concisus* growth assays.

#### *C. concisus* growth in conditioned medium from intestinal epithelial cells

*C. concisus* isolate Bg048 was routinely cultured in Bolton broth supplemented with 10% FBS (autoclaved and subsequently filter sterilized) and on BHI–agar plates supplemented with 7.5–10% (v/v) horse or sheep blood. For growth in spent medium collected from colonic epithelial cell lines, 25 µl of a 24-h culture of *C. concisus* in Bolton broth with 10% FBS was subcultured into a 1:1 (v/v) mixture of Bolton broth with 10% FBS and intestinal epithelial cell culture spent medium or blank cell culture medium as a control (DMEM). After a 6–24-h incubation at 37 °C, a 30-µl aliquot of the resulting culture was removed for serial dilutions and the determination of c.f.u.s using blood agar plates incubated under microaerophilic conditions (5% O_2_, 10% CO_2_, 85% N_2_) or anaerobic conditions (5% H_2_, 20% CO_2_, 75% N_2_). After 48 h of growth, *C. concisus* colonies were counted and growth relative to DMEM was evaluated.

### Microbial RNA-seq

#### *C. concisus* gene expression in the mouse gut

RNA was isolated from caecal contents collected from cSI-I and cSI-N sham-gavaged controls, and the ‘cSI-N plus isolate add-in’ mice described in Fig. 4 and Extended Data Fig. 4. cDNA libraries were generated from isolated RNA samples using the Total RNA Prep with Ribo-Zero Plus kit (Illumina). Barcoded libraries were sequenced on an Illumina NovaSeq 6000 instrument (150-nt paired-end reads to a depth of 1.93 × 10^8^ ± 7.94 × 10^7^ reads per sample (mean ± s.d.); *n* = 6 samples per treatment group). Sample metadata are listed in Supplementary Table 8. Raw reads were trimmed (trimgalore, v.0.6.1), filtered to exclude host reads (bowtie2, v.2.3.5), and mapped to MAGs (kallisto, v.0.46.2) and the *C. concisus* isolate genome (see ‘Identification and assembly of isolate genomes related to MAGs’ in Methods). The resulting kallisto pseudocount tables were imported to R (v.4.0.4). The geometric mean of absolute abundance (see ‘Determination of the absolute abundances of MAGs and isolate genomes’) was used to set sizeFactors for the DESeq2 object before differential expression with the DESeq2 Wald test.

#### *C. concisus* gene expression in vitro

*C. concisus* was grown anaerobically on solid BHI–agar supplemented with 10% sheep blood. After 48 h, single colonies were picked and inoculated into rich medium (Bolton broth supplemented with 10% FBS); after 24 h of anaerobic growth at 37 °C, an aliquot was diluted 1:32 into media used for assessing gene expression; these conditions included (1) Bolton broth containing 10% FBS ± 1% mucin, grown anaerobically and (2) Bolton broth containing 10% FBS mixed 1:1 (v/v) with conditioned medium from HTC116 colonic epithelial cells, grown under microaerophilic conditions. The colonic epithelial cell media conditions used were as follows; (1) conditioned medium from HTC116 cells grown in DMEM, (2) conditioned medium from HTC116 cells treated with L-NIO and (3) DMEM cell culture medium control. After 24 h, these broth cultures were transferred to 5 ml snap-top microcentrifuge tubes and centrifuged at 20,000 × *g* for 30 min at 10 °C to pellet *C. concisus* cells. Supernatant was removed and cell pellets were frozen at −20 °C. RNA was extracted with the Qiagen RNeasy UCP Micro kit. Briefly, 200 µl of acid-washed glass beads were added to the bacterial pellets on ice with 350 µl buffer RULT containing 2-mercaptoethanol. Bead beating was performed for 5 min; RNA was extracted according to manufacturer specifications, including on-column DNase treatment. Quality was assessed with RNA High Sensitivity ScreenTape using an Agilent Technologies 4200 TapeStation and quantified with the Qubit RNA High Sensitivity Assay kit (Invitrogen, Q32852). ERCC RNA Spike-In Mix (Invitrogen, 4456740) was added before library preparation, which was performed using the Total RNA Prep with Ribo-Zero Plus kit (Illumina). Barcoded libraries were sequenced (AVITI instrument, Element Biosciences; 150-nt paired-end reads; 1.03 × 10^7^ ± 7.4 × 10^6^ reads per sample (mean ± s.d.)). Demultiplexed reads were quality trimmed (trimgalore) and mapped to the *C. concisus* isolate genome Bg048 and ERCC transcripts (kallisto). Kallisto pseudocount tables were imported into R, and ERCC counts were extracted to create a separate DESeq2 object from which sizeFactors were calculated and centred. These sizeFactors were used to normalize the *C. concisus* DESeq2 object before differential expression testing (Wald test).

To facilitate comparison of gene expression between the in vivo and in vitro experimental conditions, reads from *C. concisus* housekeeping genes (*rpoA*, *gyrB*, *recA*, *dnaK* and *atpA*) were extracted; the geometric mean of these five housekeeping genes was calculated per sample and centred for the DESeq2 sizeFactors in a manner analogous to the ERCC spike-in control.

### *C. concisus* co-culture with mouse immune cells

Conventionally raised 8–12-week-old female wild-type C57Bl/6J (The Jackson Laboratory) or *Nos2*^−/*−*^ (B6.129P2-*Nos2*^*tm1Lau*^/J, Jackson) mice fed standard mouse diet (LabDiet, 5010) were euthanized. Femurs were collected and placed into RPMI-1640 supplemented with 2 mM L-glutamine and 10% FBS and kept on ice. Bone marrow was extracted from each femur by centrifugation and resuspended in 1 ml RPMI. Briefly, femurs were placed vertically into a 0.65-ml snap-top tube (Avant, 2924) with a puncture hole created by an 18-gauge needle. The tube was placed within a 1.5-ml microcentrifuge tube (Eppendorf) and samples were centrifuged at >10,000 × *g* for 15 s. Bone marrow cells were resuspended in RPMI and pooled between mice (*n* = 2 animals per genotype per experiment) and 200-µl aliquots were placed into wells of a 24-well tissue culture plate. Live *C. concisus* cultures were grown anaerobically in Bolton broth for 24 h or heat killed at 100 °C for 2.5 h; 400 µl was added to each well containing bone marrow from WT or iNOS^−/*−*^ mice. Cells were cultured under an atmosphere of 5% CO_2_ at 37 °C for 48 h. Tissue culture plates were centrifuged at 500 × *g* to pellet cells and collect cell supernatants, which were aliquoted into PCR plates and stored at −20 °C. Cell pellets were washed with 1 ml PBS, centrifuged at 1,000 × *g*, and stored at −20 °C until protein extraction and quantification.

#### Quantification of cytokines with ELISAs

Bone marrow cell supernatants were thawed and cytokines were assayed using 50 µl of undiluted cell supernatant following manufacturer instructions (IL-1B, Abcam, 197742; IL-17, Abcam, 199081; IL-22, Abcam, 223857; IL-5, Abcam, 204523; TNF, Abcam, 208348; MAPK signalling, Invitrogen, 85-86195-11). To quantify levels of iNOS in bone marrow cell pellets, protein was extracted using the cell lysis buffer provided in the iNOS ELISA kit (Abcam, 253219). The cell extract was diluted 1:3 to quantify protein concentration using the Micro BCA Protein Assay kit (Thermo Scientific, 23235). Of the cell extract, 50 µl was used to quantify iNOS following instructions provided with the ELISA kit; final iNOS concentration was normalized to total protein concentration.

### Ethics and inclusion

This work was performed as part of a long-standing collaboration investigating the role of gut microbiota in undernutrition that is covered by a memorandum of understanding between teams led by Tahmeed Ahmed (International Centre for Diarrhoeal Disease Research (icddr,b) Bangladesh) and Jeffrey Gordon (Washington University in St Louis, USA). Bacterial isolates used in this study were obtained from duodenal aspirates collected from the previously reported Bangladesh EED study, which was approved by the Ethical Review Committee (ERC) at the icddr,b (protocol no. PR-16007; ClinicalTrials.gov number NCT02812615). Written informed consent to participate and to undergo EGD if the child met the inclusion criteria was obtained from each child’s parent or guardian. Human biospecimens were transferred to Washington University under a Materials Transfer Agreement (MTA). Bacterial isolates cultured and used in this study are the property of icddr,b and are available under an MTA upon request to T.A. and J.I.G.

### Reporting summary

Further information on research design is available in the Nature Portfolio Reporting Summary linked to this article.

## Supplementary information

Supplementary Information Supplementary Results, Discussion, Methods, Figs. 1–13 and References.

Reporting Summary

Supplementary Table 1 **Diet and bacterial abundance information from gnotobiotic mouse experiments. a**, Diets used in gnotobiotic mouse studies. Ingredients in and nutritional analysis of each diet. **b**, Cultured isolates and taxonomic classification from BEED study duodenal aspirates. **c**, MAG assembly information and MAG taxonomic classification. (i) Assembly and taxonomic classification. (ii) Sample metadata. **d**, Abundances of MAGs from gnotobiotic animal experiments. (i) Diet oscillation experiment, log_10_-tpm (Supplementary Fig. 3). (ii) Intergenerational transmission experiment, absolute abundance: P37 animals (Figs. 1b and 2j, and Supplementary Fig. 2), P14 animals (Supplementary Fig. 2d) and adults (Supplementary Fig. 2a–c). Relative abundance: gavage mixes. (iii) co-housing experiment, absolute abundance (Fig. 2h,i). (iv) Isolate add-in experiments, absolute abundance (Fig. 3b and Extended Data Fig. 4a,b). Abundance values are log_10_ transformed. *P*_adj_ values determined using two-sided Wilcoxon rank-sum tests and FDR correction. **e**, *C. concisus* abundance in (i) mono-colonization and (ii) add-in experiments assessed by qPCR (Extended Data Fig. 4g).

Supplementary Table 2 **Phenotypic data from gnotobiotic mouse experiments. a**, Body weights from gnotobiotic animal experiments. (i) Preliminary test of consortia in recently weaned mice (Supplementary Fig. 1a); (ii) Intergenerational transmission experiment: P37 animals, P14 animals (Extended Data Fig. 2d) and conceptuses (Fig. 1c and Extended Data Fig. 2a–c) (Wilcoxon rank-sum tests). (iii) co-housing experiments (linear mixed-effects modelling). (iv) Gavage of pathology-associated isolates, ‘add-in’ experiments (Extended Data Fig. 4c,d) (repeated measures ANOVA). **b**, Protein levels in tissues from gnotobiotic animal experiments. (i) First test of consortia in recently weaned mice (Supplementary Fig. 1b,c,f). (ii) Diet oscillation experiment (Extended Data Fig. 2b). (iii) Intergenerational transmission experiment: P37 duodenum, ileum, colon and serum (Fig. 1f–h and Extended Data Fig. 3b–e); P14 duodenum, ileum, colon and serum (Extended Data Fig. 2f–l); adult duodenum, ileum, colon and serum. (iv) Co-housing experiments: duodenum, ileum, colon and serum (Fig. 2b,c). (v) Isolate add-in experiments: duodenum, colon and serum (Fig. 3c,e and Extended Data Fig. 4e,f). (vi) *C. concisus* mono-colonization experiment: duodenum, ileum, colon and serum. **c**, Histomorphometric analysis of small intestines of P37 animals from the intergenerational transmission experiment. (i) Duodenal (Fig. 1d,e) and (ii) ileal villus height, crypt depth, villus to crypt ratio (Extended Data Fig. 3a) and Ki67 quantification (Supplementary Fig. 6c,d). *P* values determined using Tukey’s post hoc tests. **d**, Micro-computed tomography of femurs of P37 animals from the intergenerational experiment. *P* values were determined using Wilcoxon rank-sum tests. **e**, Quantification of small intestinal acylcarnitines in P37 offspring (log_10_-µM) (Supplementary Fig. 4). **f**, Immune cell profiling of tissues from gnotobiotic animal experiments. (i) Intergenerational transmission experiment: P37 duodenum, ileum, colon, spleen and meninges (Fig. 1i,j and Extended Data Fig. 3f,g); duodenum, ileum, spleen and meninges of the dams. (ii) Co-housing experiment: bulk small intestinal tissue, spleen and meninges (Fig. 2d–g). *P* values determined using Wilcoxon rank-sum tests. **g**, Nitrate and nitrite quantification from isolate ‘add-in’ experiment (Fig. 4b and Supplementary Fig. 11a).

Supplementary Table 3 **Supplementary data for host RNA-sequencing. a**, Bulk tissue RNA-seq from gnotobiotic animal experiments. (i) Preliminary tests of consortia in recently weaned animals, significantly differentially expressed genes and results of GSEA between cSI-I and cSI-N-colonized mice 28 days after gavage in the duodenum, jejunum and ileum (Supplementary Fig. 1d,e). (ii) Intergenerational transmission experiment: gene set enrichment analysis (GSEA) of gene expression in the duodenum, ileum and colon of P37 animals. (iii) Co-housing experiment: differentially expressed genes in duodenal samples for: non-co-housed cSI-I controls versus non-co-housed cSI-N controls and co-housed versus non-co-housed cSI-N controls. There were no statistically significant differentially expressed genes between co-housed and cSI-I controls. GSEA of gene expression in the colon of non-co-housed cSI-I controls vs non-co-housed cSI-N controls and co-housed vs non-co-housed cSI-N controls (Fig. 3f). (iv) Add-in experiment: GSEA of gene expression in the colon. Pathways shown were enriched in all the following conditions: co-housed animals vs cSI-N non-co-housed controls, *C. concisus*+*Actinomyces*-gavaged animals vs cSI-N sham-gavaged controls and *C. concisus*-gavaged animals vs cSI-N sham-gavaged controls. Values provided (*P* and *q* values, enrichment scores, rank, leading edge, core enrichment) are for *C. concisus+Actinomyces*-gavaged vs cSI-N sham-gavaged animals (Fig. 3f). **b**, Proportions of cell types quantified by snRNA-seq in the duodenums (i) and ileums (ii) of P37 animals from the intergenerational transmission experiment (Supplementary Fig. 5a,b) (*n* = 3 mice per group; statistical significance determined using edgeR). **c**, snRNA-seq analysis of duodenums and ileums of P37 offspring from intergenerational transmission experiment. (i) Pseudo-bulk differential expression of enterocyte clusters in the duodenum and ileum of cSI-I compared to cSI-N animals (*n* = 3 mice per group; DESeq2 Wald test). (ii) GSEA of gene expression along the crypt–villus axis (Supplementary Fig. 5c). (iii) GSEA and annotations for genes involved in efferocytosis (Supplementary Fig. 5d). (iv) ‘Differential NicheNet’ results (Supplementary Fig. 6a,b). (v) GSEA of homologues in the mouse gut (P37 duodenum and ileum) to LAZ-associated proteins quantified in the duodenal mucosa of children in the BEED study (Supplementary Fig. 7a,b). **d**, GSEA results in the duodenum and colon for bulk RNA-seq datasets of animals from co-housing and ‘add-in’ experiments; all treatment groups presented were compared to their cSI-N counterparts (Fig. 3f).

Supplementary Table 4 **Comparative genomics analysis. a**, Isolate genome assembly information and taxonomic classification. (i) Quality assessment and taxonomic classification of assembled isolate genomes. (ii) Average nucleotide identity (ANI) of isolate genomes with their related MAGs. (iii) Species-specific virulence factors in MAGs, their respective isolate genomes and related reference genomes. **b**, In silico metabolic reconstructions of MAGs and isolate genomes. (i) mcSEED-based metabolic annotation for each MAG by gene. (ii) Annotations for pathology-associated MAGs and phylogenetically related organisms by gene. (iii) ‘Metabolic phenotype’ prediction key. **c**, *C. concisus* pan-genome analysis (Supplementary Fig. 8). (i) List of 120 isolate genomes included in pan-genomic analysis and their association with disease. Enrichment analyses performed on predicted genes (ii), COG20 functional annotations (iii) and COG Pathway annotations (iv). Enrichment results were included if functional groups were enriched in isolates from diseased conditions (Crohn’s disease, ulcerative colitis, gastroenteritis, periodontitis) including EED, but not in isolates from healthy controls.

Supplementary Table 5 **Supplementary data for microbial RNA-sequencing. a**, Significantly differentially expressed *C. concisus* genes in the gut compared to growth in culture (Supplementary Fig. 9a) (DESeq2 Wald test). (i,ii) *C. concisus* gene expression in the gut (single isolate ‘add-in’) compared to rich medium (Bolton broth ±1% mucin). (iii,iv) *C. concisus* gene expression when gavaged concurrently with the *Actinomyces* strains (*C. concisus*+*Actinomyces*) compared to gene expression in rich medium (Bolton broth ±1% mucin). **b**, *C. concisus* gene expression in the mouse gut in conditions where inflammation was produced [*C. concisus* (i), *C. concisus*+*Actinomyces* (ii), cSI-I (iii)] compared to cSI-N/mock-gavaged controls (Fig. 5c and Supplementary Fig. 9b). **c**, *C. concisus* gene expression in spent medium collected from HTC116 cells compared to medium alone (Supplementary Fig. 11d) (i). *C. concisus* gene expression in conditioned medium from HTC116 cells treated with the pan-nitric oxide inhibitor L-NIO compared to medium control (ii).

Supplementary Table 6 **Ex vivo** ***C. concisus*** **growth and immune signaling assays. a**, *C. concisus* growth in conditioned medium collected from a mouse colonic epithelial cell line (CT26) and a human colonic epithelial cell line (HTC116) (Fig. 4d, and Supplementary Figs. 10 and 11b,c). **b**, Quantification of iNOS within and cytokines secreted by wild-type (WT C57Bl/6J) and iNOS^−/*−*^ (*Nos2*^−/*−*^) bone marrow cells stimulated with live and heat-killed *C. concisus* (Fig. 4e–i and Extended Data Fig. 5). **c**, *C. concisus* growth in and depletion of formate; IL-1ß induction after formate pre-treatment (Supplementary Fig. 12b–d).

Supplementary Table 7 **Antibody panels for flow cytometry analyses**. (i) Antibodies used for flow cytometry of intestinal immune cells in intergenerational transmission and co-housing experiments (Fig. 1i,j, Extended Data Fig. 3f,g and Supplementary Fig. 13). (ii,iii) Antibodies used for flow cytometry of splenic and meningeal immune cells in intergenerational transmission (ii) and co-housing experiments (iii) (Fig. 2f,g and Supplementary Fig. 13).

Supplementary Table 8 **Metadata associated with sequencing datasets**. Sequencing metadata from gnotobiotic animal and bacterial culture experiments. **a**, Bulk RNA-seq collected from preliminary test of consortia (Supplementary Fig. 1d,e). **b**, Intergenerational transmission experiment: (i) short read shotgun DNA sequencing (Figs. 1b and 2j, and Supplementary Fig. 2), (ii) snRNA-seq (Supplementary Figs. 5, 6a,b and 7), (iii) bulk tissue RNA-seq (Fig. 3f) and (iv) microbial RNA-seq. **c**, Co-housing experiment: (i) short read shotgun DNA sequencing (Fig. 2h,i), (ii) bulk tissue RNA-seq (Fig. 3f) and (iii) microbial RNA-seq. **d**, Isolate add-in experiments: (i) short read shotgun DNA sequencing (Fig. 3b and Extended Data Fig. 4a,b), (ii) bulk tissue RNA-seq (Fig. 3f) and (iii) microbial RNA-seq (Supplementary Fig. 9b). **e**, *C. concisus* RNA-seq in culture medium and conditioned medium collected from HTC116 cells (Supplementary Figs. 9a, 11d and 12a).

## Data Availability

Datasets generated by (1) shotgun sequencing of DNA isolated from (a) the intestinal contents of gnotobiotic mice (including resulting MAGs) and (b) individual cultured bacterial strains, (2) snRNA-seq and bulk RNA-seq of intestinal tissue and (3) microbial RNA-seq of caecal contents collected from gnotobiotic mice have been deposited at the European Nucleotide Archive (ENA; https://www.ebi.ac.uk/ena) under accession number PRJEB61647.

## Code availability

The custom source code for annotation of bacterial genes and prediction of metabolic pathway presence/absence is available on GitHub at https://github.com/rodionovdima/PhenotypePredictor-2 (ref. ^54^) and has been accessioned on Zenodo at 10.5281/zenodo.13250652 (ref. ^55^). All other analyses presented utilize published algorithms with their accompanying methods and are referenced as such.
