# Supplementary material for: Enteropathy produced in mice by intergenerational transmission of small intestinal microbiota from undernourished children
Source: Nat Microbiol. 2026 Jun 16;11(7):1967–81. doi: 10.1038/s41564-026-02394-4 (PMC13323066; doi:10.1038/s41564-026-02394-4)
Supplement: Supplementary file 1 — Supplementary Results, Discussion, Methods, Figs. 1–13 and References. [file 41564_2026_2394_MOESM1_ESM.pdf]

nature microbiology

Supplementary information

https://doi.org/10.1038/s41564-026-02394-4

Enteropathy produced in mice by intergenerational transmission of small intestinal microbiota from undernourished children

In the format provided by the authors and unedited

Supplementary Results

Initial tests of consortia of duodenal bacteria cultured from children from the BEED study

In a previous pilot study[1], a collection of 184 bacterial isolates cultured from the duodenal aspirates of children in the BEED study were pooled and introduced by oral gavage directly into adult germ-free mice fed a diet formulated based on foods consumed by children living in Mirpur, Bangladesh, the urban slum where the clinical study was performed ('Mirpur-18' diet, **Supplementary Table 1a**). Compared to controls gavaged with intact cecal contents from conventionally-raised mice (conventionalized animals, CONV-D), the duodenal aspirate-derived culture collection induced an enteropathy characterized by (i) patchy immunoinflammatory infiltrates in the small intestine, (ii) increased duodenal crypt depth, (iii) reduced expression of tight junction proteins and increased expression of genes involved in anti-microbial defense (*Reg3β* and *Reg3γ*) in the duodenum, and (iv) elevated levels of matrix metalloproteinase 8 (MMP8) in serum as well as along the length of the small intestine[1].

The two bacterial consortia cultured from children with EED were gavaged into separate groups of just-weaned 4-5-week-old germ-free mice (n=5 animals/group) fed the Mirpur-18 diet. Mice colonized with all 184 isolates gained significantly less weight over 28 days than mice colonized with the species-representative subset (**Supplementary Fig. 1a, Supplementary Table 2a**). Serum levels of insulin-like growth factor 1 (IGF-1) were significantly correlated with body mass at 9 and 28 days after colonization across all animals (**Supplementary Fig. 1b**), although there were no statistically significant differences between treatment groups (**Supplementary Table 2b**). Leptin levels were diminished compared to CONV-D animals, but not significantly different between mice that received the 184-member and species-representative consortium (**Supplementary Fig. 1c, Supplementary Table 2b**), suggesting that additional factors contributed to the reduced weight gain observed in animals that received the complete consortium.

Sequencing of RNA isolated from the duodenums, jejunums and ileums of these animals followed by gene set enrichment analysis (GSEA) revealed that a large proportion of the gene ontology (GO) categories that were significantly enriched ($q<0.05$) between animals colonized with the full versus subset bacterial consortia were related to immune function, including genes involved in the anti-bacterial defense response and leukocyte activation (**Supplementary Figs. 1d,e**). Levels of the protein lipocalin-2 (LCN2/NGAL; neutrophil gelatinase-associated lipocalin) were also significantly elevated in the serum of mice colonized with the full 184-isolate consortium (**Supplementary Fig. 1f**); previously we had observed that LCN2 levels in the duodenal mucosa of children with EED were strongly correlated with the absolute abundances of 14 EED-associated 'core taxa'[1]. Compared to animals colonized with the species-representative subset community, mice colonized with the full consortium of EED-derived bacteria harbored higher levels of intestinal and systemic inflammation. We named these consortia 'child small intestinal non-inflammatory' (cSI-N) and 'child small intestinal inflammation-inducing' (cSI-I), respectively.

Dam-to-pup transmission of EED-derived bacterial consortia

We identified bacterial members of each consortium that successfully colonized recipient dams and were transmitted to their offspring using long-read shotgun DNA sequencing of cecal contents from dams and P37 pups followed by assembly of metagenome-assembled genomes (MAGs) (n=4 dams and n=22 cSI-I, n=25 cSI-N P37 offspring). We reasoned that this approach would be useful for initial characterization of colonizing members of culture collections where multiple strains of a given species may exist. A total of 51 unique high-quality MAGs (defined as ≥85% complete and ≤5% contaminated based on marker gene analysis, **Supplementary Table 1c**) were generated from cecal contents of dams and P37 offspring harboring either of the two cSI consortia. A total of 25 MAGs from the cSI-I consortium colonized dams and all were represented in their P37 offspring (threshold criteria for colonization: >0.0001% relative abundance in at least one intestinal segment in at least 50% of animals, **Supplementary Table 1d, Supplementary Fig. 2a**). Twenty-eight MAGs from the cSI-N consortium colonized dams; all 28 also colonized their P37 offspring (**Supplementary Table 1d, Supplementary Fig. 2a**). Based on short-read shotgun sequencing-based calculations of MAG abundances in fecal samples collected from dams, the cSI-I and cSI-N communities remained stable between 4 and 17 weeks after gavage of these cultured consortia even if the animals had one or more litters during this period (P=0.09, PERMANOVA; n=4 dams/group; **Supplementary Figs. 2b,c**).

Fewer MAGs were detected in P14 offspring (88% of MAGs that colonized cSI-I dams; 68% of cSI-N MAGs that colonized dams). Of the 21 MAGs with significantly higher absolute abundance in P37 cSI-I offspring compared to their cSI-N counterparts, seven were significantly higher in the cecum and/or colon of P14 offspring (**Supplementary Fig. 2d**).

Testing the role of diet in mediating systemic inflammation

To determine whether inflammation induced by the cSI-I consortium was diet-dependent, we performed a diet oscillation experiment. Adult germ-free animals fed a standard mouse diet were colonized with either the cSI-I or cSI-N consortium.

Mice were maintained on standard chow for one week, then fed a diet designed to be representative of that consumed by adults residing in the Mirpur district of Dhaka ('Adult Mirpur' diet; **Supplementary Table 1a**), followed by a return to standard diet for one week. LCN2 levels were measured in serum samples obtained at the end of each of the 7-day diet periods (**Supplementary Fig. 3a**). Statistically significant increases in systemic inflammation were documented in both groups of mice when fed Adult Mirpur compared to standard mouse diet. Moreover, LCN2 levels were significantly higher in mice colonized with cSI-I compared to cSI-N in both diet contexts (**Supplementary Fig. 3b, Supplementary Table 2b**), suggesting that the Adult Mirpur diet exacerbates systemic inflammation.

Analysis of fecal samples collected longitudinally disclosed that 19 of the 21 MAGs with abundances that were significantly higher in cSI-I compared to cSI-N P37 offspring (**Fig. 1b**) were also significantly higher in adult mice when fed either Adult Mirpur or standard mouse diets (**Supplementary Figs. 3c,d**).

P37 offspring metabolic phenotypes

Bone biology – Compared to P37 cSI-N and CONV-D mice, serum levels of Dickkopf-1 (DKK1, P=0.009 and P=0.014, respectively), Osteoprotegerin (OPG, P=0.008, P=0.0006) and Fibroblast Growth Factor-23 (FGF23, $P=2.01\times10^{-5}$, $P=1.4\times10^{-5}$) were significantly higher in cSI-I animals, indicating increased osteocyte and bone remodeling activity (two-sided Wilcoxon rank-sum tests; **Supplementary Table 2b**). Micro-computed tomography of femurs disclosed significantly increased cortical tissue mineral density in P37 cSI-I versus cSI-N animals (P=0.03, two-sided Wilcoxon rank-sum test; n=19-21 mice/group); no significant differences in other cortical and trabecular parameters were found between the three treatment groups (**Supplementary Table 2d**).

Small intestinal tissue acylcarnitines – Plasma acylcarnitines are elevated in a fasting state and have been identified as a biomarker of EED in children[2]. Moreover, fecal acylcarnitines are elevated in the dysbiotic microbial states associated with inflammatory bowel disease[3] and can activate pro-inflammatory signaling cascades[4,5]. We quantified acylcarnitine levels in intestinal tissue instead of plasma to examine whether differences in host fatty acid metabolism occurred as a result of colonization with the cSI-I compared to the cSI-N consortium. Several medium and long-chain acylcarnitines were significantly elevated in the intestine of cSI-I compared to cSI-N or CONV-D offspring (**Supplementary Fig. 4a, Supplementary Table 2e**), suggesting that the carnitine shuttle may be impaired in the intestine.

Intestinal epithelial cellular responses to intergenerational transmission of the cSI-I and cSI-N consortia

Single nucleus RNA-sequencing (snRNA-Seq) – To characterize the responses of different intestinal epithelial cell types to the EED donor-derived bacterial consortia, we performed snRNA-Seq on intact segments of frozen duodenum and ileum harvested from P37 cSI-I and cSI-N mice. A total of 15,135 duodenal nuclei (**Supplementary Fig. 5a**) and 20,584 ileal nuclei (**Supplementary Fig. 5b**) collected from 3 littermates/group passed our quality metrics (see *Methods*). Analysis of marker gene expression identified nuclei from epithelial lineages (stem and transit amplifying cells and their enterocyte, goblet, Paneth, enteroendocrine and tuft cell descendants), as well as from mesenchymal lineages (endothelial, smooth muscle, neuronal, immune and interstitial cells of Cajal). Enterocytes comprised the largest proportion of nuclei (**Supplementary Table 3b**) and based on marker gene expression were divided into villus base, mid-villus and villus tip subpopulations. A significantly higher proportion of Paneth cells were present in the duodenum of cSI-I offspring than their cSI-N counterparts (**Supplementary Table 3b**). Increased density[6] of Paneth cells as well as degranulation[7] have both been reported in Zambian children with EED.

'Pseudo-bulk' analysis of differential gene expression in enterocytes positioned at different locations along the crypt-villus axis in both the duodenum and ileum revealed an enhanced epithelial immune response in P37 cSI-I compared to cSI-N mice. Of the 133 GO categories significantly enriched in at least two subpopulations of enterocytes in cSI-I offspring, 35 were involved in bacterial recognition and defense, as well as recruitment/migration and activation of immune cells (**Supplementary Fig. 5c, Supplementary Table 3c**).

Epithelial homeostasis requires a balance of differentiation, programmed cell death, and clearance of dying cells and cellular debris. This process, termed 'efferocytosis', involves recognition and clearance of dying cells by phagocytes, including both professional phagocytes and healthy neighboring intestinal epithelial cells[8–10]. Efferocytosis can be viewed as a process that sits at the interface between regulation of gut epithelial renewal, inflammation and the microbiota[11]. Based on published literature[12–14], we manually curated an 'efferocytosis' gene set composed of 273 genes. This gene set was significantly enriched in cSI-I mid-villus duodenal enterocytes (P=0.019, **Supplementary Fig. 5d**). Genes enriched in cSI-I duodenal enterocytes included caspases linked to apoptosis (*Casp3*), genes linked to phosphatidylserine exposure during apoptosis (*Xkr8, ATP11c*), a channel that regulates apoptotic cell communication with the microbiota (*Panx1*),

several efferocytotic receptors/recognition mediators (*Mertk, Stab1*, *Mfge8*), as well as cytosolic proteins linked to intracellular signaling during efferocytosis (*Elmo1, Elmo2, Dock1, Crk*, see **Supplementary Table 3c**). These findings suggest that apoptosis and subsequent efferocytosis within the small intestine play a role in shaping the epithelial response to the cSI-I consortium.

***Proliferative signaling to stem and transit-amplifying cells* –** To assess the effects of the SI consortia on epithelial regeneration, we used the algorithm *NicheNet*[15,16] to characterize intercellular signaling in cSI-I versus cSI-N P37 offspring. *NicheNet* surveys expression of known protein ligands by user-designated 'sender' cell types and their cognate receptors within a 'receiver' population. For our analysis, we defined stem and transit amplifying (stem/TA) cells as 'receivers' and all other cell types within the SI as 'senders' (**Supplementary Fig. 6a**). Bone morphogenetic protein (Bmp), Wnt and Notch signaling are three of the major pathways that mediate cellular proliferation during normal intestinal development, responses to injury and hyperplastic inflammatory responses[17,18]. In the ileum, ligands more highly expressed in cSI-I compared to cSI-N mice included *Wnt3* in Paneth cells (and their receptors *Lrp6, Ryk* and *Bmpr1a* in TA/stem cells, **Supplementary Fig. 6b, Supplementary Table 3c**), as well as Paneth cell *Bmp7* (and its *Bmpr1a* and *Bmpr2* receptors in stem/TA cells). *Bmp6* and *Bmp2,* two other ligands for *Bmpr1a* and *Bmpr2* receptors, were also expressed at higher levels in ileal cSI-I goblet cells (**Supplementary Figs. 6a,b**). Expression of *Wnt5a* and the Wnt inhibitor *Dkk2* were significantly diminished in ileal smooth muscle cells (SMCs) in cSI-I mice, as were their receptors in stem/TA cells (*Cftr* and *Ptprk* in the case of *Wnt5a* and *Lrp6* in the case of *Dkk2*) (**Supplementary Figs. 6a,b, Supplementary Table 3c**). SMC-derived *Wnt5a* augments Th2 immunity and inhibits cellular proliferation by downregulating expression of *Ctnnb1* (ß-catenin)[19]. Quantification of Ki67, a marker of cellular proliferation, in duodenal and ileal crypts provided additional evidence of increased epithelial regeneration in cSI-I animals (**Supplementary Figs 6c,d**; **Supplementary Table 2c**).

Contextualizing intestinal transcriptional responses with proteomic features in duodenal mucosa of children with EED – To further assess the degree to which the transcriptional response in enterocytes present in the duodenum and ileum of P37 offspring recapitulates pathophysiologic changes we had documented in the upper gastrointestinal tract of Bangladeshi children with EED[1], we performed GSEA with mouse homologs of duodenal mucosal proteins that were: (i) negatively correlated with length-for-age-Z-score (LAZ) and positively correlated with the levels of core EED bacterial taxa in BEED study participants, or (ii) positively correlated with LAZ in these children and negatively associated with core EED taxa (**Supplementary Fig. 7a**). Expression of mouse genes homologous to the human duodenal proteins that were negatively associated with growth and positively correlated with the absolute abundances of core taxa were enriched in cSI-I enterocytes in the duodenum (crypt through mid-villus) and in the ileum (crypt and along the length of the villus) (**Supplementary Fig. 7a**). Genes encoding antimicrobial peptides (*Reg3β* and *Reg3γ*), the pro-inflammatory cytokine *Il18*, the heat-shock protein *Hsp90aa1* and the immune cell chemoattractant *Ccl28* were more highly expressed in all duodenal enterocyte cell types, whereas the type-2 immune response regulator *Arg-2* was more highly expressed in cSI-N offspring (**Supplementary Fig. 7b, Supplementary Table 3c**). Together, these results provide evidence that SI enterocytes play an important role in mediating the immune response in offspring of dams colonized with the cSI-I consortium.

C. concisus depends on community members to colonize and induce inflammation

After determining that *C. concisus* induced inflammation in the context of the cSI-N community, we tested whether *C. concisus* alone could induce intestinal and systemic inflammation. We mono-colonized adult germ-free mice with 10^7 CFUs via oral gavage on experimental days 0, 3, 5, 7, and 14. Two groups served as controls: animals orally gavaged with 10^7 CFUs of heat-killed *C. concisus* on the same days, and another group that was maintained as germ-free (6-7 mice/group). After 18 days, qPCR assays disclosed $10^{2.7}$-$10^{5.8}$ organisms/ng DNA in cecal and/or colonic contents in mice gavaged with the live organism; levels were below the limits of detection (10^2 genome equivalents) in the duodenum, jejunum, and ileum of all mice gavaged with live *C. concisus* and for all members of the two control groups (**Supplementary Table 1e**).

The abundance of *C. concisus* in cecal contents of mono-colonized animals was not significantly different from that in cSI-N mice (**Extended Data Fig. 4g, Supplementary Table 1e**). Moreover, despite two additional gavages, levels of *C. concisus* achieved in mono-colonized mice were substantially lower than in mice from the add-in experiment (118- and 131-fold compared to mice originally colonized with cSI-N then gavaged with *C. concisus* ± the *Actinomyces* strains, respectively; 167-fold lower than in cSI-I mock-gavaged controls) (**Extended Data Fig. 4g, Supplementary Table 1e**). There were no statistically significant differences in duodenal, ileal, colonic, or serum levels of LCN2 nor CHI3L1 between germ-free mice and mice gavaged with heat-killed or live *C. concisus* (P>0.05; repeated measures ANOVA;

Supplementary Table 2b). These findings indicate that levels of *C. concisus* colonization and its capacity to elicit an inflammatory response are community-context dependent.

***C. concisus* comparative genomic analysis**

Some strains of *C. concisus* decreased expression of tight junction proteins and increased permeability to FITC-dextran in a cultured human adenocarcinoma-derived, enterocyte-like cell line (HT-29)[20,21]. Searches of the Bangladeshi *C. concisus* Bg048 isolate genome and corresponding MAG048 failed to identify known *C. concisus* virulence factors including *Zot* (Zonula occludens toxin), which has been found in 30% of *C. concisus* strains[22] and reported to disrupt intestinal epithelial tight junctions and augment IL-8 production in HT29 cells[44,47]. Nonetheless, searches within Bg048 and corresponding MAG for homologs to well-described bacterial virulence factors using the Virulence Factor Database (VFDB[23]) identified genes involved in chemotaxis (the chemotaxis regulator *cheY*, flagellar motor switch proteins *fliM* and *fliN*, and flagellum-specific ATP synthase *fliI*) that were conserved with *Campylobacter jejuni* (**Supplementary Table 4a**).

We performed a follow-up comparative genomics analysis that included 119 additional *C. concisus* strains isolated from individuals with oral and/or gastrointestinal pathology (periodontitis, gastroenteritis, Ulcerative Colitis, Crohn's Disease, Inflammatory Bowel Disease), or healthy controls[24,25] (**Supplementary Fig. 8**). We performed a functional enrichment analysis that focused on functions (Clusters of Orthologous Genes, COGs) or genes that were enriched in isolates obtained from diseased compared to healthy individuals. COG20 functions and pathways, including genes involved in bacterial defense, bacterial cell wall modification, immune signaling/subversion and Type IV pilus assembly, were significantly enriched in the Bg048 strain and other isolates obtained from diseased compared to healthy hosts (**Supplementary Table 4c**). Our analysis also disclosed that genes encoding a nitrate-inducible formate dehydrogenase (*fdnG*) and formate dehydrogenase H (*fdhF*) were unique to the Bg048 isolate (**Supplementary Fig. 8**). In addition, this strain and only one other among the 119 surveyed contained a *selD* (selenide water dikinase) which, through its capacity to produce selenocysteine, may provide the required selenium cofactor for these formate dehydrogenases[26] (**Supplementary Fig. 8**, **Supplementary Table 4c**).

Intestinal epithelial-derived substrates boost *C. concisus* Bg048 growth

C. concisus transcriptional profiling – *C. concisus* is known to be non-saccharolytic and exhibit preferences for simple carbon sources (e.g., fumarate, DMSO) and hydrogen gas for growth. Our *in silico* metabolic predictions indicated that *C. concisus* Bg048 has broad respiratory capacity, including for nitrate, nitrite and nitrous oxide (**Supplementary Table 4b**)[27].

To garner clues about substrates that support *C. concisus* growth in the gut, we conducted RNA-seq analyses of the cecal microbial community of mice from our 'add-in' experiment (**Fig. 3**) and of *C. concisus* grown *in vitro* under anaerobic conditions in rich medium (Bolton Broth with and without 1% mucin). Compared to growth *in vitro* in both media conditions, genes involved in (i) motility (Flagellin A *flaA* and secreted flagellin *flaC*, flagellar ring proteins *flgI/flgH*, assembly factor f*liW*, biosynthetic proteins *fliQ/fliP*, basal body rod protein *flgC*, motility protein *motB*, chemotaxis proteins *cheW*, *cheA*, *mcpH*), (ii) oxidative stress response (superoxide dismutases, *sodB/sodC*), and (iii) several genes encoding respiratory proteins (dimethyl sulfoxide *dorA_2*, *dmsA_1*, *dmsB*, tetrathionate *ttrA/B*, thiosulfate *phsB*, and fumarate *fdrA/B*, *ifcA_2* reductases; formate dehydrogenases *fdhF_3/fdhF_4, fdhB1_2* and hydrogenlyases *hycD/hycG/hycE*) were more highly expressed in cSI-N/*C. concisus*-colonized mice (**Supplementary Fig. 9a**, **Supplementary Table 5a**).

We then compared *C. concisus* gene expression between four groups of mice: cSI-I, cSI-N/*C. concisus*, cSI-N/*C. concisus+Actinomyces* and cSI-N/mock controls (the latter contain significantly lower levels of *C. concisus*) (**Supplementary Fig. 9b**). The transcript with the largest increase in expression in cSI-N/*C. concisus* mice compared to cSI-N/mock-gavaged animals was a periplasmic nitrate reductase (*napA*, **Figs. 5b,c**). A nitrate import protein (*nrtD*) was also significantly more highly expressed in cSI-N/*C. concisus* mice, suggesting that nitrate may play a role in *C. concisus* colonization and/or virulence (**Fig. 4c**, **Supplementary Table 5b**). Genes involved in (i) motility (*motB*, twitching mobility proteins *pilT_1/pilT_2*, flagellar proteins *flgB, flgG_1/flgG_2, flgH, flgI, flhA, flhB_1/flhB_2, fliG, fliM, fliN, fliP, fliQ, fliR, ylxH*), (ii) redox homeostasis/survival (*sodB*, cytochrome c551 peroxidase *ccpA*, peroxidase *yfeX*, thiol peroxidase *tpx*), and (ii) formate metabolism (*fdhF_1/fdhF_2, hycE*, formate-tetrahydrofolate ligase *fhs*) were also more highly expressed by *C. concisus* in inflammatory conditions (**Supplementary Fig. 9b, Supplementary Table 5b**).

Products of human colonic epithelial cells increase* C. concisus *growth – Having determined that in the context of the cSI-N community *C. concisus* induced an immunoinflammatory response, we tested the hypothesis that *C. concisus* may utilize host substrates produced under conditions where there is inflammation and perturbed intestinal epithelial turnover.

To do so, we first turned to an *in vitro* tissue culture system in which we cultured mouse- and human-derived colonic epithelial cell lines (CT26 and HTC116, respectively) with or without subsequent induction of cell death (**Supplementary Fig. 10a**, see *Methods*) and then collected conditioned medium[11]. *C. concisus* growth in the presence of oxygen (5% O_2) was significantly higher in spent medium from live mouse CT26 cells (**Supplementary Figs. 10b,c, Supplementary Table 6a**) and human HTC116 cells (**Supplementary Figs. 10d-f, Supplementary Table 6a**) compared to conditioned medium harvested from cells undergoing apoptosis or to cell culture medium alone. When cultured anaerobically, *C. concisus* growth did not differ in spent medium from cells that were alive or undergoing apoptosis and was diminished compared to growth in cell culture medium alone (**Supplementary Figs. 10g,h, Supplementary Table 6a**). These findings suggest that changes in *C. concisus* metabolism in the presence of low levels of oxygen, such as those that occur in the oxidative state of the inflamed gut[28], may underlie a fitness benefit conferred to *C. concisus* through utilization of host-derived substrates.

During an inflammatory response, nitric oxide produced by inducible nitric oxide synthase (iNOS, encoded by *Nos2*) reacts with reactive oxygen species to form nitrate[29]. Other Proteobacteria have been shown to utilize host-derived nitrate[29–31] or other byproducts of iNOS activity[65] to gain a competitive advantage in mouse models[32]. Given the strong up-regulation of nitrate reductase by *C. concisus* in the gut under inflammatory conditions (cSI-I, cSI-N/*C. concisus*, cSI-N/*C. concisus+Actinomyces*) compared to non-inflammatory states (cSI-N mice) (**Figs. 5b,c**), we examined whether *C. concisus* could utilize host-derived substrates generated through metabolism of nitric oxide.

Nitrate in ileal tissue was significantly higher with addition of *C. concisus* alone (cSI-N/*C. concisus*) compared to cSI-N and cSI-I sham-gavaged controls as well as when *Actinomyces* were present (cSI-N/*C. concisus+Actinomyces*) (**Fig. 4b, Supplementary Table 2g**). Nitrate reductase activity reduces nitrate to nitrite; increased bacterial nitrate reductase activity should result in elevated luminal nitrite. Nitrite in cecal contents trended higher in *C. concisus*-gavaged animals (**Supplementary Fig. 11a, Supplementary Table 2g**), although the difference was not statistically significant, perhaps due to microbial reduction of nitrite or the short half-life of nitric oxides.

C. concisus utilizes produces of host nitric oxide metabolism – To test whether epithelial cell nitric oxide generation contributed to the increase in *C. concisus* growth in spent medium collected from live cells, we treated HTC116 cells with the non-selective NOS inhibitor N-iminoethyl-L-ornithine (L-NIO)[33]. L-NIO treatment significantly reduced the growth benefit conferred to *C. concisus* by exposure to conditioned medium collected from live human colonic epithelial cells after 24 hours growth in microaerophilic atmospheric conditions (**Fig. 4d, Supplementary Fig 11b, Supplementary Table 6a**). L-NIO treatment did not alter anaerobic growth in spent medium from colonic epithelial cells compared to culture medium alone (**Supplementary Figs. 11c, Supplementary Table 6a**). Together, these results suggest that host nitric oxide metabolism boosts growth of *C. concisus* and may confer an advantage under conditions where low levels of oxygen are present in the gut, as is the case with inflammation.

We subsequently profiled *C. concisus* gene expression during growth under microaerophilic conditions in conditioned medium from live HTC116 cell cultures, or the cell culture medium (DMEM). A putative formate transporter (*focA*), formate dehydrogenase (*fdhB*) and formate hydrogen lyases (*hycE, hycD*), a periplasmic nitrate reductase *(napA)*, as well as other genes involved in anaerobic respiration were significantly more highly expressed when grown in the presence of live HTC116 spent medium compared to medium alone (**Supplementary Fig. 11d, Supplementary Table 5c**). Importantly, the periplasmic nitrate reductase was not significantly differentially expressed when *C. concisus* was grown in conditioned medium from HTC116 cells treated with the NOS inhibitor L-NIO compared to medium alone (DMEM) while other genes involved in anaerobic respiration were still differentially expressed in the presence L-NIO, including those involved in formate metabolism (**Supplementary Table 5c**).

Formate pre-treatment increases* C. concisus *virulence – As noted above, when *C. concisus* was grown under microaerophilic conditions in spent medium from live HTC116 cell cultures, a nitrate-inducible formate transporter (*fdnG*) and formate transporter (*focA*) were expressed at significantly higher levels compared to medium alone (**Supplementary Fig. 12a**).To determine whether formate plays a role in *C. concisus*-induced inflammation, as has been described for other pathogens, including *Campylobacter jejuni*[34–36], we stimulated bone marrow cells with *C. concisus* that had been grown with and without formate. While formate did not increase *C. concisus* growth (**Supplementary Fig. 12b, Supplementary Table 6c**); (i) live (but not heat killed) *C. concisus* depleted formate when co-cultured with WT or *Nos2*$^{-/-}$ bone marrow cells (**Supplementary Fig. 12c Supplementary Table 6c**) and (ii) *C. concisus* pretreated with 10 mM formate significantly increased IL-1β produced by bone marrow cells (**Supplementary Fig. 12d, Supplementary Table 6c**). Collectively, these findings point to inter-related pathways involving nitric oxide metabolism, formate, and effector cytokines in the host response to *C. concisus*.

Supplementary Discussion

Deciphering the role of the small intestinal microbiota in the pathogenesis of EED has been hampered by several factors. For example, EGD is limited to the proximal intestine and carries potential risks, precluding, for ethical reasons, its use in healthy children to define what constitutes a 'normal' small intestinal microbiota. In addition, the volume of fluid and biomass of microbes that can be retrieved from the lumen of the small intestine with EGD is small, limiting the number of ways the material can be employed for downstream analyses.

Our pre-clinical intergenerational gnotobiotic model, combined with shorter-duration manipulation of community membership using cohousing and single strain manipulations, allowed for testing the role of specific members of the cSI-I consortium in mediating the physiologic effects observed in dams and their offspring. If the cSI-I and cSI-N consortia had contained unique strains, we would have been able to track the provenance of strains as the two consortia mixed in cohoused mice (and define whether strains 'bloomed' or 'invaded'). However, as the same strains were present in both cSI-I and cSI-N, we are unable to distinguish the origin of strains in cohoused animals. Regardless, from the results of our cohousing experiments, we were able to associate three MAGs – *C. concisus*, *A. naeslundii* and *A. odontolyticus*, all members of the oral microbiota – with development of pathology. Follow-up experiments in which these candidate disease-promoting isolates were introduced to gnotobiotic animals colonized with the non-pathology-inducing cSI-N consortium defined *C. concisus* as a key driver in inducing intestinal inflammation. These experiments will also provide an opportunity for more detailed physiological profiling, including comprehensive energy balance studies, to ascertain the origin of the weight loss phenotype observed in cSI-I mice.

Disruption of the intestinal microbiota by taxa that normally reside in the oral microbiota has been implicated in studies of undernutrition and EED[37,38]. Evidence for this process, referred to as 'decompartmentalization' of the microbial community, has come from applying culture-independent methods (primarily16S rRNA amplicon sequencing) to feces. As such, there is little information about disease associations with specific strains, or correlations with their genome-encoded functional features.

It is intriguing that addition of this EED donor-derived *C. concisus* strain to mice harboring the EED duodenal-derived cSI-N consortium of cultured bacteria produced an immunoinflammatory response that was greatest in the colon – at least within the relatively short duration of the cohousing and 'add-in' experiments, which were conducted in coprophagic mice. These findings prompt the question of what factors allow it (and other members of the oral microbiota) to establish themselves in distal regions of the intestine.

C. concisus has been reported to be over-represented in the fecal microbiota of stunted compared to non-stunted children living in South India, and significantly more abundant in the feces of a cohort of stunted children living in sub-Saharan Africa compared to healthy controls[39,40]. In children of the BEED study, the only *Campylobacter* ASV present correlated significantly with duodenal mucosal levels of CD177, a cell surface glycoprotein involved in neutrophil activation, and was significantly more prevalent in children with histopathologic evidence of epithelial damage. *C. concisus* has been described as a typical member of the oral microbiota in healthy individuals (97% prevalence in a cohort of 59 individuals aged 3-80 years)[41], but it has also been associated with gingival disease[42] and inflammatory bowel disease (IBD)[42,43]. The distinctions between *C. concisus* strains recovered from the oral and intestinal microbiota of healthy individuals versus those with immunoinflammatory conditions remain to be fully defined.

Our 'add-in' experiments indicate that *C. concisus* induces higher levels of inflammation after a longer period of colonization – rather than being cleared by the host. Canonical enteric pathogens, including *Salmonella*[11], have been shown to utilize substrates from dying, rather than live, intestinal, epithelial cells, as is the case for *C. concisus*. The question of what the initiating events are that allow a pathogen/pathobiont to first establish itself in a microbial community and induce inflammation is complex. One approach is to use genetic tools to identify the contributing signaling and metabolic pathways in both the microbe and host. Our model provides a justification for and sets the stage for these types of detailed analysis.

Supplementary Methods

Mouse Experiments

Husbandry for initial tests of bacterial consortia derived from children with EED – 4–5-week-old germ-free C57Bl/6J mice were given *ad libitum* access to a standard chow diet (Diet 2018S, Envigo). Three days prior to colonization, mice were switched to the Mirpur-18 diet. For initial comparisons of gnotobiotic mice that had been orally gavaged (oral gavage needle; Cadence Science; catalog no. 7901) with the cSI-I consortium, the cSI-N consortium or the cecal contents of conventionally-raised C57Bl/6J animals that had been maintained on the standard chow diet, one group of animals per

treatment arm was euthanized 9 days after gavage (n=5 mice/group), and the second group 28 days later (n=5 mice/group). All animals were weighed and fecal samples were collected three times per week.

To test of the role of diet in mediating inflammation, adult female germ-free mice were given breeder chow (Lab Diet 5021) *ad libitum* prior to oral gavage with the cSI-I or cSI-N consortium. Mice were fed breeder chow for one week prior to switching to the Adult Mirpur diet for the following week and then returned to breeder chow for the final week. Blood was collected from all animals in their isolators retro-orbitally prior to a diet switch (n=3 animals/colonization group euthanized at the end of 7 days on a given diet; the remaining n=5 animals/group were euthanized at 28 days.

Micro-computed tomography (μCT) of bone – The femur was harvested from the left rear leg and cleaned of muscle and connective tissue. Femurs were stored in 10 mL 10% neutral buffered formalin at room temperature for 24 hours. Fixed femurs were then washed in 1X PBS for 15 minutes; this wash step was repeated two more times. Femurs were then washed in 30% ethanol for 30 minutes, 50% ethanol for 30 minutes, and 70% ethanol for 30 minutes. Femurs were subsequently embedded in 2% agarose and scanned with a μCT 40 desktop cone beam instrument (ScanCO Medical, Brüttisellen, Switzerland). For analyses of cortical bone, 100 slices were taken for each sample in the transverse plane, with a 6 μm voxel size (high resolution); slices began at the midpoint of the femur and extended toward the distal femur. The boundaries and thresholds for bone were drawn manually using μCT 40 software. Volumetric parameters were quantified using software associated with the ScanCO instrument.

Acylcarnitine quantification – Acylcarnitines were quantified in 1 cm segments of small intestinal (jejunal) tissue from P37 offspring using methods described previously[44,45]. Briefly, frozen tissue was weighed and extracted in (v/v) 50:50 acetonitrile:0.3% formic acid in water (v/v) at 50 mg/mL (w/v). Intestinal tissue samples were homogenized in Lysing Matrix D (MP Biochemicals, 6913050) tubes with 1 minute bead-beating/1 minute off (on ice) until no visible tissue pieces remained. Samples were centrifuged at 2,000 x *g* at 4 °C for 3 minutes, incubated on wet ice for 1 minute prior to transfer of the supernatant to a plastic 2 mL screw-cap tube, which was stored at -80 °C until quantification.

Metagenome-assembled genomes (MAGs)

In silico metabolic reconstructions of MAGs and isolate genomes – MAGs and isolate genomes were initially annotated using *prokka* (v1.14;[46]); KEGG Orthology (KO) numbers were assigned to bacterial coding sequences using BlastKOALA[47]. MAGs were then subjected to *in silico* metabolic pathway reconstruction. Our approach for reconstruction is based on (i) gene annotation using the microbial community SEED (mcSEED) database containing 2,856 reference bacterial genomes for which metabolic pathways have been inferred from expert curation and (ii) subsequent *in silico* predictions of the presence or absence of the pathway in each genome[48,49]. These results capture utilization and/or biosynthesis of 106 metabolites, including carbohydrates, amino acids, vitamins, and fermentation end-products[50–52].

For functional annotation of coding sequences in each genome, we used a combination of public domain tools (such as DBSCAN, DIAMOND, MMSeq2), custom scripts and the mcSEED reference database genes. These annotations were supplied to a 'Phenotype Predictor' pipeline that allows automated propagation of curated metabolic phenotypes over new microbial genomes and MAGs from represented phylogenetic groups using a consensus of three complementary approaches: (i) rule-based phenotype assignment using the genomic distribution of orthologs and a set of phenotype rules; (ii) machine learning models for binary phenotype prediction trained on reference sets of genes and phenotypes and (iii) a neighbor-based approach for phenotype assignment based on variability of metabolic phenotypes and genes within a group of phylogenetically close neighbors. The combination of all three approaches allows consensus phenotype assignment with >99% accuracy[53]. The mcSEED gene annotations are provided in **Supplementary Table 4b**.

snRNA-Seq

Isolation of nuclei from small intestinal tissue – Nuclei were extracted from flash-frozen 1.5 cm pieces of duodenum and ileum (piece #2, see *"Division of the intestine"* above) collected from P37 mice in the intergenerational transmission experiment. Briefly, tissue was thawed and minced in lysis buffer (25 mM citric acid, 0.25 M sucrose, 0.1% NP-40, 1X protease inhibitor). Nuclei were released from cells using a Dounce homogenizer (Wheaton), washed 3 times with buffer [25 mM citric acid, 0.25 M sucrose, 1X protease inhibitor (Roche)], then filtered successively through 100 μm-, 70 μm-, 40 μm-, 20 μm- and 5 μm-diameter strainers (pluriSelect) to obtain single nuclei. Following filtration, nuclei were pelleted by centrifugation (500 x *g* for 5 minutes) and resuspended in a buffer containing 5 mM KCl, 3 mM $MgCl_2$, 50 mM Tris, 1 mM DTT, 0.4 U/μL RNase inhibitor (Sigma), plus 0.4 U/μL Superase inhibitor (ThermoFisher)[44].

Generation and sequencing of snRNA-Seq libraries – We used approximately 7,000 nuclei per intestinal sample for gel bead-in-emulsion (GEM) generation. Reverse transcription and library construction were performed according to the protocol provided in the 3' gene expression v3.1 kit manual (10X Genomics PN-1000121). Balanced libraries were sequenced on Illumina NovaSeq 6000 instrument [150 nt paired-end reads; $2.95 \times 10^8 \pm 2.02 \times 10^7$ reads/sample (mean ± SD)]. Sample metadata are listed in **Supplementary Table 8**.

Analysis of snRNA-Seq datasets

Preprocessing and quality control - Read alignment, feature-barcode matrices and quality controls were performed using the *CellRanger* 5.0 pipeline with the flag '--include-introns' (GRCm38/mm10). 'Ambient' RNA was removed using the *remove-background* module from CellBender[54]. Sample integration, count normalization, cell clustering and marker gene identification were performed using Seurat 4.0[55]. Briefly, filtered feature-barcode matrices from CellRanger were imported as a Seurat object using *CreateSeuratObject*. Matrices were filtered to remove low quality nuclei (defined as nuclei with < 200 or > 5000 genes or < 400 UMIs). Nuclei with over 5% reads from mitochondrial genes or over 5% reads from ribosomal protein genes were excluded. Each sample was then normalized using *SCTransform*[56,57] and predicted doublets were removed using *DoubletFinder*[58]. Samples of the same tissue type (duodenum or ileum) were integrated using *SelectIntegrationFeatures*, *PrepSCTIntegration*, *FindIntegrationAnchors* and *IntegrateData* from the Seurat software package. Each integrated dataset was subjected to unsupervised clustering using *FindNeighbors* (dimensions = 1:30) and *FindClusters* (resolution = 0.8 and 1.2 for duodenum and ileum, respectively) from the Seurat package.

Cell type annotation – Cell type annotation for duodenal and ileal snRNA-Seq objects was performed using the *FindMarkers* function in Seurat. Manual cell type assignments were conducted based on expression of reported markers[59].

Pseudo-bulk analysis of differential gene expression – Genes defined as having low levels of expression (read count < 4) were filtered out prior to count aggregation across nuclei for a given cell type (cluster) within each biological sample. Each pseudo-bulked sample served as input for *DESeq2*-based differential gene expression analysis (likelihood ratio test, minimum=1e-6[60]). Genes that were differentially expressed (adjusted $P < 0.05$) were used as input for GSEA with *clusterProfiler*[61].

Intercellular signaling – Signaling within each tissue (duodenum or ileum) was inferred with *'Differential' NicheNet*[15]. Briefly, log-transformed counts were used for as input for the wrapper function *nichenet_seratobj_aggregate*. Default ligands, receptors and target matrices were used. In the duodenum and ileum, all cell clusters were designated as 'sender' cells to stem and transit amplifying (TA) 'receiver' cells[16]. *Differential NicheNet* includes differential expression of ligands within a given 'sender' cell type between conditions (cSI-I vs. cSI-N) in the ranking of ligand-receptor pairs, while the *NicheNet* algorithm does not.

C. concisus **comparative genomics analysis**

Published *C. concisus* whole genomes (97 from refs[24], 14 from ref[25], 8 from NCBI taxid=199) were downloaded from NCBI through *anvi'o* (v8, ref[62]). A typical pan-genomic workflow was conducted. Briefly, whole genomes (.gbff files) and associated metadata were downloaded through their NCBI BioProject accessions or taxid numbers. Contig databases were generated with default config settings using the anvi'o snakemake workflow[63]; NCBI-PGAP was used as the gene caller to create a genomes storage database. For the pangenome analysis[64], ncbi-blast was used with *mcl-inflation*=10 and *minbit*=0.5. Genome similarity was computed with *pyANI*. Disease-associated metadata were manually curated and used to compute functional enrichment (*anvi-compute-functional-enrichment-in-pan*[63]). The statistical approach used to define enrichment scores for functions or genes fits a generalized linear model to the occurrence of each module and computes a Rao test statistic with multiple hypothesis correction; this approach has been described previously[63].

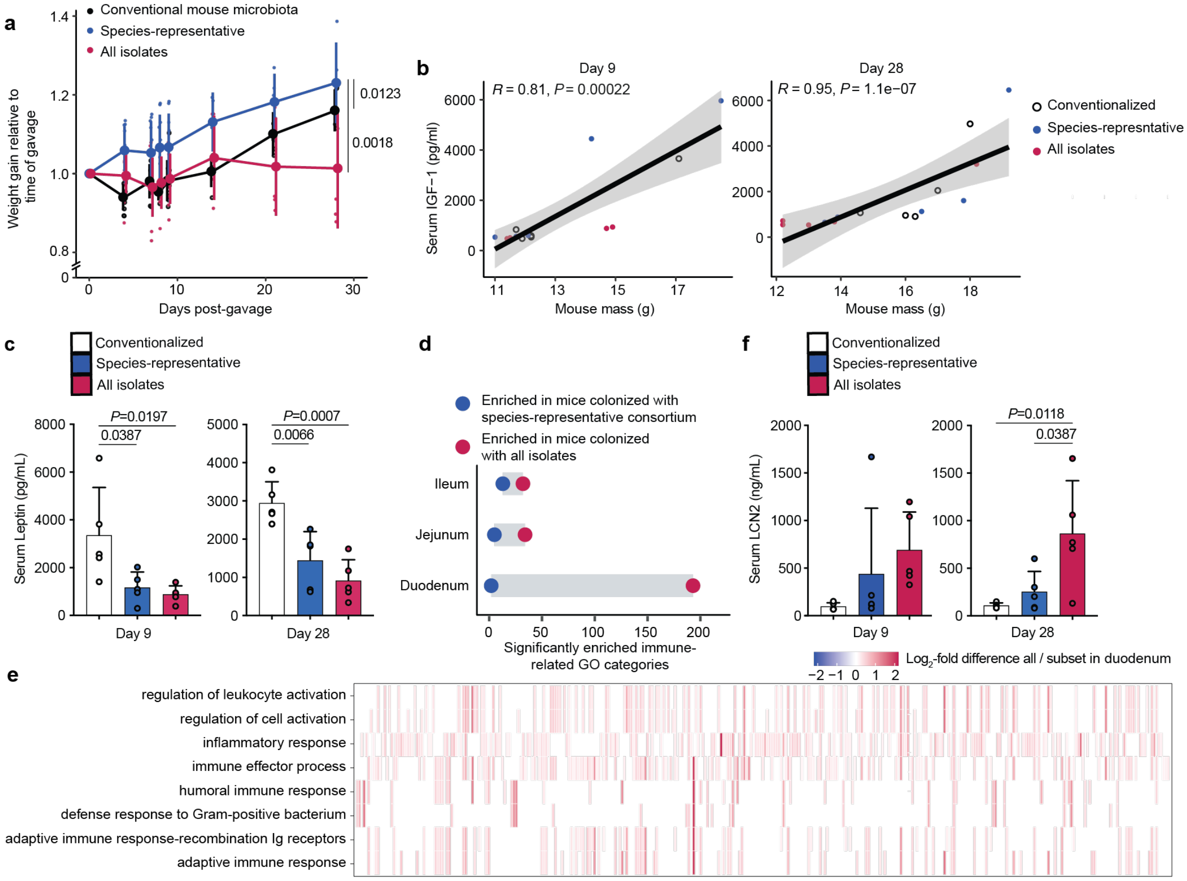

Supplementary Fig. 1. Initial test of the host response to colonization with the cSI-N and cSI-I consortia. a, Weight gain of mice colonized with all 184 isolates cultured from duodenal aspirates obtained from children with EED (cSI-I), or a species-representative set of isolates (cSI-N). A control group of CONV-D mice was colonized with cecal microbiota harvested from conventionally raised animals. All mice were fed Mirpur-18 diet *ad libitum*. Each small point represents the weight gain for an individual mouse; larger points denote the mean ± s.d.. Linear mixed effects model (Weight ~ Group*Day + (1|MouseID), *P*-values shown are the result of Tukey's post-hoc tests, n=10 mice/group until day 9, n=5 mice/group thereafter). **b,** Serum IGF-1 correlates with mouse weight measured 9 (left) or 28 (right) days after colonization (linear regression). **c,** Serum leptin levels determined 9 days (left, $F_{(2,12)}$=6.079, *P*=0.0150) or 28 days (right, $F_{(2,12)}$=14.21, *P*=0.0007) post-colonization (bars denote mean ± s.d., one-way ANOVA with Tukey's multiple comparisons). **d,** Number of Gene Ontology (GO) categories related to immune system function that were significantly enriched (GSEA *q* value < 0.05) based on differential gene expression in the small intestine of mice colonized with all isolates (red) versus the species representative subset (blue). Bulk RNA-seq was performed in duodenal, jejunal, and ileal tissue 28 days following gavage. **e,** Expression of genes (columns, un-labeled) that comprise the leading edge of the 8 GO categories most significantly enriched in animals colonized with the full consortium (red) compared to the species-representative subset (blue). Bulk RNA-seq of duodenal tissue, 28 days post colonization. **f,** Serum lipocalin-2 (LCN2) levels 9 days (left, $F_{(2,12)}$=2.091, *P*=0.1663) or 28 days (right, $F_{(2,12)}$=6.814, *P*=0.0105) post-gavage of the consortia (bars denote mean ± s.d., one-way ANOVA with Tukey's multiple comparisons). For **b-f**, n=5 mice/treatment group/timepoint.

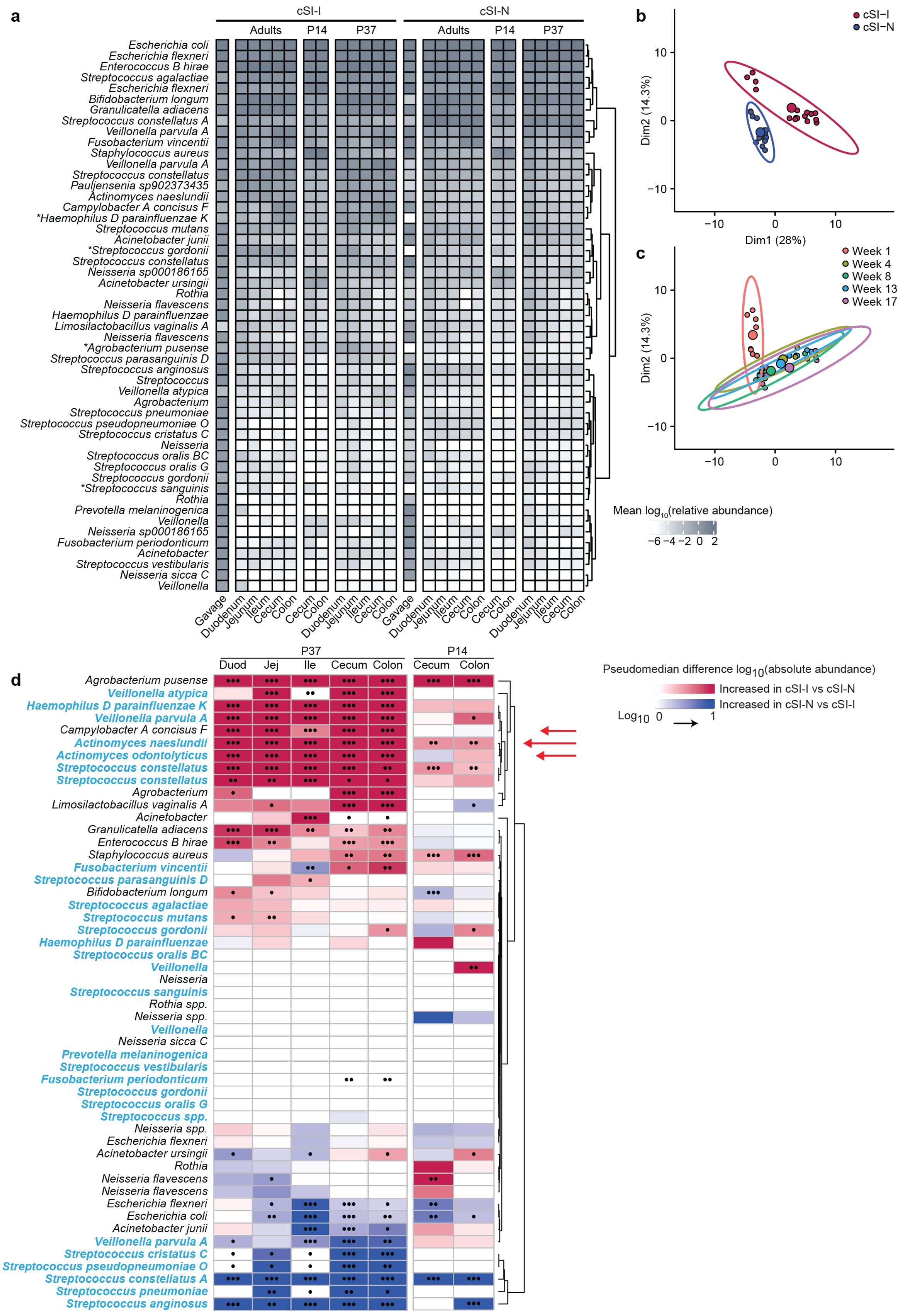

Supplementary Fig. 2. MAGs detected in dams and their offspring. a, Mean relative abundance ($\log_{10}$-transformed) of all MAGs that passed quality threshold parameters in input cSI-I and cSI-N gavage mixtures, the duodenum, jejunum, ileum, cecum, and colon of dams (n=7 cSI-I, n=5 cSI-N) and their P37 offspring (n=21 cSI-I, n=25 cSI-N), as well as the cecum and colon of P14 offspring (n=19 cSI-I, n=11 cSI-N). The asterisks denote four MAGs that were below the limit of

detection in the cSI-N gavage mixture at the sequencing depth used but nonetheless were detectable in (i) cSI-N dams and their offspring in this and independent follow-up experiments as well as in (ii) the cSI-I gavage mixture, dams and their offspring. **b,c,** Principal component analysis of MAGs in feces of dams ordinated by their $\log_{10}$ absolute abundance and colored by bacterial consortium (**b**) or timepoint (**c**). The composition of community membership differs significantly between cSI-I and cSI-N dams (**b,** P=0.01, PERMANOVA) but is stable after the first week of colonization (**c**, P=0.09, PERMANOVA weeks 4 through 17). Each small point represents a fecal sample from one animal. Larger filled circles are the centroids for the ellipses shown, which denote the 95% confidence interval. **d,** MAGs whose absolute abundance differs significantly between P37 (left) and P14 (right) cSI-I and cSI-N mice are indicated (•, P-adj < 0.05, ••, P-adj < 0.01, •••, P-adj < 0.001, FDR-corrected two-sided Wilcoxon rank-sum). All 51 MAGs are included in the heatmap. n=11 and n=25 P14 and P37 cSI-N offspring, respectively; n=19 and n=21 P14 and P37 cSI-I offspring; 3-8 pups/litter.

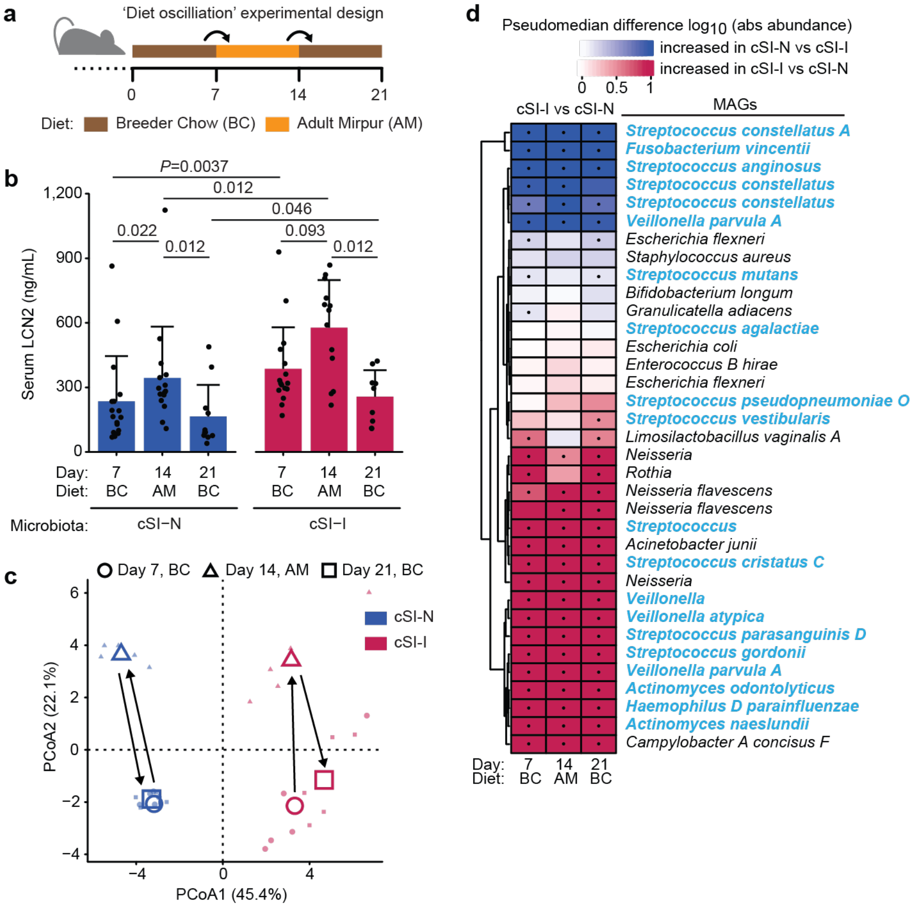

Supplementary Fig. 3. Diet oscillation experiment. a, Design of experiment to test dietary effects on systemic inflammation induced by cSI-I consortium. **b,** Serum levels of LCN2 after mice were fed breeder chow for one week (day 7, n=16 cSI-I mice, n=17 cSI-N), Adult Mirpur the following seven days (day 14, n=13 cSI-I mice, n=15 cSI-N), and a final week of breeder chow (day 21, n=9 cSI-I mice, n=11 cSI-N) (two-sided Wilcoxon rank-sum tests, bars denote mean ± s.d.). **c,** Principal component analysis of MAGs in feces of mice during the transition from breeder chow (the larger shape denotes the centroid of points for each diet and timepoint, circle) to Adult Mirpur (triangle) and back to breeder chow (square). **d,** Detectable MAGs ($\log_{10}$ count > 0) in fecal samples are included in the heatmap; MAGs that match 'core taxa' described in children with EED are highlighted in blue, as in **Fig. 1b**, MAGs whose absolute abundance differs significantly between cSI-I and cSI-N mice are indicated (•, P-adj < 0.05, FDR-corrected two-sided Wilcoxon rank-sum).

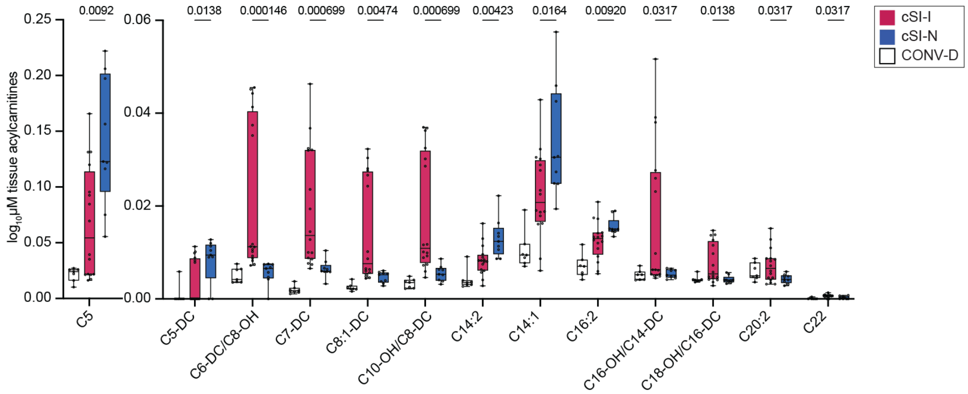

Supplementary Fig. 4. P37 intestinal acylcarnitine levels. Concentration of acylcarnitines in small intestinal tissue of P37 offspring. Acylcarnitines included in this figure exhibited a statistically significant difference in levels between cSI-I and cSI-N (unadjusted *P*-value < 0.05 for all 66 acylcarnitines quantified, multiple Mann-Whitney tests). *q*-values shown are FDR-corrected (Benjamini, Krieger, Yekutieli two-stage step up) Mann-Whitney tests comparing levels of the 13 acylcarnitines included in the figure between cSI-I and cSI-N (n=7 CONV-D animals, n=9 cSI-N, n=16 cSI-I; boxplots denote median, quartiles, minima and maxima).

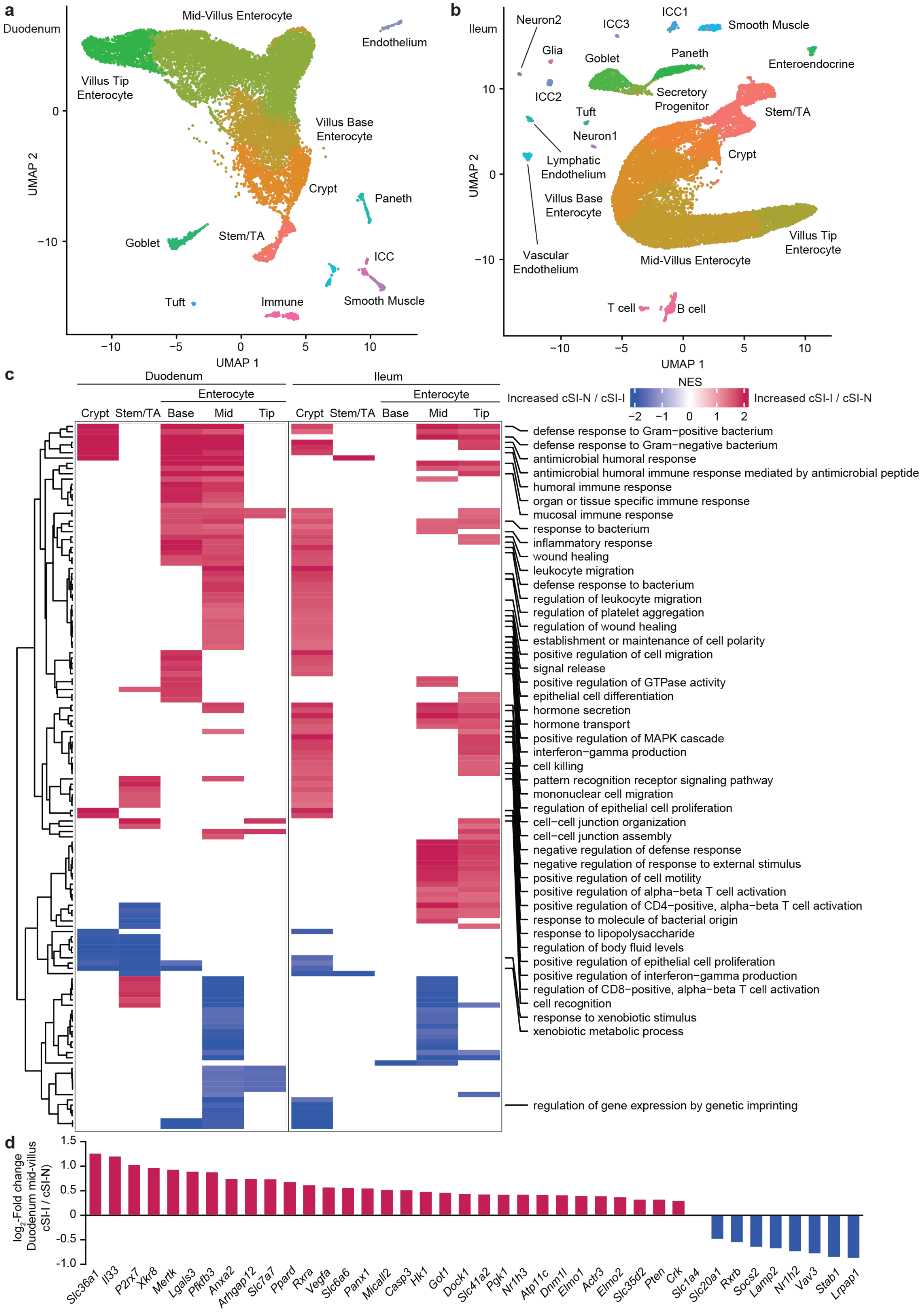

Supplementary Fig. 5. snRNA-Seq gene expression in the duodenum and ileum of P37 cSI-I versus cSI-N animals. **a,b,** Uniform manifold and projection (UMAP) plot of single nuclei isolated from the duodenum (**a**), and ileum (**b**) of P37 offspring (n=3 mice/group). **c,** Normalized enrichment scores (NES) for all GO categories (rows) that were significantly

enriched ($q < 0.05$, GSEA) in at least two of the indicated cell populations (columns) in either the duodenum (left) or ileum (right). Immune-related GO terms are labeled. If a GO category was non-significant, it was assigned an NES value of 0. **d,** Genes that were significantly differentially expressed and comprised the 'leading edge' of the efferocytosis gene set in duodenal mid-villus enterocytes (cSI-I versus cSI-N; P-adj < 0.05, GSEA). See **Supplementary Table 3c** for annotations.

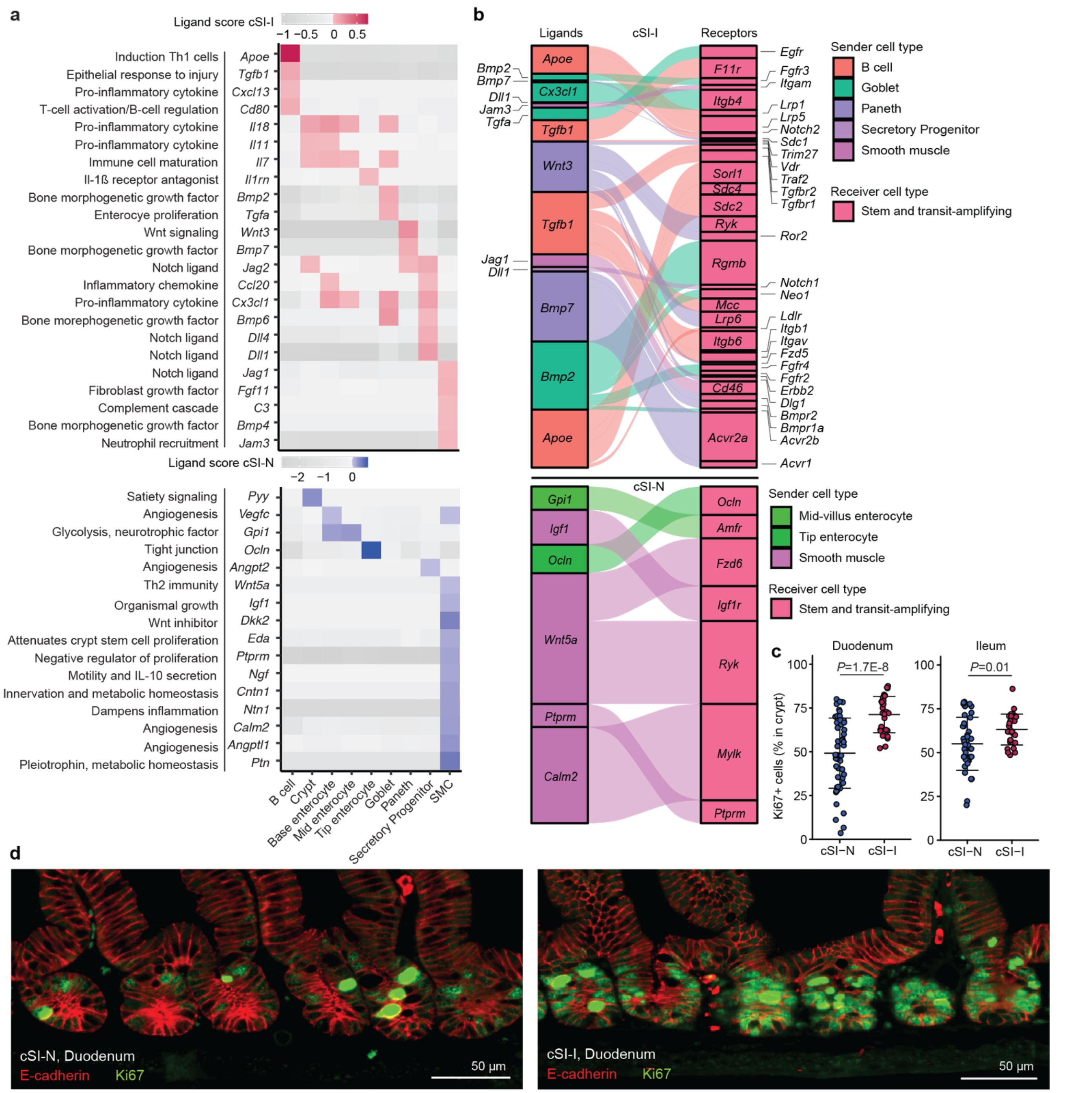

Supplementary Fig. 6. Increased proliferative signaling in the small intestine of P37 offspring of cSI-I dams. a, Intercellular signaling from cell populations (columns) to stem/transit amplifying (TA) cells in the ileum. A subset of ligands identified by *NicheNet* are shown that were differentially expressed between cSI-I and cSI-N animals. Annotations for these ligands (left) are based on literature findings. Ligands that were more highly expressed in cSI-I mice are shown in red (top of panel); those more highly expressed in cSI-N animals are shown in blue (bottom of panel). Their paired receptors are shown in (**b**). See **Supplementary Table 3c** for full *NicheNet* results. **b,** *NicheNet* analysis of the expression of receptors (right) in stem and transit-amplifying (TA) cells for ligands (left) expressed by various cell types in the ileum of P37 animals. Upper panel – ligands expressed more highly in cSI-I animals; lower panel – ligands expressed more highly in cSI-N animals. Boxes are sized proportionally to the weighted ligand/receptor score. **c,** Percent of all cells in a crypt that were Ki67^{+} in the duodenum (left) or ileum (right) (n=3 cSI-I offspring, n=5 cSI-N; each dot represents a single crypt-villus unit; 10 crypts analyzed per intestinal segment per mouse). *P*-values were determined by Tukey's post-hoc tests; mean values ± s.d. are shown. **d,** Representative sections of crypts from the duodenum of cSI-N (left) and cSI-I (right) P37 mice. Sections were stained with antibodies to E-cadherin (red) and Ki67 (green). Scale bar, 50 µm.

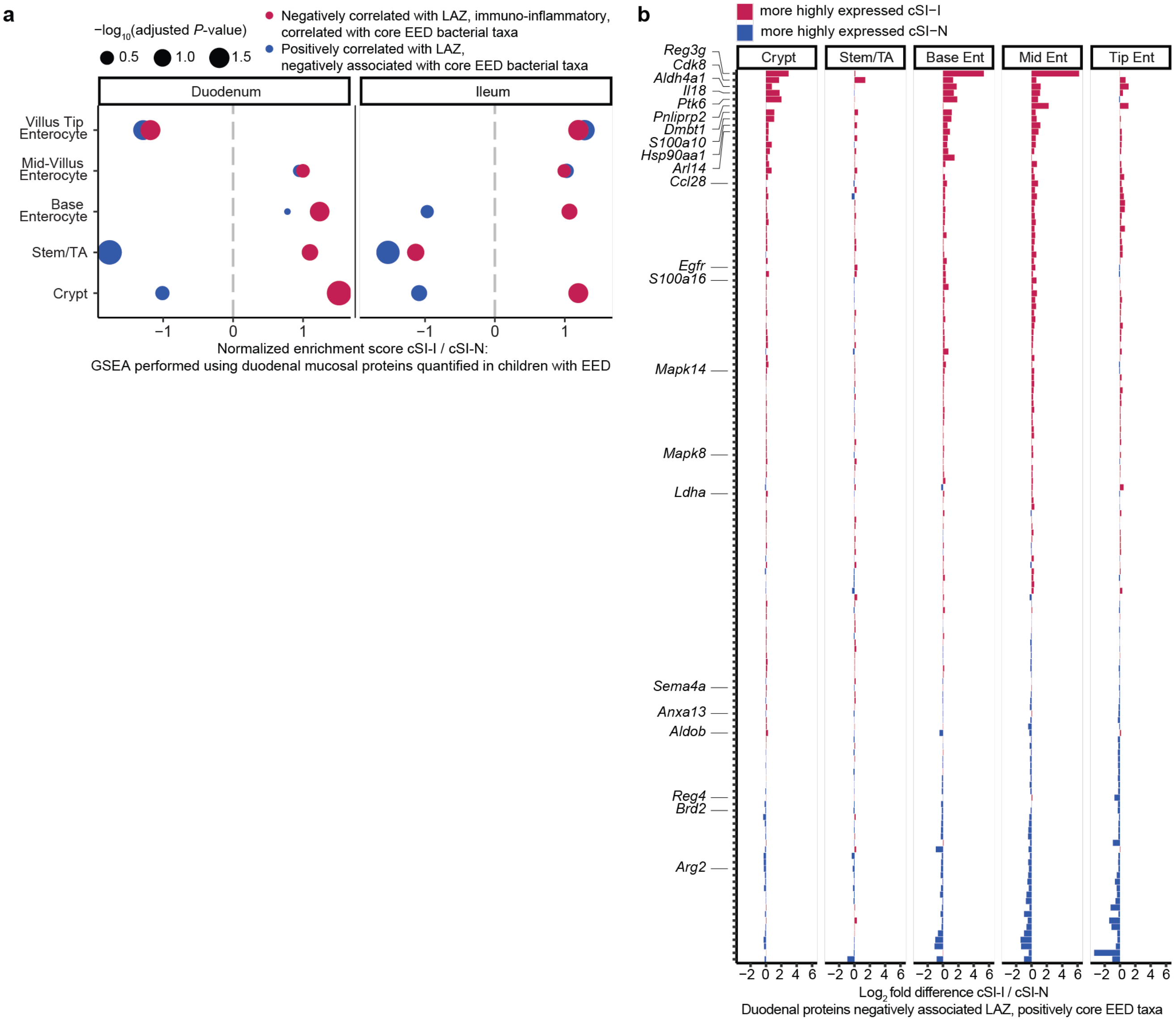

Supplementary Fig. 7. Expression of genes encoding orthologs of proteins quantified in the duodenal mucosa of children with EED in P37 animals. a, Gene set enrichment analysis (GSEA) along the crypt-villus axis, focusing on genes encoding duodenal mucosal proteins whose levels were quantified in children with EED in the BEED study. **b,** Expression of transcripts (rows) in cell populations (columns) in the duodenum of P37 animals that match proteins, quantified in biopsies of the duodenal mucosa of Bangladeshi children with EED, that were (i) negatively correlated with their LAZ and (ii) positively correlated with the absolute abundances of 'core' EED bacterial taxa; the abundances of these taxa were positively correlated with the degree of stunting. See **Supplementary Table 3c** for a list of the mouse homologs of LAZ-associated human proteins.

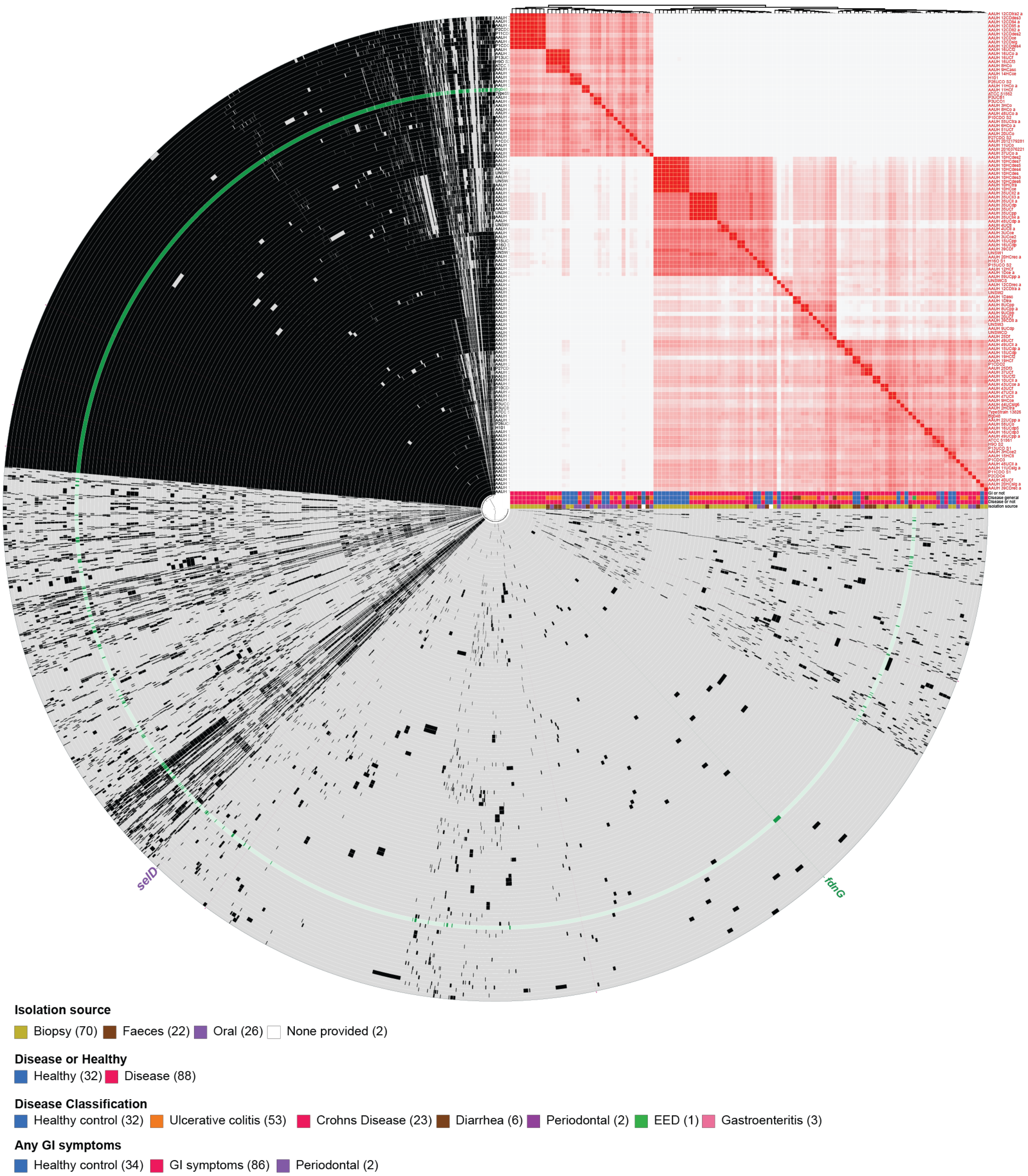

Supplementary Fig. 8. Comparative genomic analysis of *C. concisus* isolates. 120 whole *C. concisus* genomes are included in the circle phylogram that were isolated from intestinal biopsies, feces, or saliva of healthy or diseased individuals. Genes that are present are shown in black; if genes are absent, in gray. The Bangladeshi isolate from children with EED (Bg048) is highlighted in green. The nitrate-inducible formate dehydrogenase (*fdnG*) unique to this Bangladeshi isolate and a selenate di-kinase (*selD*) unique to the Bangladeshi isolate and one other genome are highlighted. Heatmap shows average nucleotide identity (ANI) comparisons between all isolates.

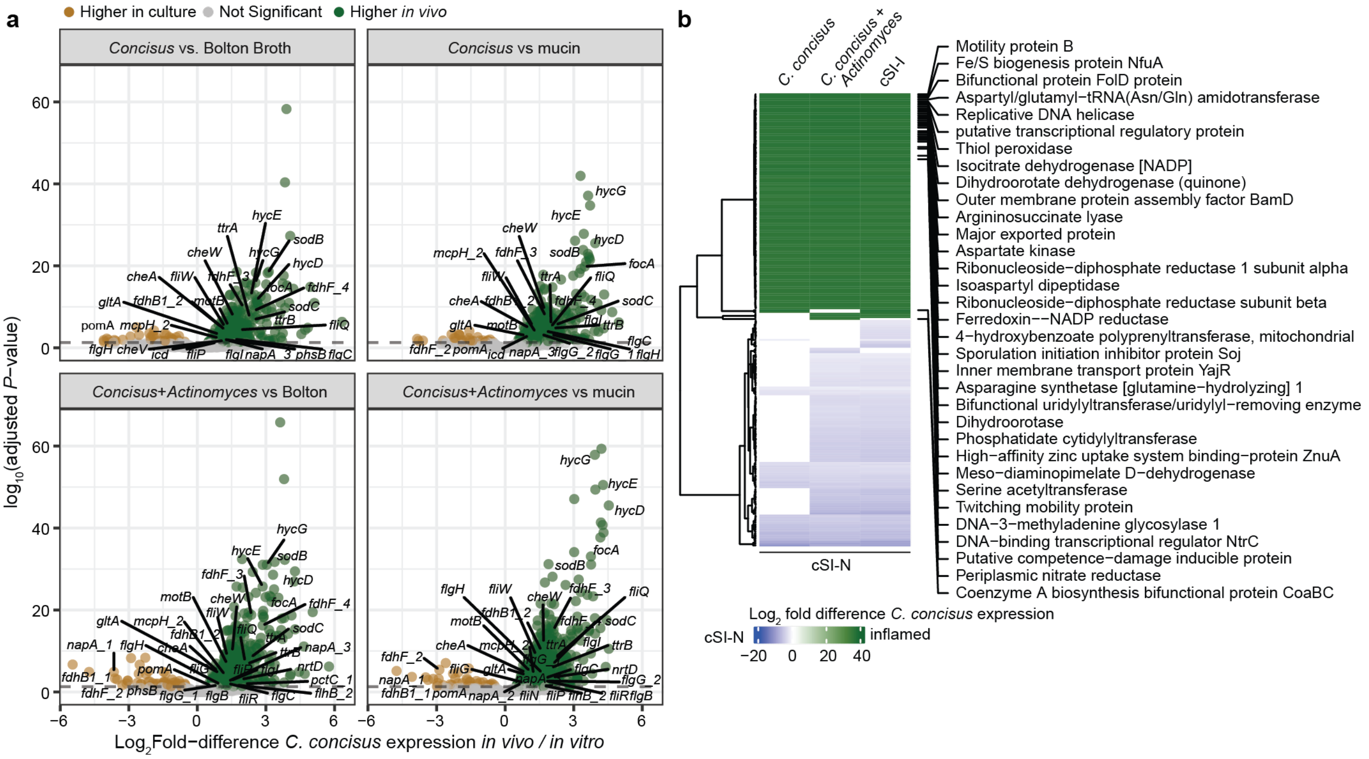

Supplementary Fig. 9. *C. concisus* gene expression during inflammatory and non-inflammatory conditions. a, *C. concisus* gene expression in the gut (*C. concisus*, *C. concisus+Actinomyces*) from the isolate 'add-in' experiment (**Fig. 3**) compared to growth in rich medium (Bolton broth, or Bolton broth supplemented with 1% mucin). Green: P-adj < 0.05 and higher expression *in vivo*, brown: P-adj < 0.05 and higher expression *in vitro* (*DESeq2* Wald test, n=4 biological replicates/condition). Genes involved in formate metabolism and anaerobic respiration are labelled. **b,** *C. concisus* genes that were significantly differentially expressed (*DESeq2*, Wald test) in the cecal contents of cSI-I and *C. concisus*-gavaged animals, ± *Actinomyces* ('inflamed') versus sham-gavaged cSI-N controls. The products of the most significantly differentially expressed genes across all three pairwise comparisons are labeled. n=5 cSI-N/*C. concisus* mice, n=10 cSI-N and cSI-I sham-gavaged controls, n=11 cSI-N/*C. concisus+Actinomyces* mice from two independent experiments.

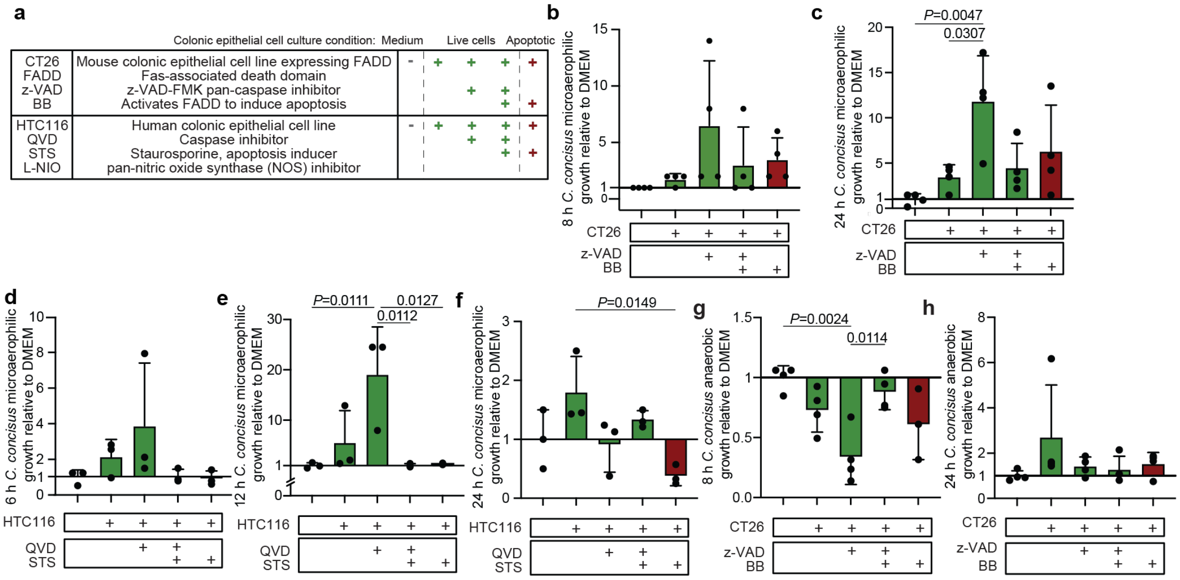

Supplementary Fig. 10. Mouse and human epithelial cell substrates boost *C. concisus* growth *in vitro*. a, Abbreviations of cell lines and treatments used to collect conditioned medium from mouse and human colonic epithelial cells that were live or apoptotic. QVD, Quinoline-Val-Asp-Difluorophenoxymethylketone (caspase inhibitor); L-NIO, N^5-(1-Iminoethyl)-L-ornithine, dihydrochloride (nitric oxide synthase inhibitor). Conditioned medium acquired from live epithelial cells is colored in green; apoptotic cells, in red. **b,c,** Growth of *C. concisus* after 8 hours (**b,** one-way ANOVA $F_{(4,15)}$=1.860, *P*=0.1700) or 24 hours (**c,** one-way ANOVA $F_{(4,15)}$=5.268, *P*=0.0075) in conditioned medium collected from CT26 mouse colonic epithelial cells that were live (CT26:FADD, z-VAD, z-VAD+BB) or were treated with an inducer of apoptosis (BB). Growth is presented as a ratio to growth in cell culture medium (n=4 biological replicates/condition). **d-f,** Growth of *C. concisus* after 6 (**d,** $F_{(4,10)}$=1.659, *P*=0.2350), 12 (**e,** $F_{(4,10)}$=6.734, *P*=0.0068), or 24 (**f,** $F_{(4,10)}$=4.570, *P*=0.0234), hours in spent medium collected from HTC116 human colonic epithelial cells that were live (QVD, QVD+STS), or that had been treated with an inducer of apoptosis (STS) relative to growth in cell culture medium (n=3 biological replicates/condition, growth is presented as a ratio to growth in cell culture medium. **g,h,** Anaerobic growth of *C. concisus* after 8 (**g,** one-way ANOVA, $F_{(4,14)}$=6.694, *P*=0.0031, n=4 biological replicates/condition except for apoptotic cell supernatants where n=3) or 24 hours (**h,** one-way ANOVA, $F_{(4,15)}$=1.362, *P*=0.2936, n=4 biological replicates/treatment condition) in conditioned medium from live CT26 mouse colonic epithelial cells (CT26:FADD, z-VAD, z-VAD+BB) or from cells treated with an inducer of apoptosis (BB) relative to growth in tissue culture medium. For **b-h**, bars denote mean ± s.d. *P*-values determined using Tukey's multiple comparisons.

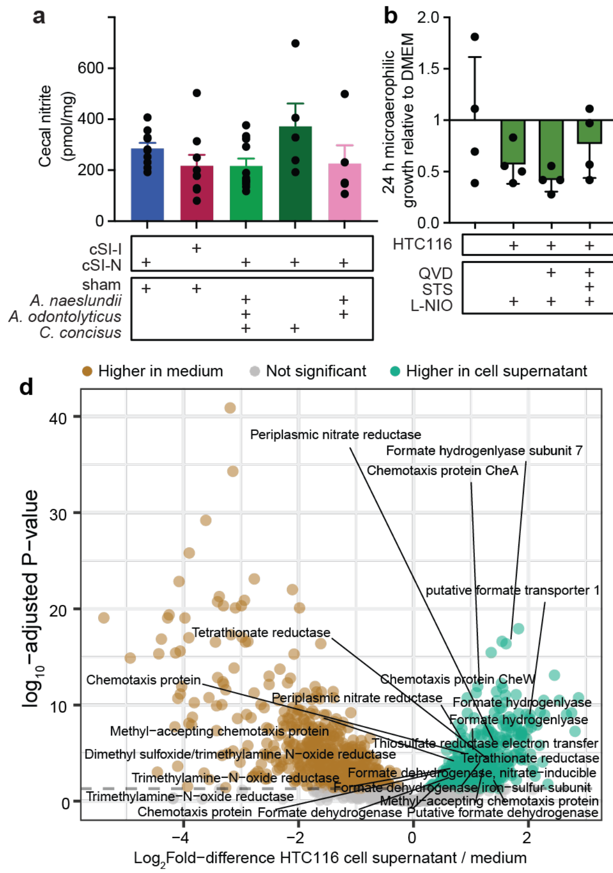

Supplementary Fig. 11. Inhibition of NOS activity in human colonic epithelial cells abrogates benefit to *C. concisus* growth from products of colonic epithelial cells. a, Levels of nitrite in cecal contents 9 days post-isolate gavage (mice combined from two independent experiments, resulting in n=10 cSI-I and cSI-N, n=5 cSI-N/*C. concisus* and cSI-N/*Actinomyces*, n=11 cSI-N/*C. concisus*+*Actinomyces* mice (bars denote mean ± s.e.m., one-way ANOVA, $F_{(4,35)}$=1.865, *P*=0.1385). **b,** Microaerophilic growth of *C. concisus* after 24 hours growth in conditioned medium from HTC116 human colonic epithelial cells acquired from live cells (QVD, QVD+STS) or cells treated with an inducer of apoptosis (STS). All cells were treated with an inhibitor of nitric oxide synthase (L-NIO). Growth shown as a ratio to growth in cell culture medium alone (one-way ANOVA, $F_{(4,15)}$=1.765, *P*=0.1885). **c,** Anaerobic growth of *C. concisus* after 24 hours in conditioned medium from HTC116 cells collected from live cells (QVD, QVD+STS) or cells that had been treated with an inducer of apoptosis (STS). All cells were treated with L-NIO (one-way ANOVA, $F_{(4,18)}$=0.7876, *P*=0.5482). For **b,c,** bars denote mean ± s.d., n=4 biological replicates/condition except for L-NIO/QVD/STS-treated HTC116 cells grown anaerobically, where n=7. **d,** *C. concisus* genes that were significantly differentially expressed when cultured in conditioned medium collected from HTC116 cells (positive log_2-fold change) compared to medium alone (negative log_2-fold change, *DESeq2* Wald test, n=4 biological replicates/condition).

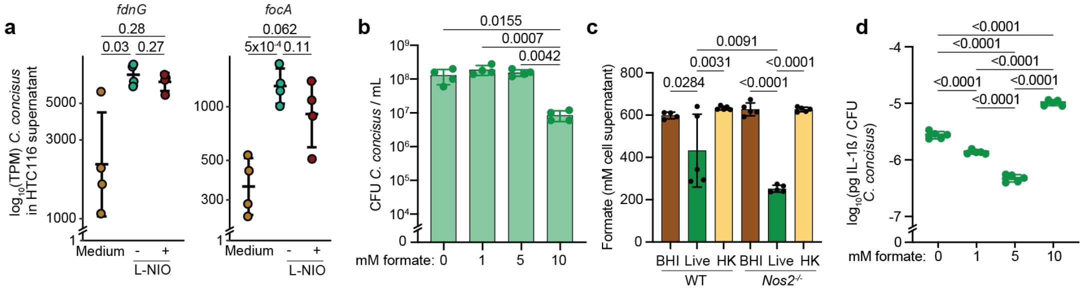

Supplementary Fig. 12. *C. concisus* pre-treatment with formate increases production of IL-1ß. a, Expression of the *C. concisus* nitrate-inducible formate dehydrogenase (*fdnG*) and formate transporter (*focA*) when cultured in supernatants collected from HTC116 cells that were or were not treated with the NOS inhibitor L-NIO or cell culture medium alone (statistics shown are unadjusted *P* values from the *DESeq2* Wald test, transcripts per million, n=4 biological replicates/condition). **b,** *C. concisus* cultured in Bolton broth supplemented with various levels of formate; growth was quantified after 24 hours (one-way ANOVA, $F_{(3,12)}$=11.17, *P*=0.0009, n=4 biological replicates/condition). **c,** Formate was quantified in supernatants from bone marrow cells incubated for 48 hours with bacterial culture medium (BHI), fresh *C. concisus* culture (Live), or heat-killed *C. concisus* culture (HK, one-way ANOVA, $F_{(5,23)}$=22.11, *P*<0.0001). **d,** IL-1ß quantified in supernatants collected from bone marrow cells that had been incubated for 24 hours with *C. concisus* pre-treated with various concentrations of formate (one-way ANOVA, $F_{(3,16)}$=527.4, *P*<0.0001, n=5 biological replicates/condition). For all panels, bars denote mean ± s.d. *P*-values shown are the result of Tukey's multiple comparisons.

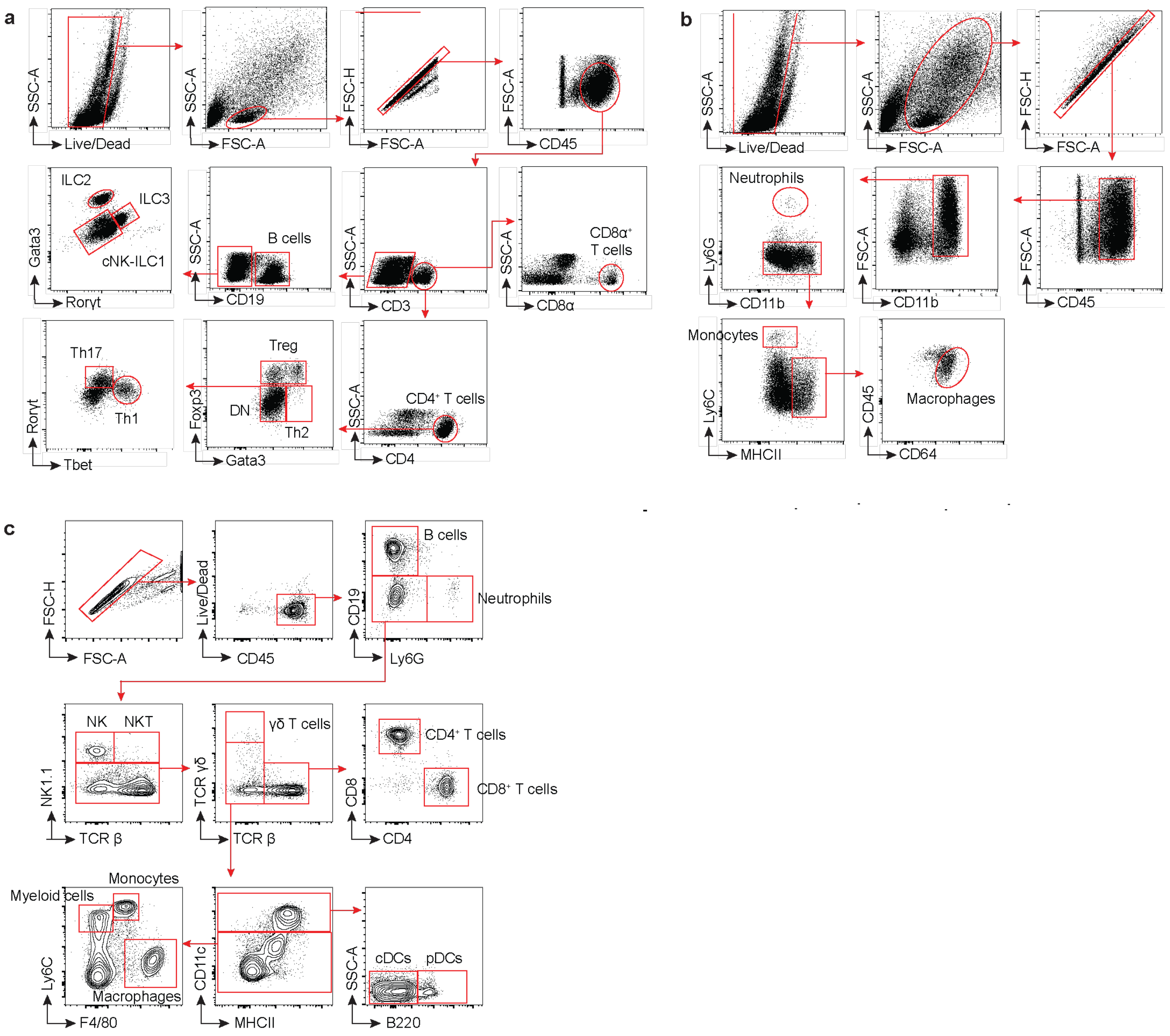

Supplementary Fig. 13. Gating strategies for flow cytometry. a,b, Representative gating strategy for quantifying lymphoid (**a**) and myeloid (**b**) immune cells in the intestinal lamina propria. **c,** Representative gating strategies for meninges and spleen. See **Supplementary Table 7** for antibodies used.

References

1. Chen, R. Y. *et al.* Duodenal microbiota in stunted undernourished children with enteropathy. *N. Engl. J. Med.* **383**, 321–333 (2020).
2. Semba, R. D. *et al.* Environmental enteric dysfunction is associated with carnitine deficiency and altered fatty acid oxidation. *EBioMedicine* **17**, 57–66 (2017).
3. Lemons, J. M. S. *et al.* Enterobacteriaceae growth promotion by intestinal acylcarnitines, a biomarker of dysbiosis in inflammatory bowel disease. *Cell. Mol. Gastroenterol. Hepatol.* **17**, 131–148 (2024).
4. Dambrova, M. *et al.* Acylcarnitines: Nomenclature, biomarkers, therapeutic potential, drug targets, and clinical trials. *Pharmacol. Rev.* **74**, 506–551 (2022).
5. Rutkowsky, J. M. *et al.* Acylcarnitines activate proinflammatory signaling pathways. *Am. J. Physiol. Endocrinol. Metab.* **306**, E1378–E1387 (2014).
6. Liu, T. C. *et al.* A novel histological index for evaluation of environmental enteric dysfunction identifies geographic-specific features of enteropathy among children with suboptimal growth. *PLoS Negl. Trop. Dis.* **14**, 1–21 (2020).
7. Mulenga, C. *et al.* Epithelial abnormalities in the small intestine of Zambian children with stunting. *Front. Med.* **9**, 849677 (2022).
8. Lee, C. S. *et al.* Boosting apoptotic cell clearance by colonic epithelial cells attenuates inflammation in vivo. *Immunity* **44**, 807–820 (2016).
9. Shankman, L. S. *et al.* Efferocytosis by Paneth cells within the intestine. *Curr. Biol.* **31**, 2469-2476.e5 (2021).
10. Mehrotra, P. & Ravichandran, K. S. Drugging the efferocytosis process: concepts and opportunities. *Nat. Rev. Drug Discov.* **21**, 601–620 (2022).
11. Anderson, C. J. *et al.* Microbes exploit death-induced nutrient release by gut epithelial cells. *Nature* **596**, 262–267 (2021).
12. Morioka, S. *et al.* Efferocytosis induces a novel SLC program to promote glucose uptake and lactate release. *Nature* **563**, 714–718 (2018).
13. Perry, J. S. A. *et al.* Interpreting an apoptotic corpse as anti-inflammatory involves a chloride sensing pathway. *Nature Cell Biol.* **21**, 1532–1543 (2019).
14. Maschalidi, S. *et al.* Targeting SLC7A11 improves efferocytosis by dendritic cells and wound healing in diabetes. *Nature* **606**, 776–784 (2022).
15. Browaeys, R., Saelens, W. & Saeys, Y. NicheNet: modeling intercellular communication by linking ligands to target genes. *Nat. Methods* **17**, 159–162 (2020).
16. Guilliams, M. *et al.* Spatial proteogenomics reveals distinct and evolutionarily conserved hepatic macrophage niches. *Cell* **185**, 379-396.e38 (2022).
17. Takahashi, T. & Shiraishi, A. Stem cell signaling pathways in the small intestine. *Int. J. Mol. Sci.* **21**, 2032 (2020).
18. Vanuytsel, T., Senger, S., Fasano, A. & Shea-Donohue, T. Major signaling pathways in intestinal stem cells. *Biochim. Biophys. Acta* **1830**, 2410–2426 (2013).
19. Yu, X. M. *et al.* Wnt5a inhibits hypoxia-induced pulmonary arterial smooth muscle cell proliferation by downregulation of β-catenin. *Am. J. Physiol. Lung Cell. Mol. Physiol.* **304**, L103–L111 (2013).
20. Nattramilarasu, P. K., Sá, F. D. L. de, Schulzke, J. D. & Bücker, R. Immune-mediated aggravation of the *Campylobacter concisus*-induced epithelial barrier dysfunction. *Int. J. Mol. Sci.* **22**, 2043 (2021).
21. Aagaard, M. E. Y., Kirk, K. F., Nielsen, H. L., Steffensen, R. & Nielsen, H. *Campylobacter concisus* from chronic inflammatory bowel diseases stimulates IL-8 production in HT-29 cells. *Gut Pathog.* **15**, 5 (2023).
22. Mahendran, V. *et al.* The prevalence and polymorphisms of zonula occluden toxin gene in multiple *Campylobacter concisus* strains isolated from saliva of patients with inflammatory bowel disease and controls. *PLoS ONE* **8**, e77525 (2013).
23. Liu, B., Zheng, D., Zhou, S., Chen, L. & Yang, J. VFDB 2022: a general classification scheme for bacterial virulence factors. *Nucleic Acids Res.* **50**, D912–D917 (2021).
24. Kirk, K. F. *et al.* Molecular epidemiology and comparative genomics of *Campylobacter concisus* strains from saliva, faeces and gut mucosal biopsies in inflammatory bowel disease. *Sci. Rep.* **8**, 1902 (2018).
25. Liu, F. *et al.* Analysis of complete *Campylobacter concisus* genomes identifies genomospecies features, secretion systems and novel plasmids and their association with severe ulcerative colitis. *Microb. Genom.* **6**, mgen000457 (2020).
26. Khangulov, S. V., Gladyshev, V. N., Dismukes, G. C. & Stadtman, T. C. Selenium-containing formate dehydrogenase H from *Escherichia coli*: A molybdopterin enzyme that catalyzes formate oxidation without oxygen transfer. *Biochemistry* **37**, 3518–3528 (1998).

27. Yeow, M. *et al.* Analyses of energy metabolism and stress defense provide insights into *Campylobacter concisus* growth and pathogenicity. *Gut Pathog.* **12**, 13 (2020).
28. Litvak, Y., Byndloss, M. X. & Bäumler, A. J. Colonocyte metabolism shapes the gut microbiota. *Science* **362**, eaat9076 (2018).
29. Rivera-Chávez, F. & Bäumler, A. J. The pyromaniac inside you: *Salmonella* metabolism in the host gut. *Annu. Rev. Microbiol.* **69**, 31–48 (2015).
30. Rivera-Chávez, F. *et al.* Energy taxis toward host-derived nitrate supports a *Salmonella* pathogenicity island 1-independent mechanism of invasion. *mBio* **7**, e00960-16 (2016).
31. Winter, S. E. *et al.* Host-derived nitrate boosts growth of *E. coli* in the inflamed gut. *Science* **339**, 708–711 (2013).
32. Faber, F. *et al.* Host-mediated sugar oxidation promotes post-antibiotic pathogen expansion. *Nature* **534**, 697–699 (2016).
33. McCall, T. B., Feelisch, M., Palmer, R. M. J. & Moncada, S. Identification of N-iminoethyl-l-ornithine as an irreversible inhibitor of nitric oxide synthase in phagocytic cells. *Br. J. Pharmacol.* **102**, 234–238 (1991).
34. Huang, Y., Suyemoto, M., Garner, C. D., Cicconi, K. M. & Altier, C. Formate acts as a diffusible signal to induce Salmonella invasion. *J. Bacteriol.* **190**, 4233–4241 (2008).
35. Wang, K.-C., Lerche, M. H., Ardenkjær-Larsen, J. H. & Jensen, P. R. Formate metabolism in *Shigella flexneri* and its effect on HeLa cells at different stages during the infectious process. *Microbiol. Spectr.* **11**, e00631-22 (2023).
36. Kassem, I. I., Candelero-Rueda, R. A., Esseili, K. A. & Rajashekara, G. Formate simultaneously reduces oxidase activity and enhances respiration in *Campylobacter jejuni*. *Sci. Rep.* **7**, 40117 (2017).
37. Rogawski, E. T. *et al.* Use of quantitative molecular diagnostic methods to investigate the effect of enteropathogen infections on linear growth in children in low-resource settings: longitudinal analysis of results from the MAL-ED cohort study. *Lancet Glob. Health* **6**, e1319–e1328 (2018).
38. Vonaesch, P. *et al.* Stunted children display ectopic small intestinal colonization by oral bacteria, which cause lipid malabsorption in experimental models. *Proc. Natl. Acad. Sci. USA* **119**, e2209589119 (2022).
39. Vonaesch, P. *et al.* Stunted childhood growth is associated with decompartmentalization of the gastrointestinal tract and overgrowth of oropharyngeal taxa. *Proc. Natl. Acad. Sci. USA* **115**, E8489–E8498 (2018).
40. Dinh, D. M. *et al.* Longitudinal analysis of the intestinal microbiota in persistently stunted young children in south India. *PLoS ONE* **11**, e0155405 (2016).
41. Zhang, L. *et al.* Isolation and detection of *Campylobacter concisus* from saliva of healthy individuals and patients with inflammatory bowel disease. *J. Clin. Microbiol.* **48**, 2965–2967 (2010).
42. Liu, F., Ma, R., Wang, Y. & Zhang, L. The clinical importance of *Campylobacter concisus* and other human hosted *Campylobacter* species. *Front. Cell. Infect. Microbiol.* **8**, 243 (2018).
43. Kirk, K. F., Nielsen, H. L., Thorlacius-Ussing, O. & Nielsen, H. Optimized cultivation of *Campylobacter concisus* from gut mucosal biopsies in inflammatory bowel disease. *Gut Pathog.* **8**, 27 (2016).
44. Chang, H.-W. *et al. Prevotella copri* and microbiota members mediate the beneficial effects of a therapeutic food for malnutrition. *Nat. Microbiol.* **9**, 922–937 (2024).
45. An, J. *et al.* Hepatic expression of malonyl-CoA decarboxylase reverses muscle, liver and whole-animal insulin resistance. *Nat. Med.* **10**, 268–274 (2004).
46. Seemann, T. Prokka: Rapid prokaryotic genome annotation. *Bioinformatics* **30**, 2068–2069 (2014).
47. Kanehisa, M., Sato, Y. & Morishima, K. BlastKOALA and GhostKOALA: KEGG tools for functional characterization of genome and metagenome sequences. *J. Mol. Biol.***428**, 726–731 (2016).
48. Overbeek, R. *et al.* The subsystems approach to genome annotation and its use in the project to annotate 1000 genomes. *Nucleic Acids Res.* **33**, 5691–5702 (2005).
49. Aziz, R. K. *et al.* SEED servers: High-performance access to the SEED genomes, annotations, and metabolic models. *PLoS ONE* **7**, e48053 (2012).
50. Rodionov, D. A. *et al.* Micronutrient requirements and sharing capabilities of the human gut microbiome. *Frontiers in Microbiology* **10**, 1316 (2019).
51. Ashniev, G. A., Petrov, S. N., Iablokov, S. N. & Rodionov, D. A. Genomics-based reconstruction and predictive profiling of amino acid biosynthesis in the human gut microbiome. *Microorganisms* **10**, 740 (2022).
52. Frolova, M. S., Suvorova, I. A., Iablokov, S. N., Petrov, S. N. & Rodionov, D. A. Genomic reconstruction of short-chain fatty acid production by the human gut microbiota. *Front. Mol. Biosci.* **9**, 949563 (2022).
53. Hibberd, M. C. *et al.* Bioactive glycans in a microbiome-directed food for malnourished children. *Nature* **625**, 157-165 (2023).
54. Fleming, S. J., Marioni, J. C. & Babadi, M. CellBender remove-background: A deep generative model for unsupervised removal of background noise from scRNA-seq datasets. *bioRxiv* (2019) doi:10.1101/791699.

55. Hao, Y. *et al.* Integrated analysis of multimodal single-cell data. *Cell* **184**, 3573–3587 (2021).
56. Hafemeister, C. & Satija, R. Normalization and variance stabilization of single-cell RNA-seq data using regularized negative binomial regression. *Genome Biol.* **20**, 296 (2019).
57. Choudhary, S. & Satija, R. Comparison and evaluation of statistical error models for scRNA-seq. *Genome Biol.* **23**, 27 (2022).
58. McGinnis, C. S., Murrow, L. M. & Gartner, Z. J. DoubletFinder: Doublet detection in single-cell RNA-sequencing data using artificial nearest neighbors. *Cell Syst.* **8**, 329-337.e4 (2019).
59. Moor, A. E. *et al.* Spatial reconstruction of single enterocytes uncovers broad zonation along the intestinal villus axis. *Cell* **175**, 1156–1167 (2018).
60. Love, M. I., Huber, W. & Anders, S. Moderated estimation of fold change and dispersion for RNA-seq data with DESeq2. *Genome Biol.* **15**, 550 (2014).
61. Yu, G., Wang, L. G., Han, Y. & He, Q. Y. ClusterProfiler: An R package for comparing biological themes among gene clusters. *OMICS* **16**, 284–287 (2012).
62. Eren, A. M. *et al.* Community-led, integrated, reproducible multi-omics with anvi'o. *Nat. Microbiol.* **6**, 3–6 (2021).
63. Shaiber, A. *et al.* Functional and genetic markers of niche partitioning among enigmatic members of the human oral microbiome. *Genome Biol.* **21**, 292 (2020).
64. Delmont, T. O. & Eren, A. M. Linking pangenomes and metagenomes: the Prochlorococcus metapangenome. *PeerJ* **6**, e4320 (2018).
