# Supplementary material for: Enteropathy produced in mice by intergenerational transmission of small intestinal microbiota from undernourished children
Source: Nat Microbiol. 2026 Jun 16;11(7):1967–81. doi: 10.1038/s41564-026-02394-4 (PMC13323066; doi:10.1038/s41564-026-02394-4)
Supplement: Supplementary file 2 — Reporting Summary [file 41564_2026_2394_MOESM2_ESM.pdf]

nature portfolio

Corresponding author(s): Jeffrey I. Gordon

Last updated by author(s): Mar 31, 2026

Reporting Summary

Nature Portfolio wishes to improve the reproducibility of the work that we publish. This form provides structure for consistency and transparency in reporting. For further information on Nature Portfolio policies, see our Editorial Policies and the Editorial Policy Checklist.

Statistics

For all statistical analyses, confirm that the following items are present in the figure legend, table legend, main text, or Methods section.

n/a	Confirmed	
☐	☒	The exact sample size (*n*) for each experimental group/condition, given as a discrete number and unit of measurement
☐	☒	A statement on whether measurements were taken from distinct samples or whether the same sample was measured repeatedly
☐	☒	The statistical test(s) used AND whether they are one- or two-sided<br>*Only common tests should be described solely by name; describe more complex techniques in the Methods section.*
☐	☒	A description of all covariates tested
☐	☒	A description of any assumptions or corrections, such as tests of normality and adjustment for multiple comparisons
☐	☒	A full description of the statistical parameters including central tendency (e.g. means) or other basic estimates (e.g. regression coefficient) AND variation (e.g. standard deviation) or associated estimates of uncertainty (e.g. confidence intervals)
☐	☒	For null hypothesis testing, the test statistic (e.g. *F*, *t*, *r*) with confidence intervals, effect sizes, degrees of freedom and *P* value noted<br>*Give P values as exact values whenever suitable.*
☒	☐	For Bayesian analysis, information on the choice of priors and Markov chain Monte Carlo settings
☒	☐	For hierarchical and complex designs, identification of the appropriate level for tests and full reporting of outcomes
☒	☐	Estimates of effect sizes (e.g. Cohen's *d*, Pearson's *r*), indicating how they were calculated

Our web collection on statistics for biologists contains articles on many of the points above.

Software and code

Policy information about availability of computer code

Data collection	QuPath v0.2.3 was used for histomorphometric analysis of the small intestine.
Data analysis	The following software packages were used to analyze the data in this study: metaFlye (v2.8.1), checkm (v1.0.7), CoverM (v0.6.1), MaxBin (v2.2.7), MAGpurify (v2.1.2), dRep (v2.3.2), quast (v4.5), the Genome Taxonomy Database Toolkit (v1.5.1), iTOL (v6), BV-PRC Phylogenetic Tree Building Service within BV-BRC (3.32.13.a), trimgalore (v0.6.1), bowtie2 (v2.3.5), Flye (v2.9), prokka (v1.14), FastQC (v0.11.7), trimgalore (v0.6.1), kallisto (v0.46.2), tximport (v1.22.0), biomaRt (v2.50.3), limma-voom (v3.50.3), DESeq2(v1.44.0), clusterProfiler (v4.2.2), CellRanger (v5.0), CellBender (0.2), Seurat (v4.0), DoubletFinder (v2.0), SingleCellExperiment (v1.16.0), nichenetr (v1.1.0), and fgsea (v1.27.0). Parameters used and implementation of these software packages are described in the Methods section. rstatix (0.7.2), ggplot2 (v3.4.3), and ComplexHeatmap (2.10.0) were used for additional statistics and visualization in R (v4.0.4 and 4.1.2).

For manuscripts utilizing custom algorithms or software that are central to the research but not yet described in published literature, software must be made available to editors and reviewers. We strongly encourage code deposition in a community repository (e.g. GitHub). See the Nature Portfolio guidelines for submitting code & software for further information.

Data

Policy information about availability of data

All manuscripts must include a data availability statement. This statement should provide the following information, where applicable:

- Accession codes, unique identifiers, or web links for publicly available datasets
- A description of any restrictions on data availability
- For clinical datasets or third party data, please ensure that the statement adheres to our policy

Datasets generated by (i) shotgun sequencing of a) DNA isolated from the intestinal contents of gnotobiotic mice (including resulting MAGs) and b) individual cultured bacterial strains, (ii) snRNA-Seq and bulk RNA-Seq of intestinal tissue and (iii) microbial RNA-Seq of cecal contents harvested from gnotobiotic mice have been deposited at the European Nucleotide Archive (ENA; https://www.ebi.ac.uk/ena) under accession number PRJEB61647.

Research involving human participants, their data, or biological material

Policy information about studies with human participants or human data. See also policy information about sex, gender (identity/presentation), and sexual orientation and race, ethnicity and racism.

Reporting on sex and gender	Please refer to Chen, Kung et al., "Duodenal microbiota in stunted undernourished children with enteropathy", N Engl J Med 2020 (DOI: 10.1056/NEJMoa1916004) for information regarding population characteristics of the participants from which duodenal aspirates were obtained and bacterial strains used in this study were isolated.
Reporting on race, ethnicity, or other socially relevant groupings	Please refer to Chen, Kung et al., "Duodenal microbiota in stunted undernourished children with enteropathy", N Engl J Med 2020 (DOI: 10.1056/NEJMoa1916004) for information regarding population characteristics of the participants from which duodenal aspirates were obtained and bacterial strains used in this study were isolated.
Population characteristics	Please refer to Chen, Kung et al., "Duodenal microbiota in stunted undernourished children with enteropathy", N Engl J Med 2020 (DOI: 10.1056/NEJMoa1916004) for information regarding population characteristics of the participants from which duodenal aspirates were obtained and bacterial strains used in this study were isolated.
Recruitment	Please refer to Chen, Kung et al., "Duodenal microbiota in stunted undernourished children with enteropathy", N Engl J Med 2020 (DOI: 10.1056/NEJMoa1916004) for information regarding population characteristics of the participants from which duodenal aspirates were obtained and bacterial strains used in this study were isolated.
Ethics oversight	The study protocol was approved by the Ethical Review Committee at the International Center for Diarrheal Disease Research, Bangladesh (icddr,b).

Note that full information on the approval of the study protocol must also be provided in the manuscript.

Field-specific reporting

Please select the one below that is the best fit for your research. If you are not sure, read the appropriate sections before making your selection.

☒ Life sciences ☐ Behavioural & social sciences ☐ Ecological, evolutionary & environmental sciences

For a reference copy of the document with all sections, see nature.com/documents/nr-reporting-summary-flat.pdf

Life sciences study design

All studies must disclose on these points even when the disclosure is negative.

Sample size	The number of adult mice in each arm of the intergenerational transmission experiment was chosen to encourage successful breeding (in trios) and to enable statistical comparisons between each group of dams (n=4/group). The number of offspring collected in the intergenerational transmission experiment was determined by the number of pups born in each litter. For all post-weaning experiments, the number of animals per group used was 4-6, to enable statistical comparisons across groups.
Data exclusions	The only outliers identified and removed were from the nitric oxide quantification. They were identified using the ROUT method with the most stringent cut-off (Q=0.1%) implemented in Prism 10; all data are reported in Supplementary Data Table 2g.
Replication	The co-housing animal experiments were repeated twice independently; bacterial isolate add-in experiments were repeated three times. C. concisus growth assays and ex vivo stimulations were repeated 2-3 times.
Randomization	Dams were randomly assigned to each treatment group for the intergenerational transmission experiment. For all post-weaning experiments, animals were randomized to groups, optimizing for equal mean and standard deviation of starting body weight.
Blinding	Investigators were blinded with respect to experimental group for histomorphometric and immunohistochemical analyses and of the intestine. No other blinding was performed.

Reporting for specific materials, systems and methods

We require information from authors about some types of materials, experimental systems and methods used in many studies. Here, indicate whether each material, system or method listed is relevant to your study. If you are not sure if a list item applies to your research, read the appropriate section before selecting a response.

Materials & experimental systems

n/a	Involved in the study
☐	☒ Antibodies
☐	☒ Eukaryotic cell lines
☒	☐ Palaeontology and archaeology
☐	☒ Animals and other organisms
☒	☐ Clinical data
☒	☐ Dual use research of concern
☒	☐ Plants

Methods

n/a	Involved in the study
☒	☐ ChIP-seq
☐	☒ Flow cytometry
☒	☐ MRI-based neuroimaging

Antibodies

Antibodies used	See Supplementary Table 7 for the list of all antibodies used.
Validation	Vendors and catalog numbers of all antibodies used are provided, where individual validation information can be found.

Eukaryotic cell lines

Policy information about cell lines and Sex and Gender in Research

Cell line source(s)	Human HTC116 (catalogue number CCL-247) and mouse CT26 (catalogue number CRL-2638) cells were obtained from ATCC.
Authentication	Morpohology of cell lines was monitored via routine microscopic examination.
Mycoplasma contamination	All cell lines used in the laboratory are tested for mycoplasma contamination and tested negative.
Commonly misidentified lines (See ICLAC register)	Neither of the cell lines used in this study are on the ICLAC list of commonly misidentified lines.

Animals and other research organisms

Policy information about studies involving animals; ARRIVE guidelines recommended for reporting animal research, and Sex and Gender in Research

Laboratory animals	Germ-free C57Bl/6J (The Jackson Laboratory) mice and their pups were used. All experiments involving mice were performed using protocols approved by Washington University Animal Studies Committee (protocol 23-0271). Germ free and gnotobiotic mice were housed in plastic flexible film isolators (Class Biologically Clean Ltd., Madison, WI). Specific-pathogen-free C57Bl/6J (The Jackson Laboratory) and Nos2-/- (B6.129P2-Nos2tm1Lau/J, Jackson) mice were maintained in a cage rack system. All mice were housed at 23 C under a strict 12-hour light cycle (lights on at 0600h). Autoclaved paper 'shepherd shacks' were kept in each cage to facilitate the natural nesting behaviors and for environmental enrichment.
Wild animals	No wild animals were used.
Reporting on sex	Both female and male mice were used in this study. No significant differences were found between female and male adults or offspring animals in phenotypes assessed within a colonization condition.
Field-collected samples	No field-collected samples were used.
Ethics oversight	All gnotobiotic mouse experiments were performed according to IACUC (23-0271) and IBC (15436) protocols that were approved by the Washington University Animal Studies and Environmental Health and Safety Committee.

Note that full information on the approval of the study protocol must also be provided in the manuscript.

April 2023

Plants

Seed stocks: N/A

Novel plant genotypes: N/A

Authentication: N/A

Flow Cytometry

Plots

Confirm that:

☒ The axis labels state the marker and fluorochrome used (e.g. CD4-FITC).

☒ The axis scales are clearly visible. Include numbers along axes only for bottom left plot of group (a 'group' is an analysis of identical markers).

☒ All plots are contour plots with outliers or pseudocolor plots.

☒ A numerical value for number of cells or percentage (with statistics) is provided.

Methodology

Sample preparation

Intestinal tissue was digested and myeloid and lymphoid cells were collected according to methods previously described ([DOI: 10.1056/NEJMoa1916004]). Briefly, each subsegment was immediately flushed with cold PBS after dissection to remove luminal contents. Each subsegment was then opened lengthwise and gently agitated for 20 minutes at room temperature in Hanks Balanced Salt Solution (HBSS) supplemented with 15mM HEPES, 10% bovine calf serum (BCS) and 5mM EDTA. Each sample was vortexed and the suspended cells were collected; the remaining tissue fragments were subjected to a second round of gentle agitation and vortexing. The tissue remaining after the second collection was rinsed with cold 1X HBSS prior to digestion with Collagenase IV (Sigma) in complete RPMI-1640 for 40 minutes at 37 °C with gentle agitation. Digests were filtered through 100 um mesh strainer (Falcon) and subjected to density gradient centrifugation using 40% and 70% Percol solutions (GE Healthcare).

To dissect meninges, we removed the skin and muscle overlying the skull as well as the mandibles and bone rostral to maxillae; the remaining skull was placed in Iscove's Modified Dulbecco's Medium (IMDM, Sigma Aldrich). The meninges were removed from the skull cap using fine forceps and visualization under a light microscope. Meninges were digested for 20 minutes at 37 °C with 1.4 U/mL of Collagenase VIII (Sigma Aldrich) and 35 U/mL of DNAse I (Sigma Aldrich) in IMDM. Following digestion, the tissue was gently pressed through a 70 µm mesh cell strainer (Falcon). The flow-through material was centrifuged at 450 x g at 4 °C for 4 minutes. Spleens were processed in a manner similar to what was used for the meninges with the exception that we performed an additional lysis step with ammonium-chloride-potassium (ACK) Lysis Buffer (Quality Biological) prior to staining.

Instrument: Cells were analyzed on a Cytek Aurora (Cytek Biosciences).

Software: FlowJo v10.8.1 was used to analyze abundances of cell populations.

Cell population abundance: Cells collected from each sample type were resuspended in ice-cold FACS buffer (2 mM EDTA, 25 mM HEPES, 1% BSA in 1X PBS) and stained for extracellular markers at 1:300 dilution. Dead cells were excluded using Zombie NIR fixable Viability kit.

Gating strategy: See Supplementary Information for gating strategies.

☒ Tick this box to confirm that a figure exemplifying the gating strategy is provided in the Supplementary Information.
